# Beyond contacts: The important role of the support region in protein complex assembly

**DOI:** 10.1002/pro.70470

**Published:** 2026-01-20

**Authors:** Tom Miclot, Stepan Timr

**Affiliations:** ^1^ Department of Computational Chemistry J. Heyrovsky Institute of Physical Chemistry, Czech Academy of Sciences Prague Czech Republic; ^2^ Present address: Université Paris Cité Paris France

**Keywords:** interface, non‐bonded interactions, protein region, protein–protein interactions, stable complexes, transient complexes

## Abstract

Protein–protein interactions (PPIs) are fundamental to nearly all cellular processes; however, elucidating the principles governing protein association into complexes remains a significant challenge. Previous studies have shown that protein–protein interfaces can be partitioned into distinct regions—core, rim, and support—that differ in hydration and residue composition. Here, we present a detailed analysis of interactions occurring within each of these interface regions. Extending beyond simple residue proximity or atomic contacts, our analysis, facilitated by our open‐source software MICLOT, distinguishes among 18 different types of non‐bonded interactions and evaluates their dependence on the local region. Our results demonstrate that, despite its relatively low solvent accessibility prior to complex formation, the support region contains a significant number of interactions stabilizing the interface. In particular, we find that the support promotes specific residue pair interactions, including hydrogen bonds, aromatic–aromatic and arginine stacking interactions, as well as van der Waals contacts. Furthermore, we observe that the size of the support region positively correlates with overall interface stability, and we detect differences in residue partitioning between the support and rim regions when comparing stable and transient complexes. Additionally, we introduce innovative strategies inspired by natural language processing to analyze the diversity and co‐occurrence of interacting residue pairs, enabling detailed comparison of internal local organization within stable and transient interfaces. Our analysis reveals that, although pair diversity in the core and support regions falls between the interior and rim, the core and support regions exhibit interior‐like pair organization in stable interfaces. Collectively, our findings emphasize the crucial role of the local environment within an interface in shaping residue interactions and underscore the support region as a key contributor to protein complex stability.

## 1 INTRODUCTION

Protein–protein interactions (PPIs), physical associations between two or more protein molecules, result in the formation of higher‐order assemblies, which can involve either distinct proteins or multiple copies of the same protein chain. The formation of these assemblies, held together by PPIs, is crucial for the function of many proteins, including structural proteins (Wagstaff & Löwe, 2018) and numerous enzymes (Narayanaswamy et al., 2009; Noree et al., 2019; Park & Horton, 2019; Petrovska et al., 2014), as well as for a wide range of cellular processes (Garcia Seisdedos et al., 2022; Garcia‐Seisdedos et al., 2017; Headd et al., 2007; McPartland et al., 2018; Zhang et al., 2023).

The strength of PPIs is a key determinant of the functional roles of protein assemblies, and these interactions can be either transient or stable in nature. For instance, PPIs involved in cell signaling pathways are typically transient, allowing for reversible protein association and repeated signal transduction (Sweetlove & Fernie, 2018). In contrast, antibody–antigen complexes require high stability to effectively neutralize pathogens (Nooren, 2003). Understanding the differences between transient and stable PPIs remains a significant challenge, especially as the complexity of the system increases (Grassmann et al., 2024). PPI stability depends on multiple factors, including external conditions (Stein et al., 2009), interface size (Ansari & Helms, 2005), and the physicochemical properties of the interface, such as residue composition (Jayashree et al., 2019; Swapna et al., 2012).

Within both stable and transient protein–protein interfaces, a complex network of non‐bonded interactions between amino acid residues forms, playing a crucial role in protein assembly and complex stabilization (Adhav & Saikrishnan, 2023; Hou et al., 2018). For example, recent work has highlighted the prevalence of electrostatic interactions in transient interfaces (Grassmann et al., 2023). Given the importance of PPIs, a variety of methods and software tools have been developed to investigate them, including approaches that characterize residue–residue interaction types using geometric descriptors (Fonseca, n.d.), analyze interaction networks (Sora et al., 2023), evaluate interaction potentials using physics‐based models (Grassmann et al., 2023; Hou et al., 2018), use statistical potentials to predict residue pair proximity (Li et al., 2024), and pairwise residue contact propensities (Villegas & Levy, 2022), and apply machine learning to examine and validate interfaces formed in protein complexes (Ovek et al., 2024).

Geometric and physicochemical complementarity are prerequisites for protein interactions and complex formation (Chothia & Janin, 1975; Conte et al., 1999; Desantis et al., 2022; Gainza et al., 2023; Kuroda & Gray, 2016; Li et al., 2013; Milanetti et al., 2021). To describe the internal organization of protein–protein interfaces, the core/rim model (Chakrabarti & Janin, 2002) has been widely adopted. According to this model, interfaces contain a buried region—the core—which is predominantly hydrophobic and contributes significantly to complex stability. As a result, the core is particularly sensitive to mutation (Bahadur & Zacharias, 2008; Clackson & Wells, 1995; Janin et al., 2008). In contrast, the more hydrated rim region is more polar or charged, less sensitive to mutations, and responsible for specific recognition between proteins (Bahadur & Zacharias, 2008; Chakrabarti & Janin, 2002; Grassmann et al., 2023; Janin et al., 2008). Consistent with the greater role of the hydrophobic, shape‐complementary core on interface stability and the lesser role of the polar rim, a recent study (Desantis et al., 2022) found that the stability of protein complexes correlates strongly with van der Waals interaction energies rather than with Coulombic interaction energies. In addition to the core and rim, some studies have identified a third region, the support, which comprises parts of the interface that are already largely buried prior to complex formation and become further buried upon complex formation. The support has been shown to contribute to binding affinity: mutations in this region can significantly impact complex stability (Livesey & Marsh, 2022), and pathogenic mutations are notably enriched here (Petukh et al., 2015). The work of Levy (2010), which proposed a simple definition of the core, rim, and support based on residue hydration and its changes (Levy, 2010), showed that the sizes of these regions scale with the total interface size. Moreover, the amino acid composition of the rim was found to be almost identical with that of the protein surface; the core was intermediate between the surface and the interior; and the composition of the support closely resembled that of the protein interior (Levy, 2010). Thus, the support can be understood as the interior‐like part of a protein–protein interface. However, a detailed characterization of the interactions formed within the support region remains lacking.

In this work, we characterize residue interactions within the distinct regions that compose a protein–protein interface. We introduce the Molecular Interaction anaLysis toOlkiT (MICLOT), a robust Python package capable of identifying a wide variety of non‐bonded interaction types and their subtypes. By applying this tool to curated benchmark collections of high‐quality structures—including both stable and transient complexes—we identify key differences in interaction patterns and their context‐dependent properties within the core, support, and rim regions of the interface. Our analysis reveals that, although the support region is largely buried prior to complex formation, it hosts a substantial number of interactions that contribute to complex stabilization. Furthermore, we observe that the support region becomes more prominent in stable interfaces, often at the expense of the rim, suggesting a functional interplay between these interface regions, traditionally considered to be of secondary importance to interface formation.

## 2 RESULTS

Our study utilizes a comprehensive dataset comprising 413 protein hetero‐complexes, compiled from multiple databases and filtered by UniprotKB accession numbers to eliminate redundancy. To ensure high data quality, only structures with a resolution of 2.5 Å or better were included. The dataset is categorized into three types of complexes based on binding affinity: stable (permanent), transient, and intermediate, following a standard definition (Grassmann et al., 2023; La et al., 2013) (see Section 4). Further details regarding the dataset and methodology are provided in Section 4 and Supporting Information. Interface regions are defined according to the nomenclature proposed by Levy (2010) and Kastritis et al. (2014). In this scheme, the core consists of solvent‐accessible residues that become buried upon complex formation, the rim includes residues that remain largely solvent‐accessible before and after binding, and the support comprises residues that are already largely buried prior to complex formation and become even more buried upon complex formation (see Section 4 for further details).

### 2.1 Support grows with interface stability

Previous work has reported a correlation—although relatively weak—between binding affinity and the amount of surface area buried at the interface (Chen et al., 2013). In contrast, our dataset does not exhibit any discernible correlation between binding affinity and total interface area (see Figures S5 and S6), as quantified by buried surface area (see Section 4). This lack of correlation may be attributed to the emphasis in our dataset on high‐resolution structures and the lower representation of protein–peptide complexes, which comprise the majority of low‐affinity cases as indicated by Chen et al. (2013). Nonetheless, our findings are consistent with those of Chen et al. (2013) in that we also observe considerable overlap in interface areas between transient and stable complexes. Therefore, interface area alone is insufficient to clearly distinguish between stable and transient complexes (Figure S5). In fact, the analysis suggests that the relationship between interface size and affinity does not show a straightforward scaling—high‐affinity complexes do not necessarily require large interfaces. However, caution is needed when generalizing this observation. The dataset includes only protein complexes with available structures and measured binding affinities, likely excluding many high‐affinity partners that are difficult to purify or are otherwise underrepresented. Consequently, the current findings apply to the available data and experimental methods and may differ for undiscovered or less tractable protein complexes.

Furthermore, in agreement with Chen et al. (2013), we do not detect a strong relationship between the global chemical composition of the interface and complex stability—aside from an increased occurrence of leucine by 3.0 (95% CI [4.3, 1.7]) percentage points (pp) and a decreased presence of glycine by 2.6 [1.3, 3.8] pp, serine by 2.8 [1.7, 3.9] pp, and aromatic residues such as tyrosine (by 3.3 [2.1, 4.4] pp) in transient interfaces (Figure S8).

To compare the sizes of the core, support, and rim regions in our dataset, we calculated the areas of these individual regions based on their buried surface areas. Consistent with previous findings (Levy, 2010), we find that, on average, approximately 60% of the buried surface area corresponds to the core, 30% to the rim, and 10% to the support (Table S1 and Figure S7). When comparing stable and transient interfaces (see Figure S7), we observe an increase in both the absolute area and the relative proportion of the support region in stable complexes (probability of superiority: 0.64 [0.55, 0.73]). In contrast, the rim region exhibits a decreasing trend (probability of superiority: 0.38 [0.29, 0.47]). These results suggest a shift in the partitioning of interfaces from more hydrated regions (i.e., the rim) toward less hydrated ones (i.e., the core and support) as stability increases.

**FIGURE 1:**
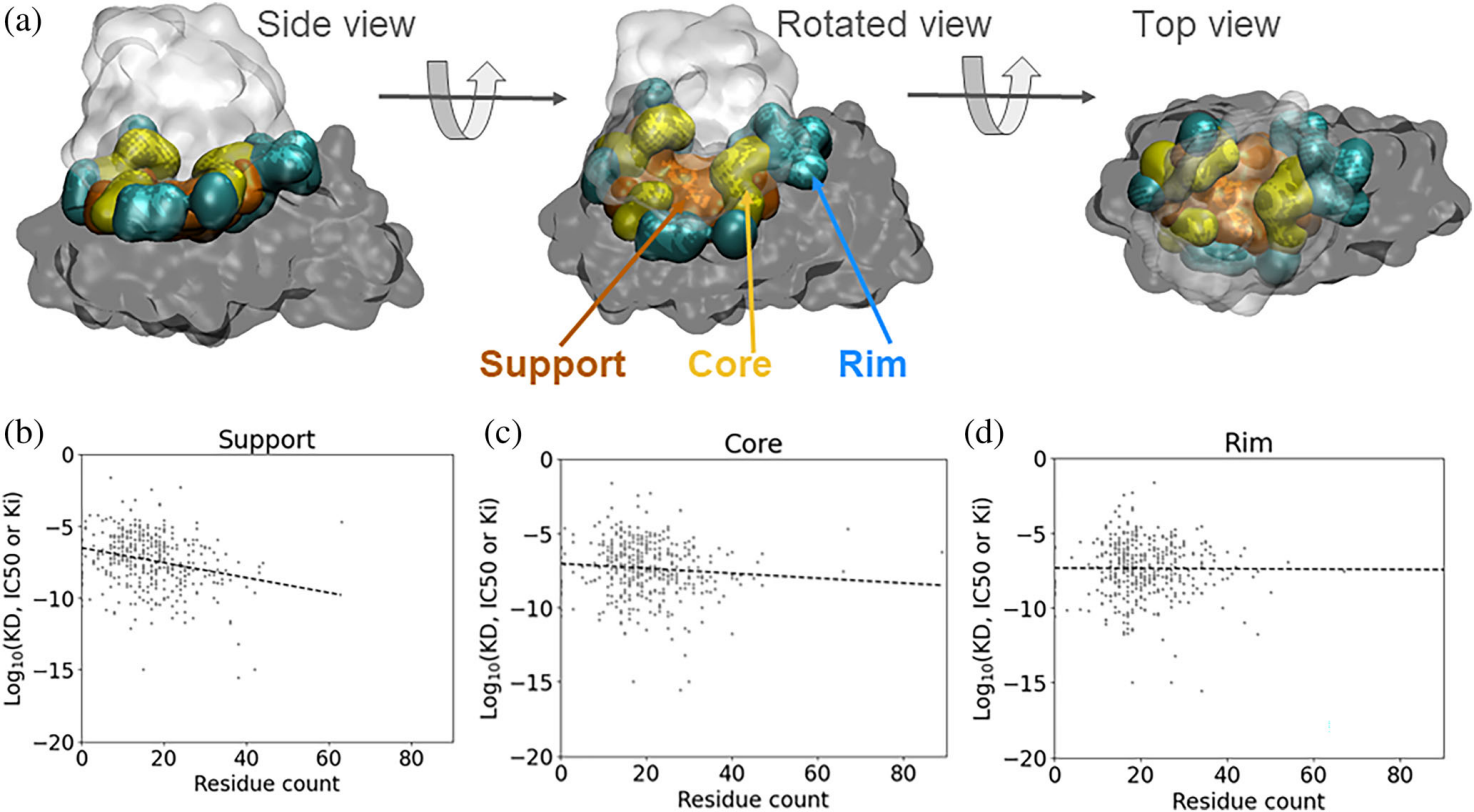
(a) Example of a protein interface, highlighting the distinct regions defined according to Levy (2010): The core as a solvent‐accessible region that becomes solvent‐inaccessible upon complex formation, the rim as a solvent‐accessible region that remains largely solvent‐accessible, and the support as a region formed by residues that are largely solvent inaccessible both in the unbound structures and in the complex (see Section 4 for the mathematical definition). Residues from each of these interface regions are highlighted on the dark‐gray chain. In addition, we define the rim non‐interacting surface (rim NIS) as the part of the rim that shows a less than 5% decrease in relative accessible surface area upon complex formation; see Methods. Binding affinity of the complex as a function of the number of residues forming the (b) support, (c) core, and (d) rim regions. More negative values indicate stronger binding. A linear regression using ordinary least squares yielded the best‐fit line y=−0.06x−6.29 ($R^{2}=0.086$ and $P=2.01\cdot 10^{-9}$) for the support, y=−0.02x−6.85 ($R^{2}=0.014$ and P=0.02) for the core, and y=−0.01x−7.24 ($R^{2}=0.001$ and P=0.6) for the rim region.

The support, already buried prior to complexation, contributes only modestly to the total surface area buried upon formation of the protein–protein interface. However, when we shift our perspective and consider its size in terms of the number of constituent residues, the relationship between support size and binding affinity becomes more apparent (see Figure 1). Despite considerable variability in the data, the overall trend suggests that larger support regions are associated with stronger binding affinities $\left(R^{2}=0.086\right)$.

### 2.2 Distinct physicochemical composition of interface regions in stable and transient complexes

**FIGURE 2:**
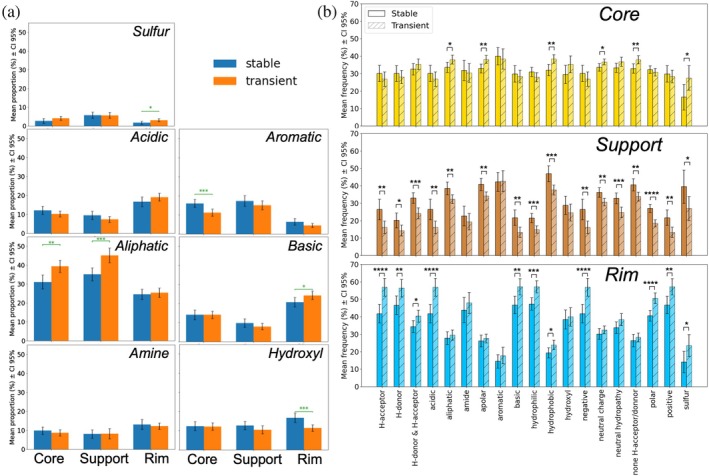
(a) Chemical composition of the different regions of stable and transient interfaces. (b) Stable and transient interfaces show differences in the partitioning of residue classes into interface regions. The mean values were computed across stable and transient complexes, respectively. The standard error of the mean with an appropriate Student's t coefficient for a 95% confidence interval was used to estimate uncertainty. Significance of differences between stable and transient complexes was assessed using Welch's *t*‐test. Statistical significance is denoted as follows: 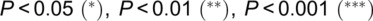, 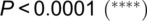. Significance of differences between regions—reported in the text—was assessed using paired *t*‐test. 95% confidence intervals for the mean differences reported in the text were obtained using the *t*‐tests.

To describe the physicochemical composition of individual regions, we performed a compositional analysis analogous to that presented by Levy (2010), but using the physicochemical residue classification established by Pommié et al. (2004) in place of standard residue names. Our analysis reveals pronounced differences between the core and the support regions on one hand, and the rim on the other (see Figures 2a and S9–13). As expected, the rim contains a larger percentage of charged residues than either the core (by 7 [3, 10] pp for positive and by 5 [1, 8] pp for negative residues in stable interfaces; see Figure S10) and support (by 11 [8, 14] pp and 7 [4, 10] pp, respectively), along with an increased presence of polar and hydrophilic residues (by 21 [16, 26] pp and 25 [21, 30] pp, respectively, compared with the support in stable interfaces; Figures S9 and S11). In contrast, it includes substantially fewer aromatic and aliphatic residues (by 11 [8, 14] pp and 11 [6, 15] pp, respectively, compared with the support in stable interfaces; Figure 2a). Notably, the support region exhibits the highest proportion of apolar and hydrophobic residues (49 [45, 53] % and 39 [35, 42] %, respectively, in stable interfaces; Figures S9 and S11), making its composition resemble that of the protein interior, as previously reported by Levy (2010).

We find that, on average, the rim region of transient interfaces is even more enriched in positively charged residues than that of stable interfaces (by 3.6 [0.3, 6.8] pp; see Figure S10). Conversely, the core and support of transient interfaces contain a higher proportion of apolar (by 10 [5, 15] pp and 12 [7, 18] pp, respectively) and hydrophobic residues (by 12 [7, 17] pp and 14 [8, 19] pp, respectively) compared with their counterparts in stable interfaces (Figures S9 and S11). This seemingly counterintuitive trend may be partially attributed to an increased presence of aliphatic residues in the core and the support of transient interfaces (by 8 [3, 13] pp and 10 [5, 15] pp, respectively; see Figure 2a), occurring at the expense of aromatic residues such as tyrosine, which is classified as a polar residue with neutral hydropathy. Additionally, the core and support of transient interfaces contain fewer residues whose side chains can form hydrogen bonds (by 11 [5, 16] pp and 13 [7, 18] pp, respectively; see Figure S12), which may further limit the stability of these complexes.

In several aspects—such as the decreased frequency of aliphatic residues, along with the lower proportions of apolar and hydrophobic residues and those with side chains that cannot form hydrogen bonds—the average composition of the support region in stable complexes more closely resembles that of the rim than it does in transient complexes. This observation further supports the idea that the key difference between stable and transient interfaces lies not in the overall residue composition, but in the local environment in which these residues are found. This hypothesis is reinforced by an analysis of residue class partitioning across different interface regions (Figure 2b). Specifically, we find that stable and transient interfaces differ in the average distribution of a number of residue classes between the support and rim regions. For instance, stable interfaces exhibit an increased partitioning of both positively and negatively charged residues into the support region in stable interfaces (by 8 [3, 14] pp and 10 [3, 17] pp, respectively), at the expense of the rim. In contrast, the partitioning of residue classes into the core and the rim non‐interacting surface (NIS) shows no major differences between stable and transient interfaces.

The observed interplay between the support and rim regions (Figure 2b) highlights their potential as key indicators for evaluating interface type. Consequently, analyzing their physicochemical properties and the interactions they mediate may be particularly informative for distinguishing between stable and transient protein interfaces.

Finally, to provide a more detailed view of how individual amino‐acid species contribute to the various interface regions, we evaluated the “stickiness” of these residues (Villegas & Levy, 2022) for each region. “Stickiness” is a statistical free‐energy scale that quantifies how overrepresented an amino acid is at protein–protein interfaces relative to water‐exposed surfaces. Specifically, it compares the fractional contribution of each amino‐acid type to the interface area with its contribution to the solvent‐accessible protein surface area (see Figure S20 and Section K in Supporting Information). The core and the support regions exhibit similar trends (see Figure S21), with hydrophobic residues showing positive stickiness values and hydrophilic residues typically showing near‐zero or negative values. These results are consistent with those reported by Villegas and Levy for interface residues undergoing significant changes in solvent‐accessible surface area upon interface formation. With a few exceptions, such as tyrosine, phenylalanine, and methionine, stickiness values in the rim tend to be close to zero for most residues, supporting the view that the rim region closely resembles the protein surface.

### 2.3 Support hosts more interactions between protein chains than rim

Non‐bonded interactions between residues from different protein chains play a critical role in stabilizing protein–protein interfaces and mediating their specificity. Consequently, a detailed characterization of these interactions is essential for understanding the factors that govern interface stability. Our analysis goes beyond simple assessments of residue proximity or side‐chain contacts. Instead, it identifies diverse types of non‐bonded interactions and evaluates their dependence on the local environment within an interface.

**FIGURE 3:**
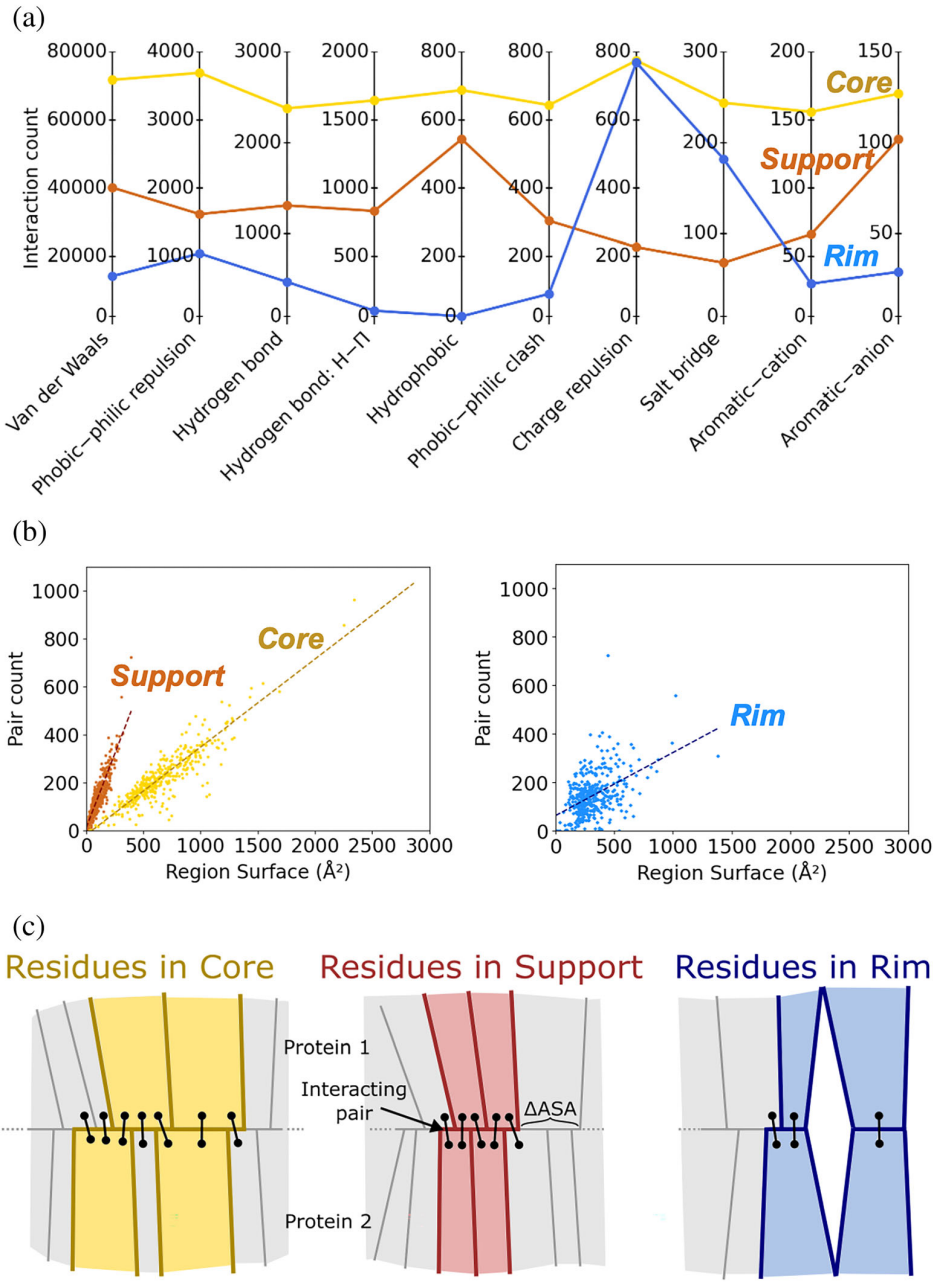
(a) Total interaction counts detected in the core (yellow), support (brown), and in the rim (blue), considering the whole dataset. Only interactions with at least 20 occurrences in each region for the dataset are shown. (b) Scaling of the number of interacting residue pairs with the buried surface area in each region: Core (yellow), support (brown), and rim (blue). Note that the rim and rim (NIS) were combined for this analysis. The data were fitted using a linear regression model yielding: y=0.37x−18.25 ($R^{2}=0.854$ P<0.0001) for the core, y=1.24x+11.10 ($R^{2}=0.816$ P<0.0001) for the support, and y=0.26x+62.17 ($R^{2}=0.234$ P<0.0001) for the rim. (c) Schematic representation of the environment present in each region. Residues positioned more centrally (i.e., in the core and support) are likely to have more neighbors than those located near the periphery (rim).

Focusing on interactions where both residues belong to the same interface region, we find that, with the exception of salt bridges and charge repulsions, the total number of such interactions detected in the support region exceeds that in the rim (see Figure 3a and Table S3). Thus, despite contributing less to the buried surface area (see Table S1 and Figure S7), the support region hosts more stabilizing interactions than the rim.

When two residues are engaged in at least one interaction, they form an interacting pair. The number of interacting pairs bridging the two protein chains (Table S2) scales as approximately three times the residue count within the core region, roughly twice the residue count within the support region, and at a one‐to‐one rate within the rim (see Figure S17A). Even when interacting pairs connecting two different regions are considered (see Figure S17B–D), the hierarchy (core > support > rim) remains preserved.

Since the regions differ in their contribution to the surface area buried upon complex formation (see Table S1 and Figure S7), we investigated how the total number of interacting pairs scales with the buried surface area in each region (see Figure 3b). Here, interacting pairs where both residues belong to the same region are counted twice as they effectively cross the region's buried surface area twice—once for each protein chain. Consistent with contributing the lowest buried surface area while still exhibiting a relatively high pair count, we find that the support shows the steepest scaling of the three regions (Figure 3b).

The relatively high number of interacting residue pairs in the support region despite its low contribution to the buried surface area can be explained by a more central location of these residues within the interface compared with the rim residues. Residues positioned more centrally are likely to have more neighbors than those located on the periphery (see Figure 3c).

Taken together, the results for the support region are consistent with a densely packed and highly connected arrangement, in which residues are positioned close together and engaged in local interactions. These results highlight the substantial contribution of the support region to bridging the two protein chains within the complex.

### 2.4 Support promotes specific interactions between residue pairs

Next, we asked whether two residues that form an interacting pair across distinct protein chains engage in the same types of interactions regardless of their interface region. To determine whether residue–residue interactions are region‐dependent, we analyzed each interaction type individually and calculated the average number of interactions per residue pair that is capable of forming that specific interaction. Note that, to obtain a clearer picture, we focused only on pairs in which both residues belong to the same interface region.

**FIGURE 4:**
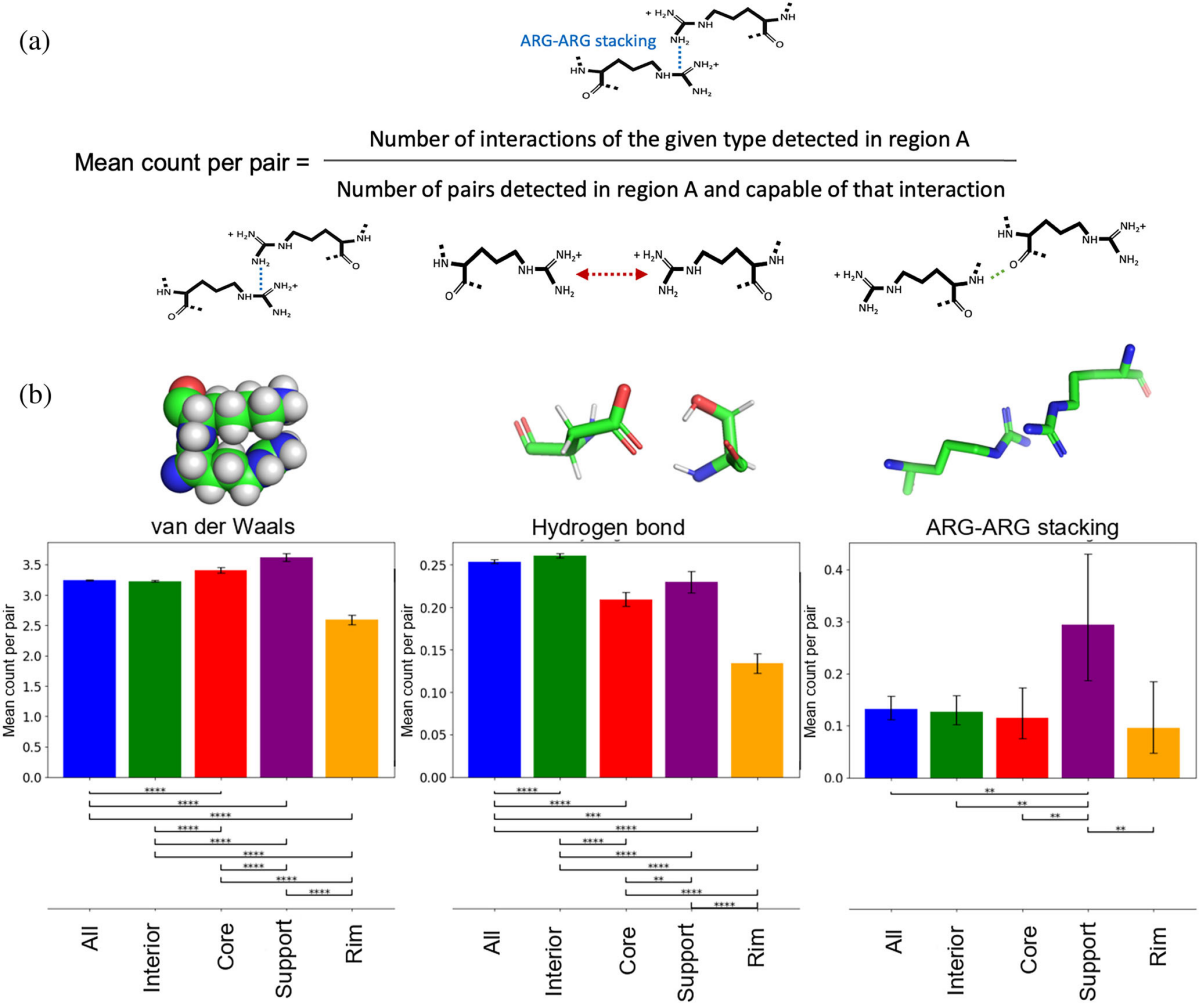
(a) Definition of the mean count per pair as the average interaction count by any residue pair capable of forming that specific interaction and located in a given protein region. (b) Mean count per pair, without considering the stability of the complexes. For binary interactions (i.e., present: 1, absent: 0), such as ARG–ARG stacking, the Wilson score interval Wilson (1927) at the 95% confidence level was used to estimate the uncertainty in the average value. To assess whether differences in averages between two regions are statistically significant, the two‐proportion *z*‐test was applied. For other, non‐binary interactions (e.g., van der Waals or hydrogen bonds), the standard error of the mean with an appropriate Student's t coefficient for a 95% confidence interval was used to estimate uncertainty. Significance of differences between regions was tested using Welch's *t*‐test. Statistical significance is denoted as follows: 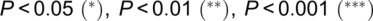, 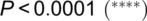. 95% confidence intervals for the effect sizes reported in the text were obtained using the Newcombe method for binary interactions and using Welch's *t*‐test for non‐binary interactions.

We find that the likelihood of an interacting residue pair engaging in a specific interaction type varies depending on the location of the pair within the interface (see Figures 4 and S19). Notable regional differences are observed for regular hydrogen bonds, arginine–arginine stacking interactions, and van der Waals interactions (see Figure 4). These findings indicate that regional differences in inter‐chain interaction counts are not solely due to varying numbers of bridging residue pairs, but also reflect distinct interaction preferences of the residue pairs in each region.

Interacting residue pairs in the support region exhibit an enhanced propensity for several interaction types (see Figure S19). Compared with the rim, the support more strongly promotes arginine–arginine stacking interactions and salt bridges (Table S4), increasing their probabilities by 0.20 (95% CI [0.06, 0.34] and 0.13 [0.01, 0.22], respectively). It also increases the average number of regular hydrogen bonds by 0.10 [0.08, 0.11] and H–π hydrogen bonds by 0.12 [0.02, 0.21]. Hints of increased propensity are also observed for additional interaction types, though these trends are accompanied by higher uncertainty due to limited statistics.

Interestingly, certain interactions are favored in the support even more than in the core or interior regions (see Figure S19). Specifically, aromatic–aromatic, aromatic–anion, and arginine–arginine stacking interactions become even more likely than in either the core (by 0.09 [0.02, 0.17], 0.07 [0.01, 0.14], and 0.18 [0.06, 0.32], respectively) or the interior (by 0.13 [0.07, 0.19], 0.11 [0.06, 0.16], and 0.17 [0.06, 0.31], respectively). In addition, the propensity for regular hydrogen bonds is higher by 0.02 [0.01, 0.03] than in the core, and the propensity for aromatic–cation interactions is larger by 0.13 [0.07, 0.19] than in the interior. Conversely, a smaller number of interaction types are disfavored in the support compared with the core (H–π hydrogen bond by 0.08 [0.02, 0.15], aromatic S/Se interaction by 0.11 [0.01, 0.22]) and the interior (regular hydrogen bonds by 0.03 [0.02, 0.04]).

Overall, the elevated propensity for multiple interaction types points to a high degree of complementarity in the support region, where the side chains of residues from the two protein chains form a densely packed arrangement. Thus, despite its modest contribution to the total buried surface area of the interface, the support plays a significant role in coupling the two protein chains through its high internal complementarity.

The dense packing of the support is further evidenced by its highest propensity for van der Waals contacts among all interface regions (see Figures S19 and S18), as well as by the higher frequency of close‐range interactions—termed charge clashes—between the side chains of like‐charged residues, compared with the rim (by 0.03 [0.01, 0.07]; see Figure S19). In contrast, the more loosely packed rim more frequently exhibits “charge repulsions” (by 0.07 [0.04, 0.12]), where like‐charged side chains are positioned farther apart. A similar trend is observed for interactions between hydrophobic and hydrophilic residues: the support promotes close contacts—termed hydrophobic–hydrophilic clashes—by 0.07 [0.05, 0.10] whereas the rim favors more distant arrangements, referred to as hydrophobic–hydrophilic repulsions by 0.08 [0.06, 0.11] (see Figure S19).

Although the core region is somewhat less densely packed than the support—as indicated by a lower number of van der Waals interactions by 0.2 [0.1, 0.3]—it still promotes several interaction types more strongly than the rim (see Figure S19). These include regular hydrogen bonds (by 0.08 [0.06, 0.09]), H–π hydrogen bonds (by 0.2 [0.1, 0.3]), salt bridges (by 0.08 [0.01, 0.15]), and van der Waals contacts (by 0.8 [0.7, 0.9]). Compared with the protein interior, the core shows an increased propensity for aromatic–cation (by 0.12 [0.08, 0.16]) and aromatic–S/Se interactions (by 0.10 [0.02, 0.17]), H–π hydrogen bonds (by 0.08 [0.04, 0.13]), as well as van der Waals interactions (by 0.18 [0.13, 0.23]). Conversely, the likelihood of forming regular hydrogen bonds and S/Se chalcogen or hydrogen bonds tends to be lower in the core than in the interior (by 0.05 [0.04, 0.06] and 0.02 [0.01, 0.03], respectively).

Taken together, these observations suggest that the local environment within an interface significantly influences how two residues interact. Therefore, partitioning the interface into core, support, and rim regions emerges as a key factor in governing overall interface stability.

### 2.5 Interacting pair diversity in core and support is between interior and rim

Our interaction analysis enables evaluation of the local arrangement of interacting residue pairs. Unlike previous approaches, which often relied on arbitrary distance thresholds to define interacting pairs, our method employs precise geometric criteria—implemented in our MICLOT software—to determine whether two neighboring residues truly form an interaction.

As expected, larger interfaces tend to contain more interacting residue pairs and interactions overall. The distributions of interaction and pair counts across protein–protein interfaces closely mirror the distribution of interface sizes (see Figures S16 and S4). Indeed, these counts scale linearly with interface size (see Figures S14 and S15). In contrast, the number of distinct interaction types and pair types shows a weaker dependence on interface size. It is important to note that the term *interaction type* refers to a non‐bonding interaction, such as a salt bridge or van der Waals interaction. In contrast, a *pair type* is defined by the specific residues involved in the pair, for instance, ARG‐HIS or PHE‐TRP. The distributions of interaction and pair types are bell‐shaped, with most interfaces comprising between 6 and 13 interaction types and between 40 and 95 pair types. These findings suggest that a moderate level of diversity in interaction and pair types is a characteristic feature—and likely a requirement—for effective protein complex formation.

**FIGURE 5:**
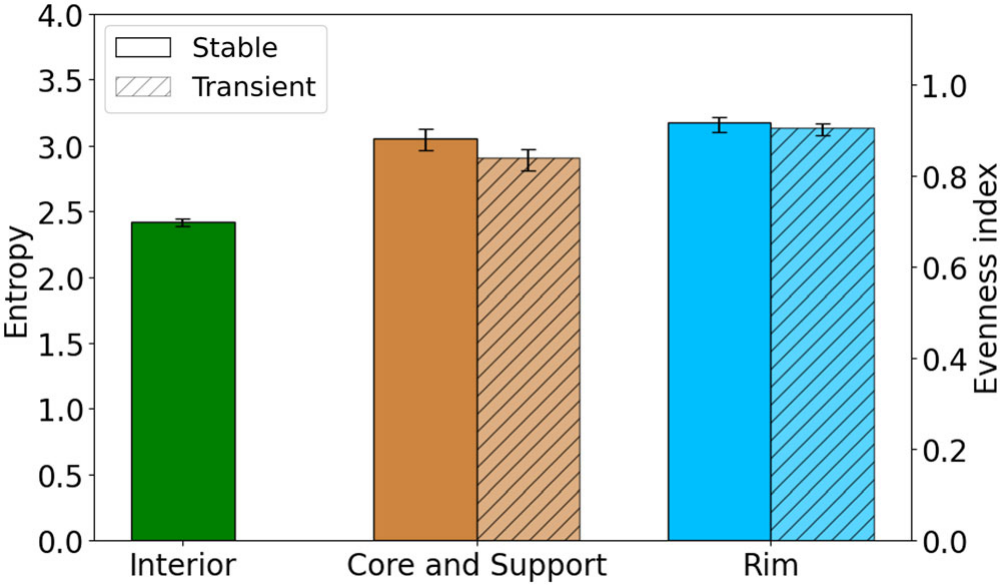
Shannon entropy and evenness index of interacting pair classes, defined by a combination of polarity and charge, demonstrate a relationship between the hydration level of an interface region and the diversity of its pair composition extracted from the dataset. The rim exhibits greater pair diversity than the core and support, which in turn show higher diversity than the protein interior. The error bars represent 95% confidence intervals derived from bootstrapping the dataset. Bootstrapping was also used to estimate the 95% confidence intervals for the differences reported in the text.

The analysis of Shannon entropy and evenness indices (see Figure 5), as described in Section I in Supporting Information, reveals that the diversity of interacting pairs, represented in simplified form based on their charge and polarity properties (see Tables S5 and S6), increases with the hydration level of the interface region. Notably, the rim exhibits the highest evenness values, indicating a highly diverse population of pairs extracted from the dataset (Figure 5). In contrast, the core and support—analyzed together to improve statistical robustness—show slightly lower evenness values (by 0.03 [0.01, 0.06] in stable interfaces and by 0.06 [0.04, 0.09] in transient interfaces; see Figure 5), though these still exceed those observed in the protein interior (by 0.18 [0.16, 0.21] in stable interfaces and by 0.14 [0.11, 0.16] in transient interfaces), which is characterized by a more uniform pair composition. These diversity metrics also provide insight into differences between stable and transient interfaces. Specifically, the core and support regions of stable interfaces harbor a slightly less homogeneous interacting pair population compared with their counterparts in transient interfaces (by 0.18 [0.16, 0.21] in stable interfaces and by 0.14 [0.11, 0.16] in transient interfaces; see Figure 5), suggesting that a greater diversity of pair types may be necessary to promote stable binding. In contrast, the rim regions of both stable and transient interfaces display similar levels of heterogeneity.

### 2.6 Core and support exhibit interior‐like pair organization in stable interfaces

To gain deeper insight into local interface architecture, we investigate whether certain residue types are more likely to co‐occur and form interacting pairs, and to what extent the observed pair type distribution reflects simple combinations of individual residue type frequencies. For this analysis, we employ mathematical tools inspired by natural language processing—specifically, term frequency–inverse document frequency (tf‐idf) (Luhn, 1957; Sparck Jones, 1972) and point‐wise mutual information (PMI) (Church & Hanks, 1989; Dagan et al., 1993; Niwa & Nitta, 1994). It is important to note that in our application, PMI values are solely based on our statistical data and do not serve as predictive measures. The methodological details are provided in Section 4 and in Section J in Supporting Information.

**FIGURE 6:**
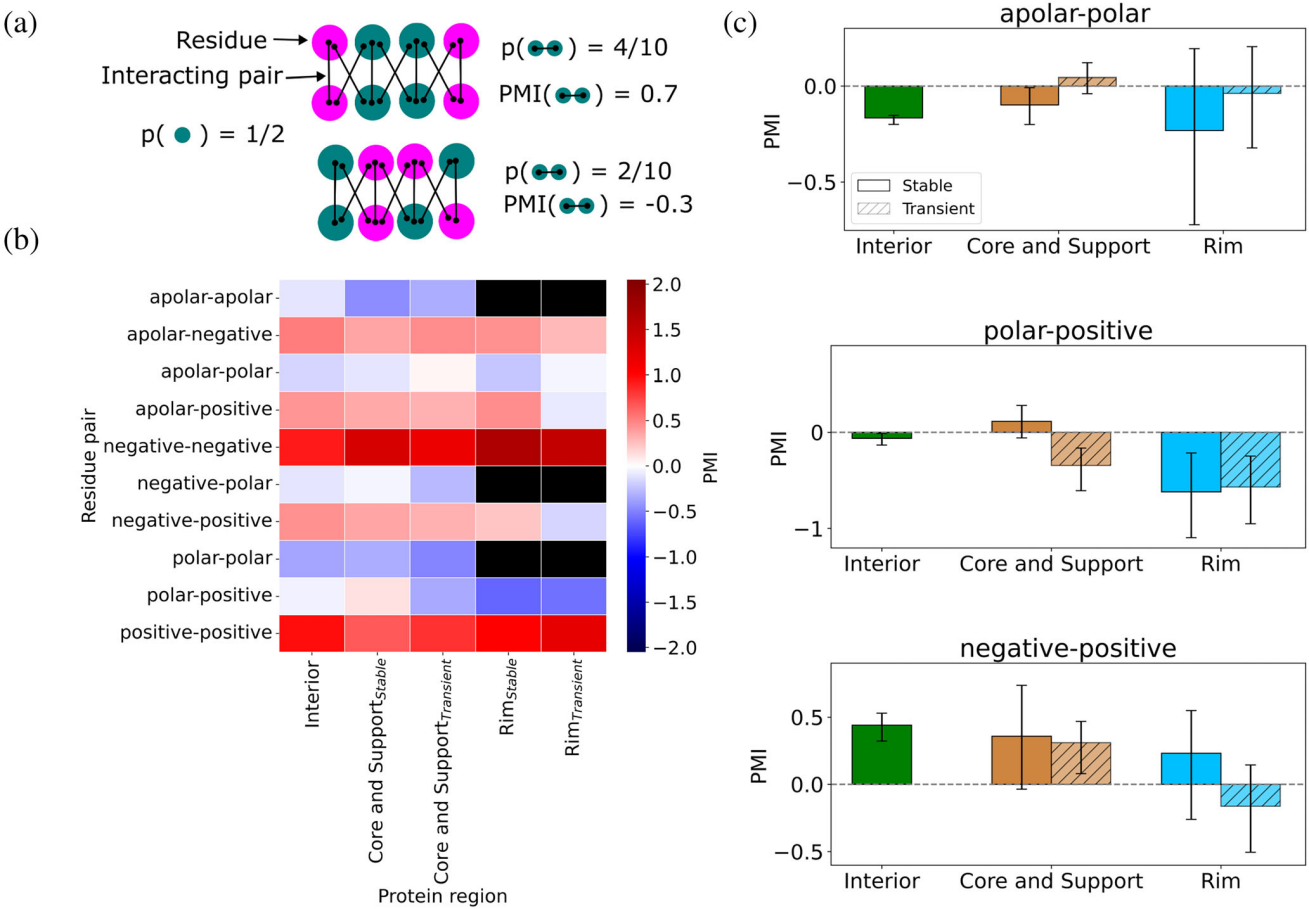
(a) Schematic illustrating how the PMI score evolves based on the relative frequency with which an interacting pair class occurs. Higher PMI values indicate pair classes that occur more frequently than expected by chance. (b) Co‐occurrence of residue classes forming an interacting pair is reported using the PMI score, following data filtering based on tf‐idf values below 1 (see Section J in Supporting Information). Removed values are indicated by black cells. Scores are presented as a heatmap, with color code corresponding to the PMI value, shown as a function of region. To improve statistical robustness, the core and support regions are analyzed together. (c) Trends in PMI scores for selected interacting pairs. The error bars represent 95% confidence intervals derived from bootstrapping the dataset. Bootstrapping was also used to estimate the 95% confidence intervals for the differences reported in the text.

We first apply tf‐idf to filter out pair types that appear only in a limited subset of structures within each interface region (Table S7). For the remaining pair types, PMI values reveal distinct co‐occurrence trends (see Figure 6 and Table S8). The PMI score quantifies the association between two residue types when forming an interaction, such that a high PMI value may occur even if the pair type is rare, provided the co‐occurrence is stronger than the baseline given by the product of the individual residue type frequencies. For example, the negative–positive pair type exhibits a relatively high PMI value (0.4 [0.3, 0.5]) in the protein interior (Figure 6b), indicating that oppositely charged residues—though relatively rare in this region—are likely to form a pair when present. In contrast, the apolar–apolar pair type, despite being frequent in the interior, has a PMI value close to zero (−0.10 [−0.12, −0.08], Figure 6b), suggesting a lack of strong mutual association. Note that comparisons of PMI values across different pair types should be interpreted with caution, since pair types capable of longer‐range interactions, such as charge repulsions, may exhibit higher PMI values because such interactions can occur even between residues that are not in close contact. In contrast, pair types restricted to short‐range interactions, and therefore requiring close contact, may tend to show lower PMI values. Despite this limitation, the PMI metric provides a useful tool to assess how the co‐occurrence of a given interacting pair type varies across interface regions and complex types.

For a number of pairs, the PMI values have large error bars, which precludes conclusive comparison between regions; a larger dataset would likely be required to obtain more discriminative results. However, certain pairs exhibit clear trends between stable and transient interfaces. In the core and support regions of stable interfaces, the “matching” polar–positive pair shows a significantly higher PMI (by 0.5 [0.2, 0.8]; Figure 6c) than in the corresponding regions of transient interfaces, where the value also becomes lower than in the interior (by 0.3 [0.1, 0.5]). In contrast, the PMI of the “mismatching” apolar–polar pair exhibits a low, interior‐like value in the core and support of stable interfaces while it is increased (by 0.2 [0.1, 0.3]; Figure 6c) in the core and support of transient interfaces. These results suggest an increased degree of internal organization—similar to that of the interior—in stable interfaces where the co‐occurrence of favorably interacting pairs is enhanced and the amount of “mismatches” decreases. Finally, while the PMI values of the negative–positive pair are comparable to that of the interior in most regions (Figure 6c), the PMI of the pair decreases by 0.6 [0.3, 1.0] in the rim of transient interfaces, consistent with a weaker complementarity of this region in transient interfaces.

## 3 DISCUSSION AND CONCLUSIONS

In this work, we presented a detailed analysis of interactions occurring within the core, support, and rim regions of protein–protein interfaces. Our results confirm that the core—an initially solvent‐exposed region that becomes buried upon complex formation—harbors the largest number of stabilizing interactions. In contrast, the more hydrated rim region contributes only modestly to inter‐chain coupling. Notably, our results highlight the important contribution of the support region. Although the residues in this interior‐like region are largely buried prior to complex formation—an observation that might suggest a limited role in inter‐chain interactions—they in fact participate in a substantial number of interactions linking the two protein chains. Moreover, the support region exhibits an even greater propensity than the core for certain interaction types, including aromatic–aromatic, aromatic–anion, and arginine–arginine stacking interactions, as well as hydrogen bonds. These findings indicate a high degree of structural and chemical complementarity within the support region. We note that defining regions by solvent accessibility does not necessarily yield a single connected area within an interface. On the contrary, in some cases, the regions consist of several distinct islands. For the core and the rim, this behavior has been shown to be characteristic of transient interfaces Luo et al. (2015). Thus, analyzing residue connectivity within each region may provide a useful means of characterizing local organization and complementarity within an interface. One might also ask whether the relatively strong contribution of the support region to protein–protein interactions arises from an induced‐fit mechanism, whereby surfaces of largely buried residues become exposed to interaction with the binding partner. However, based on our analysis, this does not appear to be the cause of the observed effect. We note that our analysis approximates free‐solution monomers using monomer structures extracted from the complexes (listed in Section L in Supporting Information). Therefore, the support region remains largely buried even in monomer structures isolated from the complex, that is, after any potential induced‐fit effects.

The relative hydrophobicity of the support region, along with its role in complementing interactions in the core, invites comparison with the O‐ring theory (Bogan & Thorn, 1998; Chakrabarti & Janin, 2002; Clackson & Wells, 1995; DeLano, 2002; Moreira et al., 2007; Thorn & Bogan, 2001). Also known as the “water exclusion” hypothesis, this theory suggests that less energetically significant residues form an O‐ring that facilitates complex formation by expelling water molecules from hotspot regions containing the most energetically significant residues (Fernández & Scott, 2003; Privalov et al., 2007). While the support region and the O‐ring represent distinct concepts, residues within the support region may nonetheless contribute, at least in part, to O‐ring formation.

Our results indicate that interface stability is not primarily dictated by its overall size or specific residue composition; indeed, stable interfaces do not differ dramatically in residue composition from transient ones. Instead, stability appears to be influenced by how residues are partitioned among distinct interface regions. In particular, we find that an increase in the size of the support region correlates with greater interface stability. Furthermore, for a number of residue‐ and interacting pair classes, their partitioning into the support grows at the expense of the rim in stable interfaces. This “interplay” between the support and rim highlights the critical role of the local environment within an interface in determining stability. These findings suggest that monitoring the properties of the support region may open up new avenues for predicting interface stability. Overall, while the core and support regions of stable interfaces exhibit higher heterogeneity in interacting residue pairs than the protein interior, they also display a degree of local organization similar to that of the protein interior, as demonstrated by our interacting pair co‐occurrence analysis.

The presented analysis was made possible by our newly developed MICLOT software, which has proven to be a powerful tool for identifying diverse interaction types, classifying interacting residue pairs, and characterizing their local interaction contexts within both stable and transient interfaces. We anticipate that MICLOT will be especially valuable for characterizing the variability of interface regions in structural ensembles from NMR and analyzing the temporal evolution of protein–protein interfaces in molecular dynamics simulations of macromolecular processes in the cytoplasm, such as the formation of dynamic enzyme assemblies. Ultimately, the detailed insights provided by MICLOT may aid in distinguishing between stable and transient interfaces (Bendell et al., 2014; La et al., 2013), as well as between different classes of protein–protein complexes. Looking ahead, MICLOT could be extended to analyze the positions of solvent molecules at the interface and indirect non‐bonded interactions—such as water‐mediated hydrogen bonds—which are also known to contribute to interface stabilization (Petukhov et al., 2004). Additionally, incorporating further physicochemical properties of amino acids (Lundblad, 2018) or molecular descriptors (Todeschini, 2009) could enhance the depth of the analysis or enable predictive modeling. To this end, MICLOT could be integrated within a hybrid framework alongside existing classification approaches, including physics‐based energy models (Grassmann et al., 2023; Hou et al., 2018), feature‐driven machine learning using structural and physicochemical descriptors (Vangone & Bonvin, 2015), or data‐driven deep learning methods that operate directly on sequence or three‐dimensional structural representations (Renaud et al., 2021; Wu et al., 2023).

Our analysis software used in this study, MICLOT, is freely available as open‐source and is accompanied by comprehensive documentation. The software can be accessed on GitHub at: https://github.com/TMiclot/MICLOT.

## 4 MATERIALS AND METHODS

### 4.1 Interaction types identified by the software

Below, we provide an overview of the interactions detected and analyzed in this study. Complete documentation of the MICLOT software, including the precise criteria used for interaction detection, is available in the GitHub repository https://github.com/TMiclot/MICLOT.

The following stabilizing non‐bonded interactions were analyzed: $n\to \pi^{*}$: Interaction between the carbon and oxygen atoms of two carbonyl groups (C=O) of the backbone. *C‐bond*: $n\to \sigma^{*}$ electron delocalization between a donor $C^{\mathrm{sp}3}$ carbon atom and an acceptor carbonyl oxygen atom. *Chalcogen bond*: Attractive interaction between a sulfur or a selenium atom and an electrophilic region (Aakeroy et al., 2019). *S/Se‐mediated hydrogen‐bond*: Hydrogen bond involving a sulfur or a selenium atom as a hydrogen donor or a hydrogen acceptor. *Hydrogen bond*: Attractive interaction of a hydrogen atom covalently bonded to an electronegative atom with another electronegative atom (Arunan et al., 2011). *H–π bond*: Attractive interaction of a hydrogen atom with the π part of an aromatic cycle. *Aromatic–aromatic*: Interaction between the π parts of two aromatic residues, or interaction between the π part of one aromatic residue and the quadrupole part of another, or interaction between the quadrupole parts of two aromatic residues. *Amino–π*: Interaction between the π part of an aromatic cycle and the amino group of a glutamine or an asparagine residue. *Aromatic–S/Se*: Interaction between a sulfur or a selenium atom and the π area or the quadrupole area of an aromatic cycle. *Aromatic–charge*: Positively (cation) or negatively (anion) charged amino acid residues interacting with the π area or the quadrupole area of an aromatic cycle. *Salt bridge*: Electrostatic interaction involving both a hydrogen bond and an ionic bond between two amino acid residues with opposite charges. *Van der Waals*: Interaction between atoms due to dipole‐induced dipole and dispersion forces (Minkin, 1999). *Hydrophobic*: Interaction between two close hydrophobic residues. *ARG–ARG stacking*: Stacking between two arginines. Please note that this type of interaction is excluded from the “charge clash” category in our analysis; see below.

*Repulsion* and *clash* refer to interactions between pairs of nearby residues that either share the same charge or differ in hydropathy. Accordingly, the identified repulsion/clash types include: anion–anion, cation–cation, and hydrophobe–hydrophophile. Two residues are in *repulsion* if their backbones are close but their side chains are far apart. In contrast, they are in *clash* if both their backbones and side chains are in close proximity. The presence of a repulsion or clash is generally unfavorable; however, its overall effect depends on nearby residues that may interact with the involved side chains.

Among all possible residue pairs in a system, only those with a $C_{\alpha }‐C_{\alpha }$ distance of 14 Å or less are retained for analysis. This distance threshold allows the identification of both short‐range and long‐range interactions. Because the software is able to identify multiple interaction types and subtypes, some interaction (sub‐)types were grouped for ease of analysis. In addition, alternative residues positions recorded in the structure file, if present, were not taken into account.

### 4.2 Dataset preparation

All complex structures were sourced from five validated databases: Protein–Protein Docking Benchmark 5.5 (Guest et al., 2021; Vreven et al., 2015), SAbDab (Dunbar et al., 2014), PPI4DOCK (Yu & Guerois, 2016), 3DComplex (Levy et al., 2006) v6.0 and PDBbind (Liu et al., 2015; Wang et al., 2004, 2005) v2020 (see Figure S1). For the latter, only structures containing two chains (dimers) were retained. In addition, all information concerning protein family, reference article (in PubMed format), classification, organism, presence of mutations, method used to solve the structure, resolution (if available), and the UniProtKB (Apweiler, 2004) number was obtained from the RCSB PDB (Berman, 2000) (see Figures S1, S2, and S3, and Section L in Supporting Information). While larger datasets have been reported in the literature (e.g., Gainza et al. (2020)), we selected a smaller dataset of complexes with known binding affinities and sufficiently high structural resolution, which was required for our detailed residue interaction analysis.

The first step of the selection process involved retaining only structures with known binding affinities. These values were taken from the database of origin; if unavailable, they were sourced from the PDBbind database. Next, only structures with a resolution below 2.5 Å or those determined by NMR were retained. Subsequently, the UniprotKB entry was used to remove redundancy: all duplicate structures were excluded to ensure that each entry in the dataset was unique. Structures with many incomplete residues, where too many atoms were absent to allow reconstruction, were also removed. The final dataset contained 413 protein hetero‐complexes. Please note that preselection was applied to structures from PDBbind and 3DComplex. For PDBbind, only dimeric complexes were retained. For 3DComplex, the selected structures were non‐identical, non‐homologous, and had distinct domain architectures for chains 1 and 2, as defined by SCOP and PFAM. For more details, see Sections A and B in Supporting Information.

### 4.3 Separation of complexes into stable and transient interfaces

**FIGURE 7:**
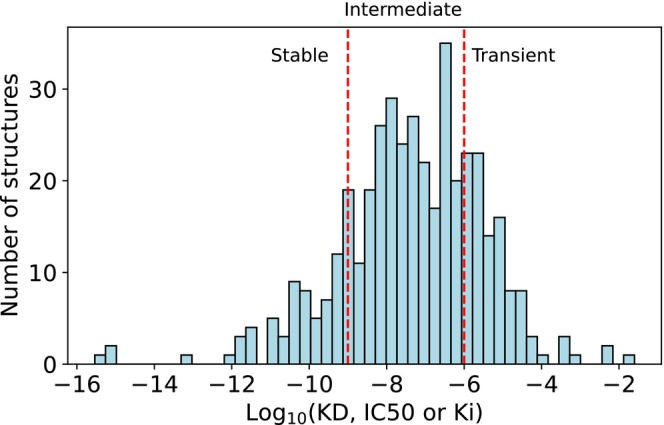
Distribution of complex affinities in the dataset. Of the total dataset, 94 complexes were identified as transient, with binding affinities (i.e., $\log_{10}\left(\text{Affinity}\right)$, where “Affinity” stands for $K_{D}$, IC50, or $K_{i}$) above −6, another 66 were classified as stable, with binding affinities below −9, while 253 complexes were categorized as intermediate, with binding affinities between −6 and −9.

Adopting an approach similar to that of Grassmann et al. (2023), we divided our dataset into distinct categories based on binding affinity, calculated as $\log_{10}\left(\text{Affinity}\right)$, where “Affinity” refers to $K_{D}$ (Vangone & Bonvin, 2015), IC50, or $K_{i}$, Complexes with a binding affinity above −6 were classified as transient, while those below −9 were designated as stable. Complexes with values between these thresholds were categorized as intermediate. Figure 7 shows that the majority of structures in the dataset were classified as intermediate.

### 4.4 Identification of protein regions

**FIGURE 8:**
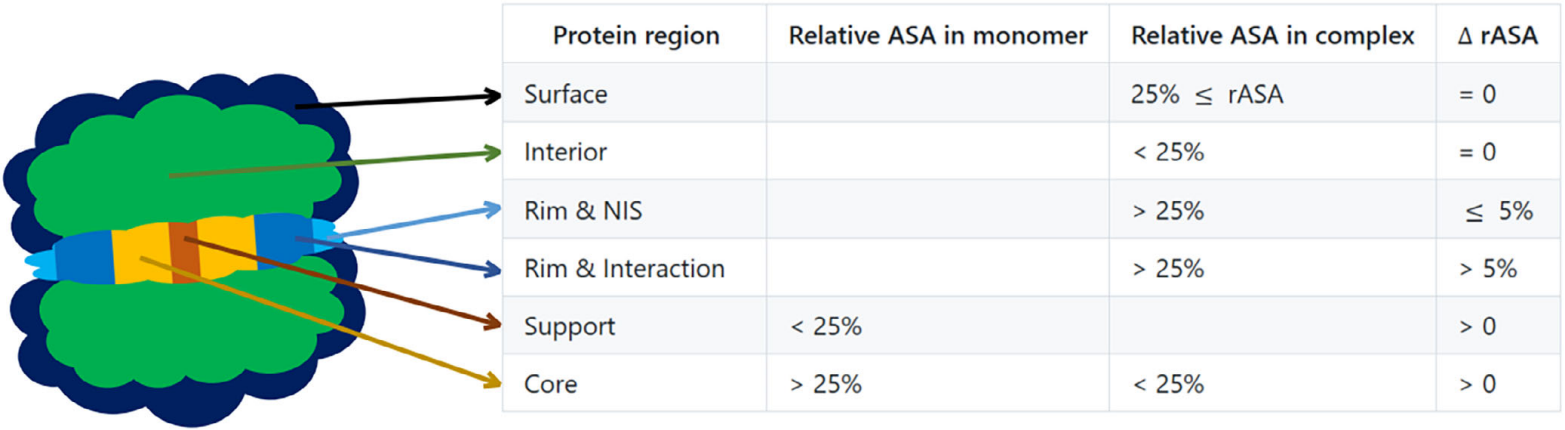
Parameters used to identify distinct protein regions based on residue ASA, along with a schematic representation of these regions.

We used the same relative accessible surface area (rASA)‐based method described by Levy (2010) to distinguish protein regions. Briefly, any residue was classified as part of the interface if its ΔrASA>0%. Rim residues were those with rASA>25% both before and after complex formation, support residues were those with rASA<25% already before complex formation, and core residues were those that shifted from rASA>25% to rASA<25% upon interface formation (Figure 8). Moreover, we distinguished residues on the rim of the interface as part of the interacting surface if their ΔrASA>5%, or as part of the non‐interacting surface (NIS) if their ΔrASA≤5% (Kastritis et al., 2014) (see Figure 8). For each residue, structural regions were determined based on theoretical and empirical maximum ASA values (*MaxASA*) (Hubbard, 1996; Lins et al., 2003; Miller et al., 1987; Rose et al., 1985; Samanta et al., 2002; Tien et al., 2013). The most frequently assigned region was then selected as the final location for the residue.

(1) $$\text{rASA}=\frac{\mathrm{ASA}}{\text{MaxASA}}$$

(2) $$\Delta \text{rASA}={\text{rASA}}_{\text{in}\;\text{monomer}}-{\text{rASA}}_{\text{in}\;\text{complex}}$$

Free‐solution monomers were approximated using monomer structures extracted from the complexes (listed in Section L in Supporting Information).

### 4.5 Characterization of pair diversity and key residue pairs in the dataset

To assess the diversity of residue pair classes in our dataset, we calculated the Shannon entropy $\left(H\right)$ for each protein region as

(3) $$H_{\text{pair}\;\text{in}\;\text{region}}=\begin{array}{l} -{\text{Frequency}}_{\text{pair}\;\text{in}\;\text{region}}\\ \times \log_{2}\left({\text{Frequency}}_{\text{pair}\;\text{in}\;\text{region}}\right) \end{array}$$

(4) $$H_{\text{region}}=\sum H_{\text{pair}\;\text{in}\;\text{region}}$$

The evenness index J (Pielou, 1966), reflecting the uniformity of pair class frequencies, was then computed as

(5) $$J=\frac{H_{\text{region}}}{H_{\max}}$$

where $H_{\max}$ is the maximum possible entropy (see Supporting Information).

To identify pairs of residue classes that are more likely to form an interacting pair than expected in the simplest case of completely random associations, we computed the Pointwise Mutual Information (PMI) score of an interacting pair class in a region as follows:

(6) $${\mathrm{PMI}}_{\text{pair}}\left\{\begin{array}{ll} \log_{2}\left(\frac{{\text{Frequency}}_{\text{pair}}}{{\text{Frequency}}_{\text{residue}\;1}\times {\text{Frequency}}_{\text{residue}\;2}}\right) & \text{if}\;\text{homogeneous}\;\text{pair}\\ \log_{2}\left(\frac{{\text{Frequency}}_{\text{pair}}}{2\times {\text{Frequency}}_{\text{residue}\;1}\times {\text{Frequency}}_{\text{residue}\;2}}\right) & \text{if}\;\text{heterogeneous}\;\text{pair} \end{array}\right.$$

The PMI was calculated using mean pair‐class and residue‐class frequencies sampled from the respective regions of stable and transient complexes. Values for the interior were derived from structures in the whole dataset.

## AUTHOR CONTRIBUTIONS

**Tom Miclot:** Conceptualization; writing – review and editing; data curation; formal analysis; software. **Stepan Timr:** Conceptualization; writing – review and editing; funding acquisition; supervision.

## FUNDING INFORMATION

Czech Academy of Sciences, Lumina Quaeruntur Fellowship LQ200402301.

## CONFLICT OF INTEREST STATEMENT

The authors declare no conflict of interest.

## Supporting information


**Data S1.** Supporting Information.

## Data Availability

The data that support the findings of this study are available from the corresponding author upon reasonable request.
