# Supplementary material for: Beyond contacts: The important role of the support region in protein complex assembly
Source: Protein Sci. 2026 Jan 20;35(2):e70470. doi: 10.1002/pro.70470 (PMC12817489; doi:10.1002/pro.70470)
Supplement: Supplementary file 1 — Data S1. Supporting Information. [file PRO-35-e70470-s001.pdf]

Supplementary information – Beyond contacts: the important role of the support region in protein complex assembly

Tom Miclot[1,*] and Stepan Timr[1,*]

[1]J. Heyrovsky Institute of Physical Chemistry, Czech Academy of Sciences, Prague, Czech Republic

[*]tom.miclot@jh-inst.cas.cz, stepan.timr@jh-inst.cas.cz

Contents

A Evaluation of dataset structure quality **S3**
A.1 Identity of proteins in the dataset S3
A.2 Quality of solved structures S4
A.3 Range of interface sizes S4

B Correction of protein complex structures **S6**
B.1 Adding missing atoms and optimizing hydrogen bonds S6
B.2 Optimizing hydrogen atom positions S6
B.3 Fixing missing bonds S6

C Comparison between stable and transient interfaces **S7**
C.1 Correlation between interface area and binding affinity S7
C.2 Overview of interfaces areas S8
C.3 Partitioning of interface area into the core, support, and rim regions S9

D Global residue composition of stable and transient interfaces **S11**
D.1 Amino acid composition of stable and transient interfaces S11
D.2 Composition of interface regions by residue classes S11

E Description and selection of residue pairs **S17**

F Residue pair interface composition **S18**
F.1 Relation between interface size and interaction or pair counts S18
F.2 Composition of residue pairs and their interactions in protein interfaces S19
F.3 Relation between pair counts and number of residues in interface region S20

G Evaluation of regions based on residue and residue pair counts **S21**

H Interaction type properties **S22**
H.1 Distribution of *van der Waals* contacts S22
H.2 Comparison of interaction counts in protein interiors and interfaces S22
H.3 Non-bonded interaction trends in protein regions S23
H.4 Evaluation of salt-bridge formation frequency by protein region S26

I Evaluation of pair diversity **S26**
I.1 Methods . . . S26
I.2 Results . . . S27

J Identification of key amino acid pairs **S28**

K Amino acid stickiness **S30**

L List of used structures **S32**

References **S57**

A Evaluation of dataset structure quality

Note Information concerning each protein complex comes from entries in the RCSB PDB database [1].

A.1 Identity of proteins in the dataset

More than half of the structures selected for the dataset are dimeric complexes from the PDBbind database and more than a quarter come from PPI4DOC (see Figure S1). Moreover, we included structures from BBM5.5, while other databases played a much smaller role in the composition of the dataset. However, the most important thing is not the database of origin but rather the species from which the structure is derived. *Homo sapiens* is the predominant species, accounting for 57.3 % of the dataset, followed by model organisms such as *Mus musculus*, *Escherichia coli*, *Saccharomyces cerevisiae* and multiple *Rattus* species. 9 other organisms, including a virus (HIV), each contribute more than 1 % of the dataset, with the remaining 27.8 % comprising other species. It is interesting to note that 2.4 % of the dataset correspond to synthetic constructs. Overall, despite the diversity, the dataset primarily represents human protein complexes.

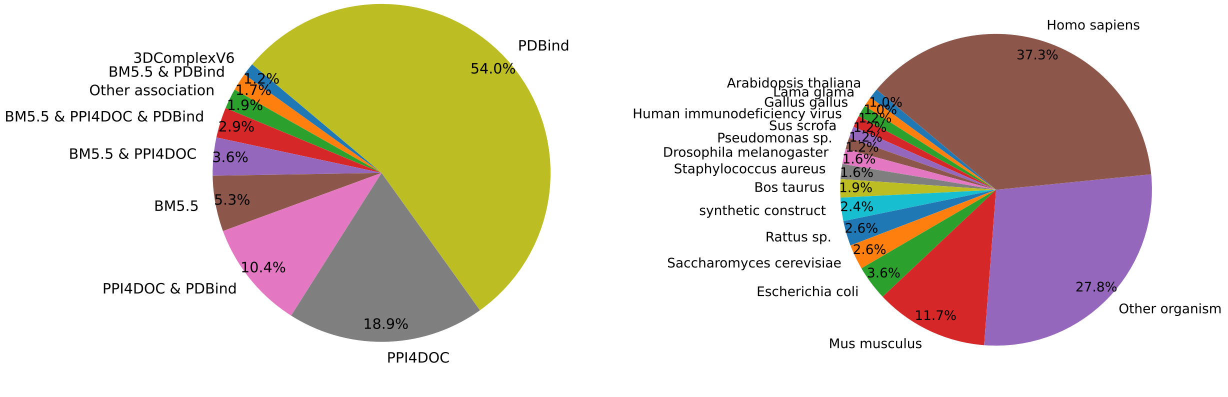

(a) (b)

Figure S1: (a) Partitioning of selected structures among databases. Note that we only retained dimeric structures from PDBbind; therefore, the quality analysis reflects this selection rather than the entire database. (b) Biological origin of the selected structures. Organisms classified as "other" each represent less than 1% of the dataset's diversity.

In the RCSB PDB database, the mutation information attribute indicates the presence of sequence mutations but does not provide further details. In general, these mutations are introduced through mutagenesis strategies used by authors to ensure crystallization of the target protein [2, 3]. Alternatively, mutations may be introduced to inhibit enzyme activity. As shown in Figure S2, a significant part of the dataset corresponds to enzymes, particularly hydrolases. In this regard, protein classification shows a broad range of categories in the dataset. The largest category is the *immune system*, followed by hydrolase-involving complexes (*hydrolase* and *hydrolase/hydrolase inhibitor*), and then *protein binding*. 11 additional categories each contribute more than 1% to the dataset, while 45.5% belong to other classifications. As a consequence, the dataset is not biased toward any single type of protein complex but displays sufficient diversity to serve as a general representation.

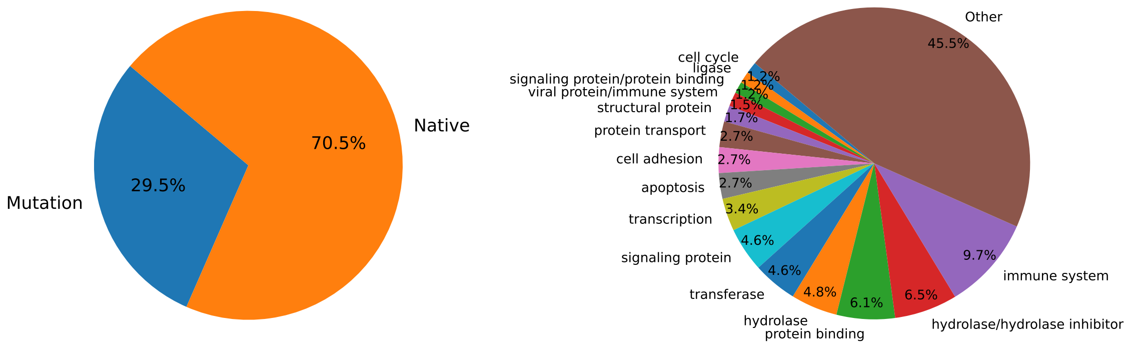

(a) (b)

Figure S2: (a) Proportion of mutated structures.(b) Classification of proteins constituting the dataset. Classification categories labeled as "other" each represent less than 1% of the dataset's diversity.

A.2 Quality of solved structures

Figure S3 shows that most protein complex structures were solved by X-ray diffraction, with resolutions predominantly between 1.80 to 2.50 Å. Structures with higher resolutions are less represented. This indicates a generally good level of structural quality; however, hydrogen atom positions typically need to be remodeled.

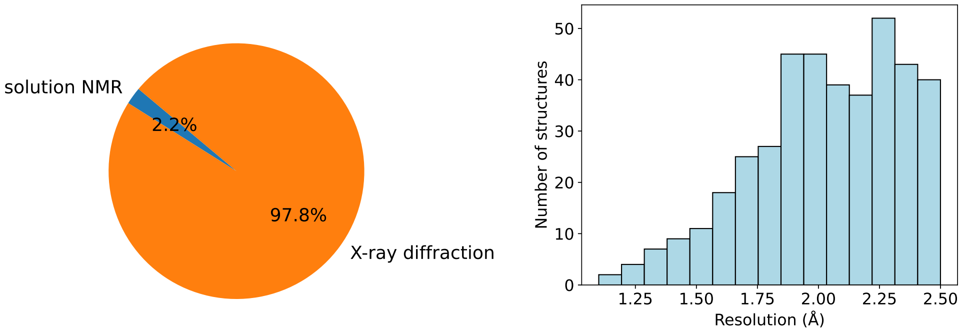

(a) (b)

Figure S3: (a) Proportion of methods used to solve the structures. (b) Resolution distribution of structures solved by X-ray diffraction.

A.3 Range of interface sizes

Method The accessible surface area (ASA) of both associated and free monomers forming the interface was calculated using the Shrake and Rupley algorithm [4] with the Golden Section Spiral algorithm implemented in MDTraj [5]. All atoms, including hydrogens, of protein residues were included in the calculation. The interface area was defined as half of the buried surface area (BSA), and the relative interface area was computed as the interface area divided by the ASA of each complex partner. The equations are provided below:

$$\mathrm{BSA} = (\mathrm{ASA}_{\mathrm{partner\ i}} + \mathrm{ASA}_{\mathrm{partner\ j}}) - \mathrm{ASA}_{\mathrm{complex}} \tag{S1}$$

$$\text{interface area} = \frac{\mathrm{BSA}}{2} \tag{S2}$$

$$\text{relative interface area} = \frac{\text{interface area}}{\text{ASA}_{\text{partner i or j}}} \times 100 \tag{S3}$$

Results The interface area distribution of the dataset peaks at around 1000 Å^2, which is in line with the reported average of 1227 Å^2 [6]. However, the distribution profile below 1000 Å^2 is different, showing relatively reduced information in this range. It is interesting to note that, despite the wide variation in interface areas, they account for only around 5–15% of the total surface area of each protein partner forming a complex.

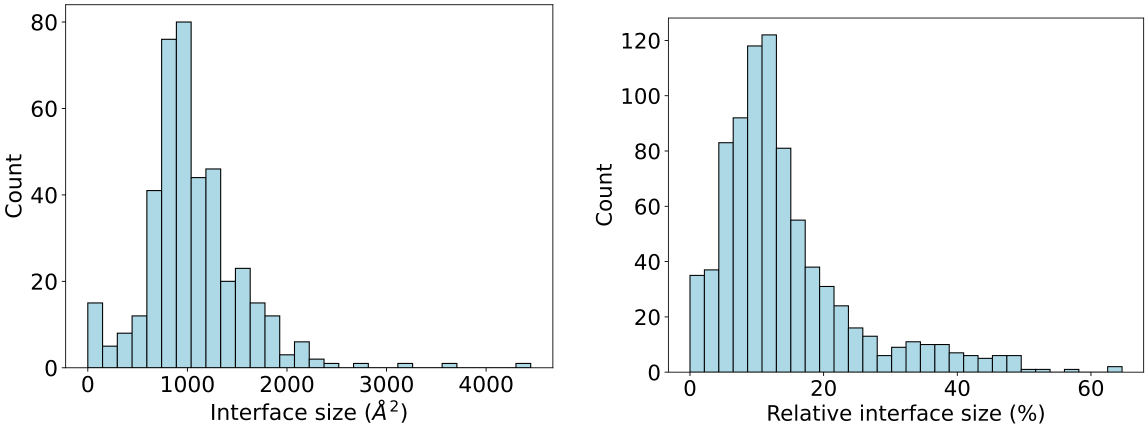

(a) (b)

Figure S4: Distributions of (a) interface areas and (b) relative interface areas in the dataset.

B Correction of protein complex structures

B.1 Adding missing atoms and optimizing hydrogen bonds

All heteroatoms, as well as unknown residues (UNK), were removed from the original file. PDB2PQR [7] was then used with its PROPKA [8, 9] parsing option to assign the protonation states of residues in the structure and add missing hydrogen atoms. A pH of 7.2 was used when the value was unspecified. An additional option was employed to ensure that rebuilt atoms did not overlap or lie too close to one another. The procedure also included optimization of hydrogen bonds by the software. The output from PDB2PQR was exported in PDB file format and submitted to PDBFixer [10] to rebuild missing heavy atoms and ensure that atom names matched those of the AMBER ff14SB force field [11]. The same pH value was used as that provided to PDB2PQR.

B.2 Optimizing hydrogen atom positions

The previous steps reconstructed hydrogen atoms in non-optimized positions, except for those making hydrogen bonds. To eliminate clashes or bad positions of hydrogen atoms, a vacuum minimization was performed using OpenMM [10]. Constraints were imposed on hydrogen bonds to avoid the loss of previously optimized hydrogen bonds, except in NMR-derived structures. In addition, position restraints of 1.0e5 kJ/(mol nm$\hat{2}$). were applied to heavy atoms to prevent displacement from their initial positions. This ensured that the overall structure of the complex did not change compared to the experimental structure and avoided force field-dependent modifications. No limit was placed on the number of minimization steps, allowing the minimization to continue until convergence.

B.3 Fixing missing bonds

In some cases, bonded atoms may not be correctly identified by software such as OpenMM or MDTraj [5]. To address this problem, bonded atoms were recognized using the same method as implemented in VMD [12]: two atoms were considered bonded if their distance d satisfied $d_{min} \leq d \leq d_{max}$, where:

$$d_{max} = 0.6 \times (radius_{vdw\ atom\ 1} + radius_{vdw\ atom\ 2}) \tag{S4}$$

$$d_{min} = radius_{covalent\ atom\ 1} + radius_{covalent\ atom\ 2} \tag{S5}$$

Van der Waals and covalent radii of atoms were taken from *CRC Handbook of Chemistry and Physics* [13]. The CONNECT records of the PDB file were then generated for all bonded atoms identified.

Disulfide and diselenide bridges were then identified based on the distance between their chalcogen atoms (sulfur or selenium). Cysteines involved in a bridge were renamed as CYX, while selenocysteines involved in a diselenide bridge were renamed as XSE. Finally, CONNECT records were written for the bonded S/Se atom pairs.

C Comparison between stable and transient interfaces

C.1 Correlation between interface area and binding affinity

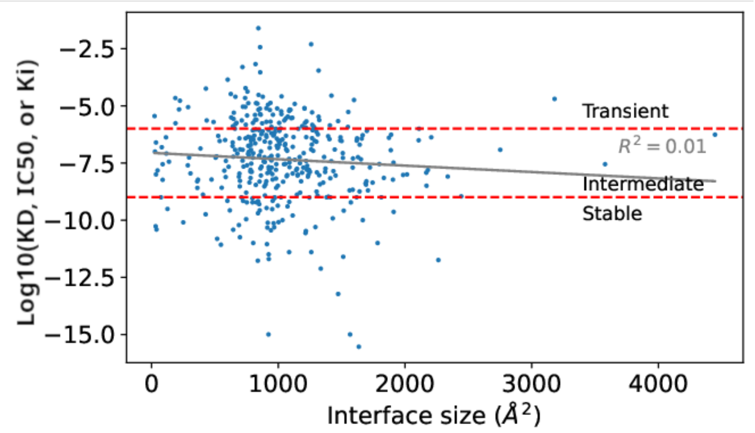

Figure S5: There is no significant correlation between interface area and binding affinity in our dataset.

C.2 Overview of interfaces areas

Stable and transient complexes exhibit a highly similar distribution of interface areas in our dataset, and the distribution of relative interface areas also shows a marked resemblance. The main difference lies in the number of structures, which reflects a size difference between the two data subsets. This suggests that analyzing the entire interface may not be a highly discriminative approach, as the overall characteristics of stable and transient complexes appear to be comparable. To gain a deeper understanding of the underlying differences, we adopted a more nuanced approach by dividing the interface into distinct regions—the core, support, and rim—as described by [14]. Examining these regions individually allowed us to uncover more subtle distinctions that may not be apparent when analyzing the interface as a whole.

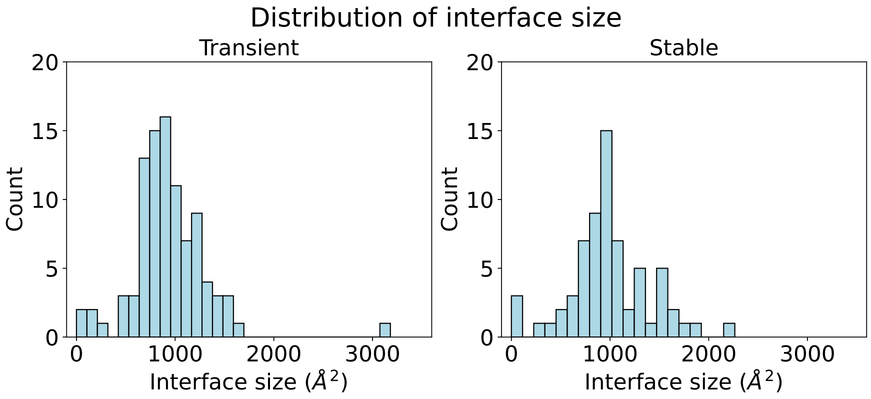

(a)

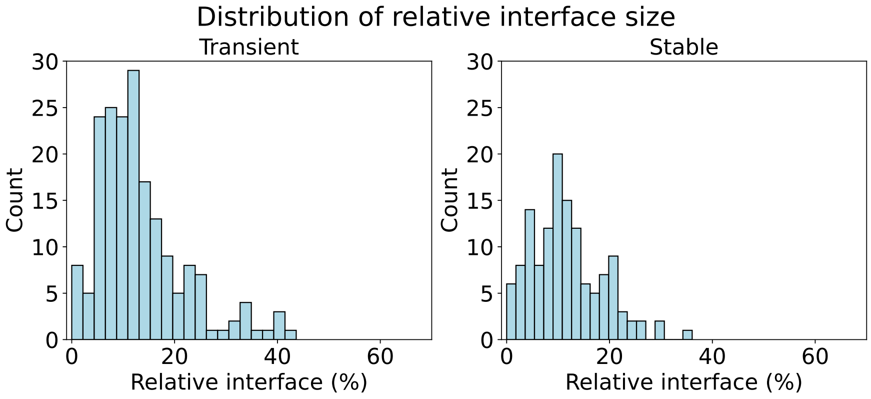

(b)

Figure S6: Distributions of (a) interface areas and (b) relative interface areas show minimal differences between stable and transient complexes in the dataset.

C.3 Partitioning of interface area into the core, support, and rim regions

	Total interface (Å^2)	Interface core (Å^2)	Interface support (Å^2)	Interface rim (Å^2)	Ratio core (%)	Ratio support (%)	Ratio rim (%)
**Stable**							
mean	989.874677	607.208394	123.476492	259.189791	58.580564	11.501436	29.918000
std	425.296214	275.951603	72.097827	125.723235	13.236188	4.769504	15.900780
25%	796.521692	461.666735	81.986300	178.704510	53.615981	9.168470	19.743660
50%	934.196376	579.348745	102.934854	241.232073	60.788616	11.522166	27.612149
75%	1217.829464	774.178134	170.973008	299.066617	67.002718	15.455441	33.495636
**Intermediate**							
mean	1092.089007	664.355783	106.509581	321.223643	58.438802	10.008344	31.552854
std	545.701572	354.982146	62.237815	178.345136	13.036700	6.265982	14.088080
25%	784.718914	451.388460	64.511889	219.188447	54.443073	7.212961	24.083959
50%	995.119005	638.520167	99.387733	283.506684	61.538548	9.349579	28.718762
75%	1326.425056	838.759044	145.916346	395.864791	66.131390	12.158629	35.452407
**Transient**							
mean	933.722276	554.794663	90.295209	288.632405	56.450750	10.029828	33.519422
std	400.471166	293.255516	60.658658	125.472156	14.281923	7.388917	14.577434
25%	740.403233	419.622314	56.147998	208.572851	53.272626	6.336329	26.268331
50%	878.268117	522.612732	82.317239	270.496404	58.892119	8.388054	31.737366
75%	1111.908862	651.509193	115.613281	356.622329	65.125049	12.776661	36.997802

Table S1: Descriptive statistics of interface region areas and their proportions within the total interface.

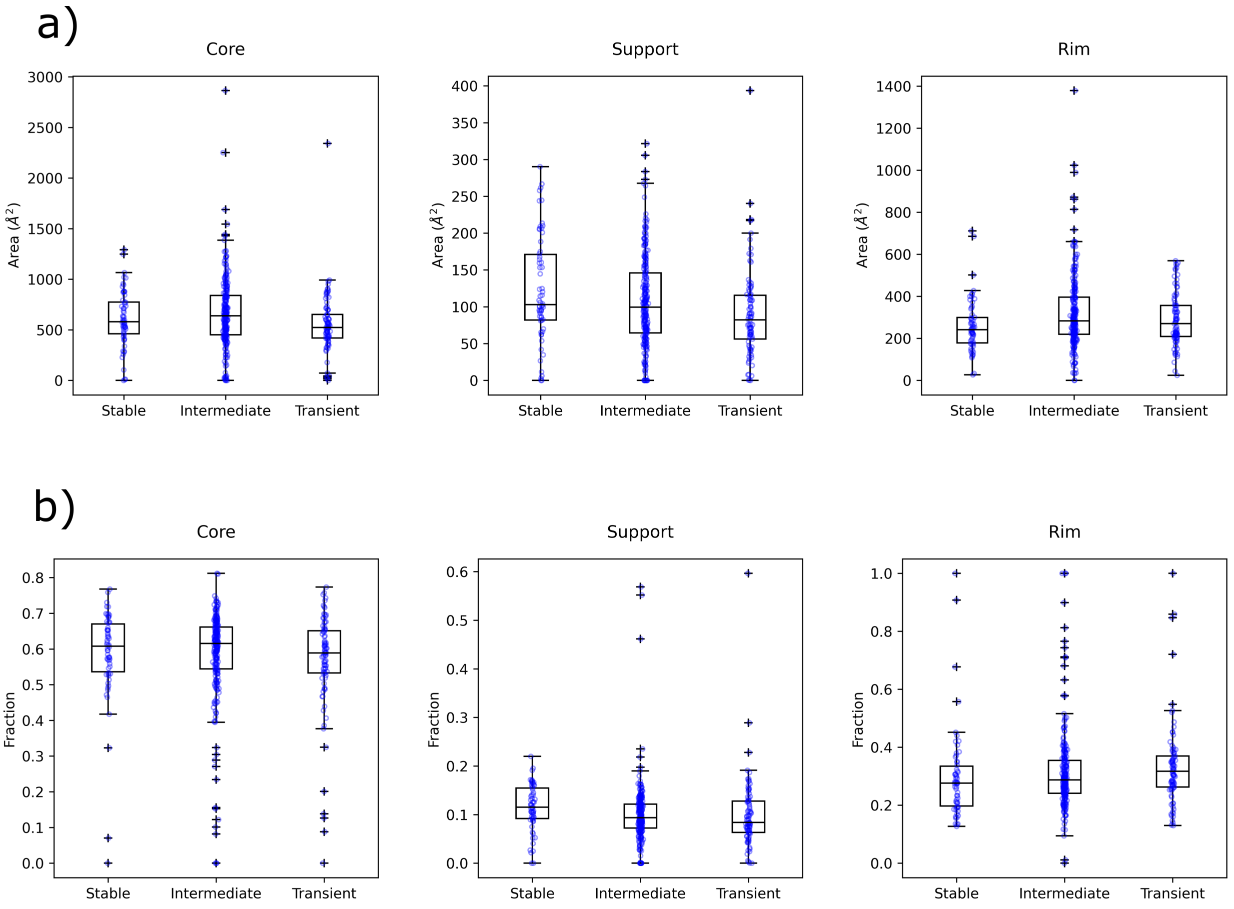

Figure S7: Box plots showing (a) the areas of the distinct interface regions and (b) their relative fractions. Each box represents the interquartile range (25th to 75th percentile) with the median marked by a horizontal line. Whiskers extend to 1.5 times the interquartile range, with outliers shown as crosses. Individual data points are overlaid as open circles. An overall significant difference among groups was detected via the Kruskal–Wallis test ($P < 0.05$) for all the regions in the areas, and for the support and the rim in the relative fractions. Pairwise comparisons conducted using Wilcoxon rank-sum tests with Bonferroni correction indicated that specific differences were significant ($P < 0.05$) between the areas of $\mathrm{core}_{intermediate}$ and $\mathrm{core}_{transient}$, $\mathrm{support}_{stable}$ and $\mathrm{support}_{transient}$, and $\mathrm{rim}_{intermediate}$ and $\mathrm{rim}_{transient}$, as well as between the relative fractions of $\mathrm{support}_{stable}$ and $\mathrm{support}_{intermediate}$, $\mathrm{support}_{stable}$ and $\mathrm{support}_{transient}$, and rim_{stable} and $\mathrm{rim}_{transient}$. The effect sizes were quantified using the probability of superiority, with their 95% confidence intervals estimated using bootstrapping.

D Global residue composition of stable and transient interfaces

D.1 Amino acid composition of stable and transient interfaces

Analysis of residue frequencies in stable and transient interfaces reveals similar compositions for most residues, but substantial variation for a few, such as leucine. However, it is the physico-chemical properties of the residues that primarily influence interface stability. Therefore, the following analyses focus on these properties to better understand their role in complex formation.

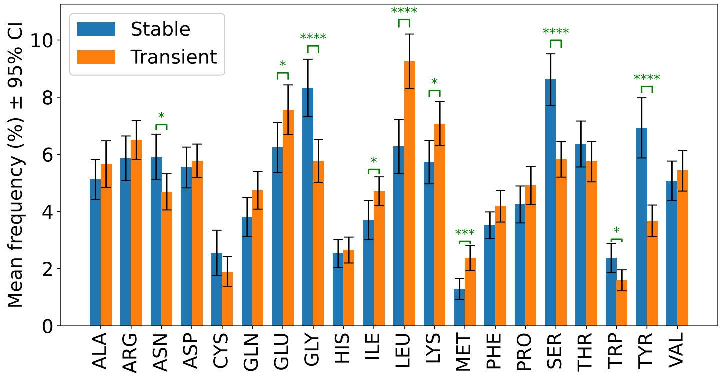

Figure S8: Mean residue frequency of each residue populating stable and transient interface. The standard error of the mean with an appropriate Students t coefficient for a 95% confidence level was used to estimate uncertainty. Significance of differences between stable and transient complexes was tested using Welch's t-test. Statistical significance is denoted as follows: $P < 0.05$ (*), $P < 0.01$ (**), $P < 0.001$ (***), $P < 0.0001$ (****). 95% confidence intervals for the mean differences reported in the text were obtained using Welch's t-test.

D.2 Composition of interface regions by residue classes

The distribution of residues in the interface is evaluated based on their physico-chemical classes, as defined by Pommié et al. [15], rather than by individual residue identities, as previously considered.

Charge and polarity are key parameters used to understand protein complex formation, describe relevant interfaces, and predict binding affinity [16, 17, 18]. Our analysis investigates the distribution of residues classes across the different interface regions. We find no significant difference in charge distribution between stable and transient interfaces in the core and support regions. However, a slight variation is observed in the rim, where transient complexes exhibit a rim (interaction) with a higher proportion of both positive and negative charges, whereas their rim is more positively charged. This finding is consistent with previous work by Grassmann et al. [19]. In contrast, polarity remains unchanged in the rim but exhibits high variation in the core and support regions, where stable interfaces display higher polarity. This can be partially explained by the classification of tyrosine as a polar residue by Pommié et al. [15], due to its hydroxyl group (-OH).

Furthermore, examination of other residue properties, such as hydropathy, reveals no significant differences in the rim, but notable variations in other regions. Analysis of potential hydrogen bonding partners shows no significant variation in the rim; however, the core and support regions of transient interfaces contain a higher proportion of residues with side chains that can neither act as a hydrogen bond acceptor nor as a donor. This suggests that transient interfaces have a reduced capacity to form hydrogen bonds than stable ones. Chemical class distributions exhibit more variation across interface regions. In particular, aromatic residues are less represented in transient interfaces. The rim region of transient complexes contains fewer hydroxyl residues but more acidic and basic residues compared to stable interfaces. In the support and core regions, the primary variations are observed for aliphatic and aromatic residues, with stable interfaces showing higher

aromaticity and lower aliphatic content. Additionally, amide residues are slightly underrepresented in the core region of transient interfaces.

Mean proportions (i.e., frequency) of residues are shown in figures below for each physico-chemical class. The standard error of the mean with an appropriate Students t coefficient for a 95% confidence level was used to estimate uncertainty. Significance of differences between stable and transient complexes was assessed using Welch's t-test. Statistical significance is denoted as follows: $P < 0.05$ (*), $P < 0.01$ (**), $P < 0.001$ (***), $P < 0.0001$ (****). Significance of differences between regions—reported in the text—was assessed using paired t-test. 95% confidence intervals for the mean differences reported in the text were obtained using the t-tests.

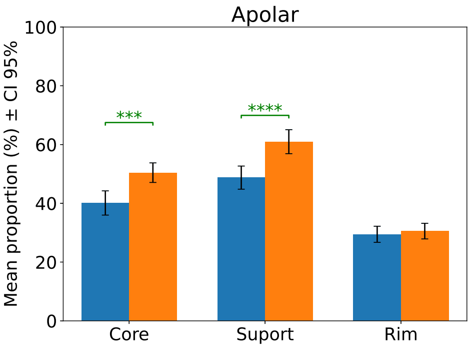

(a)

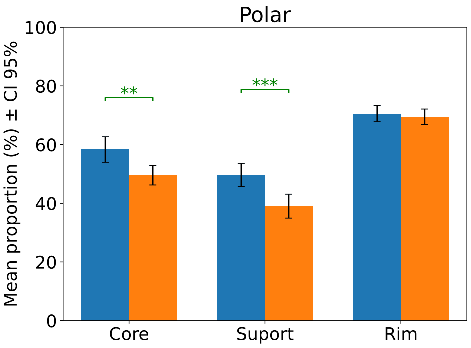

(b)

Figure S9: Class: Polarity. Mean frequency of residues in different interface regions. Blue: Stable complexes. Orange: Transient complexes.

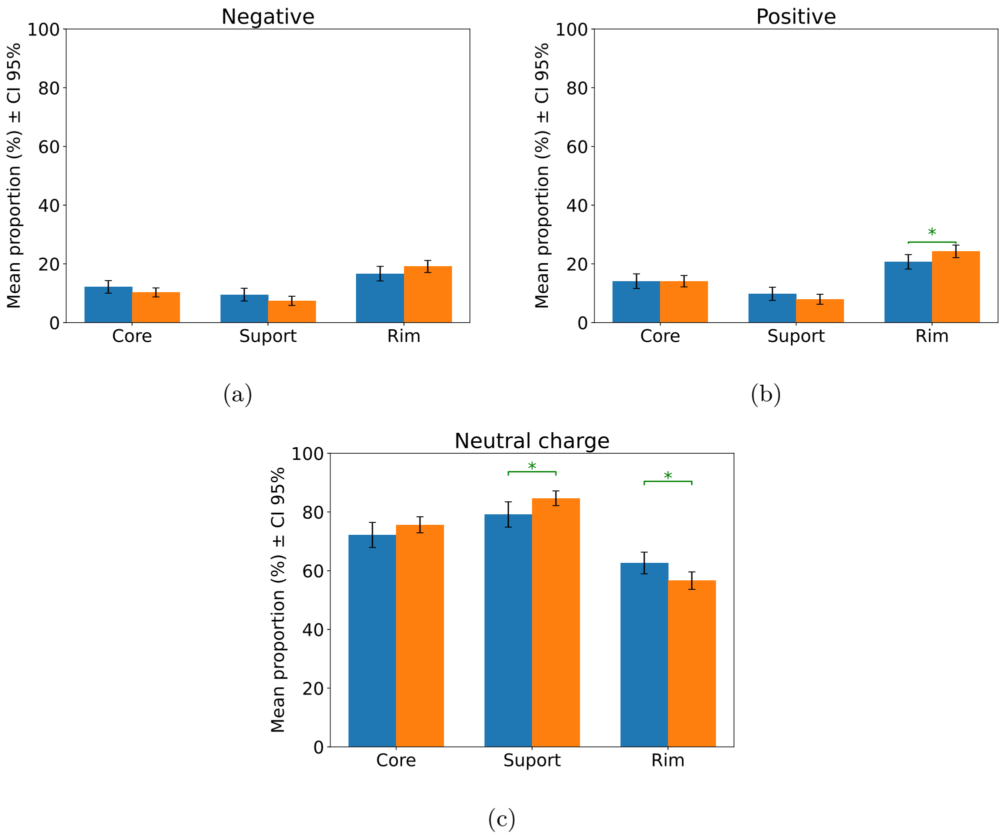

Figure S10: Class: Charges. Mean frequency of residues in different interface regions. Blue: Stable complexes. Orange: Transient complexes.

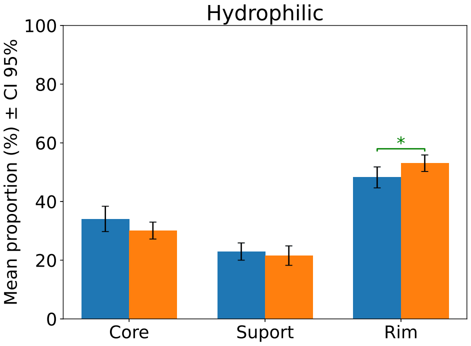

(a)

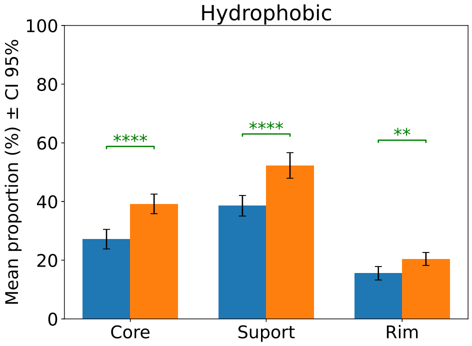

(b)

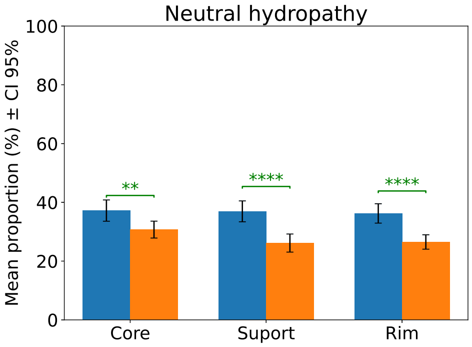

(c)

Figure S11: Class: Hydropathy. Mean frequency of residues in different interface regions. Blue: Stable complexes. Orange: Transient complexes.

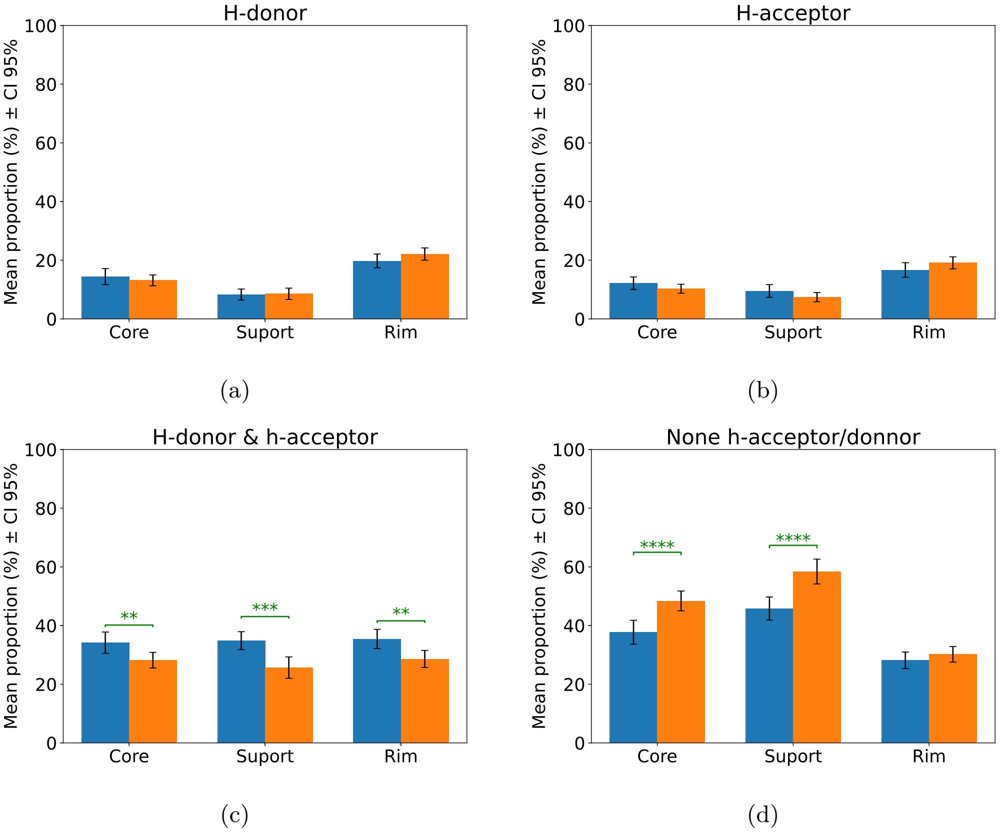

Figure S12: Class: H-bond. Mean frequency of residues in different interface regions. Blue: Stable complexes. Orange: Transient complexes.

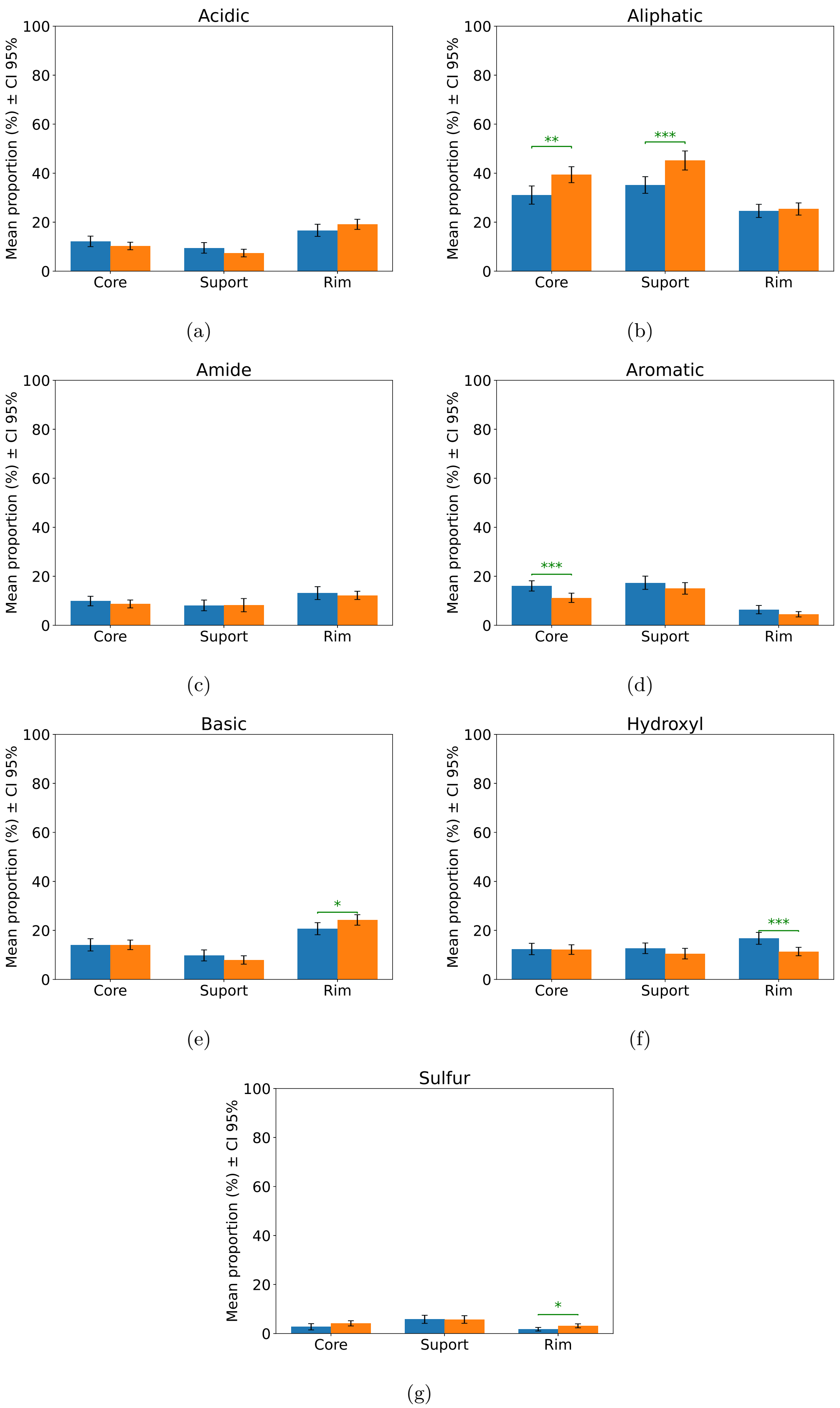

Figure S13: Class: Chemical class. Mean frequency of residues in different interface regions. Blue: Stable complexes. Orange: Transient complexes.

E Description and selection of residue pairs

We considered two residues to form a residue pair only if we identified an interaction between them. Describing pairs based solely on residue names and secondary structures can be overly complex and insufficiently detailed with respect to side-chain and backbone interactions. To ensure a simplified yet more comprehensive representation, each residue was modeled using two beads: one for the side chain and one for the backbone (BB). The side-chain bead was named after the residue itself; for example: the side chain of an arginine residue was labeled ARG. In contrast, the backbone bead was labeled according to the secondary structure (Helix: H, Strand:E, Coil: C), rather than the residue name, as the backbone conformation reflects the secondary structure in which the residue is located. Thus, the nomenclature included 23 beads: 20 for side chains and 3 for backbone types. Pairs containing identical beads are considered equivalent; for instance, ARG-HIS and HIS-ARG represent the same pair.

All consecutive residues (i,i+1) were excluded from the analysis. Finally, only pairs in which both residues belonged to the interior were considered part of the protein interior. Analogously, residue pairs were categorized according to their location in the core, support, and interacting rim of protein–protein interfaces, as well as in the NIS rim to study the extended area around the interface.

F Residue pair interface composition

Method The primary objective of this analysis is to provide a comprehensive overview of the composition of protein–protein interfaces in terms of their residue pair and interaction content. To achieve this, we do not distinguish between stable, transient, or intermediate complexes, nor do we differentiate among the various protein regions that contribute to the interface. Instead, we focus on the distributions of the number of interaction types (e.g., van der Waals, hydrogen bonds) and interactions. To illustrate these approaches, we can use van der Waals (vdW) interactions as an example: For a given interface, we record the presence or absence of a vdW interaction without considering their number. In contrast, interaction count refers to the actual number of vdW interactions between residue pairs forming the interface. These approaches are applied to all interaction types and each PDB structure. The same protocol is employed for residue pairs, utilizing a simplified two-bead representation (i.e., backbone and side chain). Finally, we analyze these values by plotting them against interface areas and by examining their distributions across the dataset, thereby providing insight into the major characteristics of the dataset.

F.1 Relation between interface size and interaction or pair counts

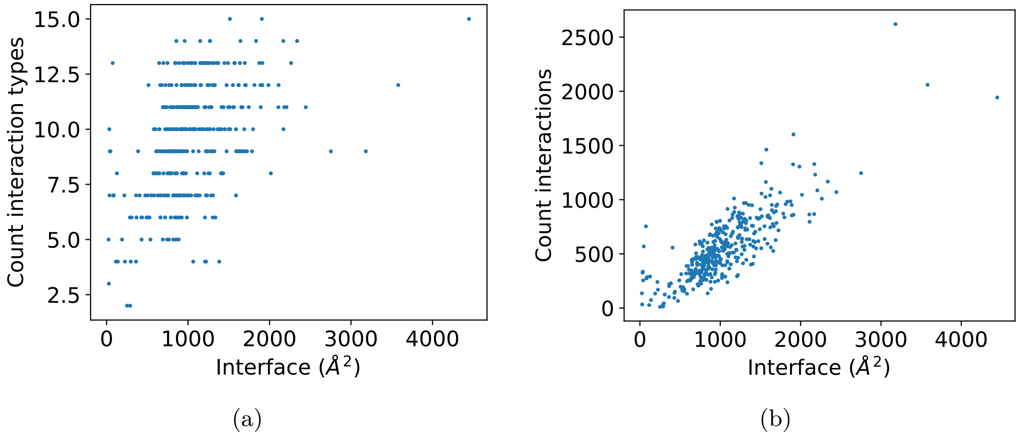

Figure S14: Correlation between interface size and (a) the number of interaction types or (b) the total number of interactions forming the interface.

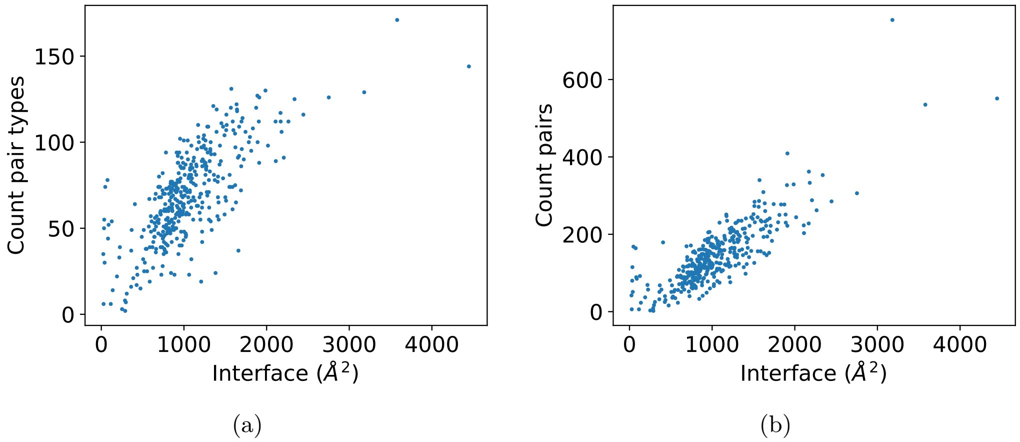

Figure S15: Correlation between interface size and (a) the number of pair types or (b) the number of interacting pairs.

F.2 Composition of residue pairs and their interactions in protein interfaces

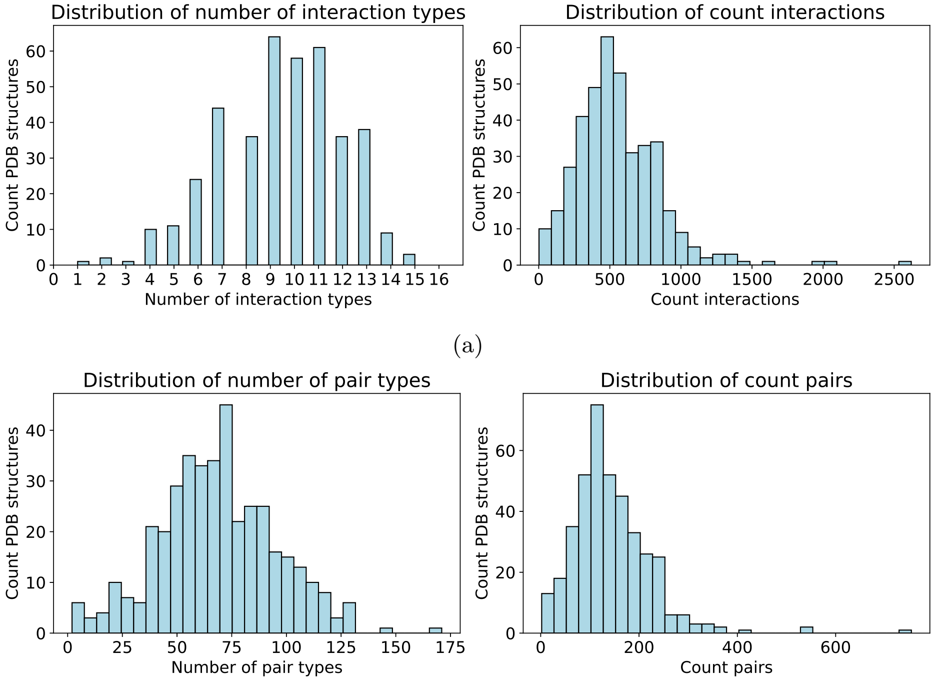

(a)

(b)

Figure S16: (a) Distribution of the number of interaction types and the number of interactions over the structure dataset. (b) Distribution of the number of pair types and the number of pairs over the structure dataset.

F.3 Relation between pair counts and number of residues in interface region

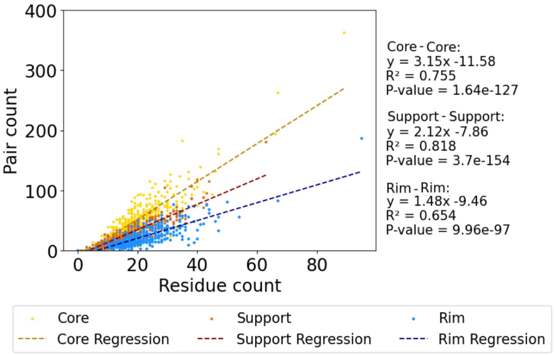

(a)

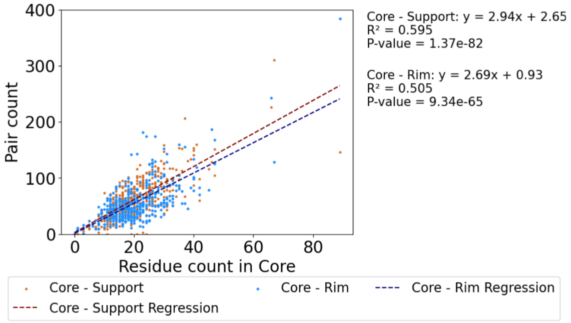

(b)

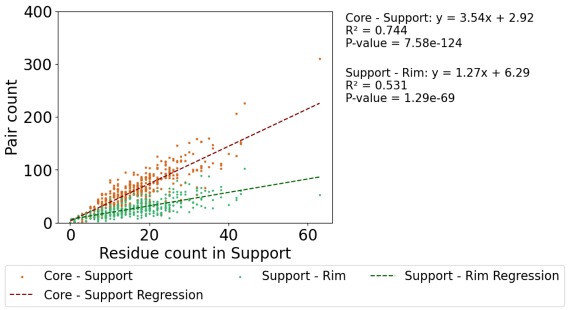

(c)

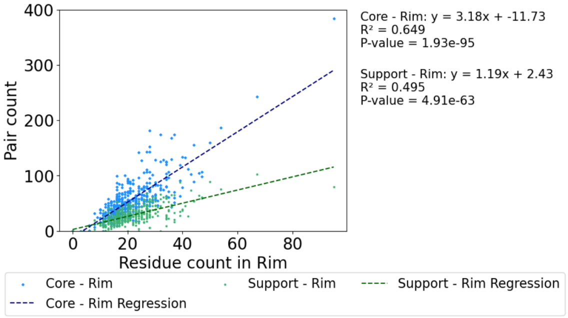

(d)

Figure S17: Number of interacting residue pairs within each region (a) and across the regions (b-d) as a function of the residue count in the given region. Note that the rim and rim (NIS) were combined for this analysis. The overall scaling is $\approx 3.2 + 3.0 + 2.7 = 8.9$ for the core, $\approx 2.1 + 3.5 + 1.3 = 6.9$ for the support, and $\approx 1.5 + 3.2 + 1.2 = 5.9$ for the rim. Therefore, although the numbers become more comparable, the hierarchy (core $>$ support $>$ rim) of the pair count per residue remains preserved when "cross-region" pairs are included.

G Evaluation of regions based on residue and residue pair counts

The Pandas *.describe* function was used to compute summary statistics for the number of residues or interacting residue pairs in each protein region. The average (mean), standard deviation (std), minimum (min) and maximum (max), as well as quartiles are displayed in the table below. Among all regions, the core contains the highest number of residues and pairs, while the rim NIS has the fewest.

	**Residues**				**Pairs**			
	Core	Rim NIS	Support	Rim	Core	Rim NIS	Support	Rim
Database								
mean	20.363	5.998	16.843	15.343	52.346	1.182	27.570	13.622
std	9.393	3.051	8.849	7.195	35.162	1.914	21.210	12.161
min	0.000	0.000	0.000	2.000	0.000	0.000	0.000	0.000
25%	14.000	4.000	11.000	11.000	30.250	0.000	13.000	6.000
50%	19.000	5.000	16.000	14.000	48.000	0.000	24.000	10.500
75%	25.000	8.000	22.000	18.000	67.000	2.000	38.000	18.000
max	89.000	20.000	63.000	75.000	363.000	11.000	181.000	119.000
Stable								
mean	19.905	5.698	20.063	13.937	45.635	1.333	34.651	9.603
std	7.558	3.420	9.296	5.535	24.711	2.222	24.255	8.202
min	3.000	0.000	2.000	6.000	2.000	0.000	0.000	0.000
25%	14.000	3.500	14.000	11.000	29.000	0.000	18.500	4.500
50%	18.000	5.000	18.000	13.000	43.000	0.000	28.000	6.000
75%	24.500	7.500	24.500	16.500	60.000	2.000	45.500	13.500
max	40.000	14.000	42.000	33.000	131.000	9.000	117.000	42.000
Intermediate								
mean	21.291	6.061	17.113	16.142	56.810	1.138	27.862	14.980
std	10.099	3.075	8.627	8.053	37.445	1.847	19.450	13.703
min	0.000	0.000	0.000	2.000	0.000	0.000	0.000	0.000
25%	15.000	4.000	11.000	11.000	35.000	0.000	13.500	7.000
50%	19.000	6.000	17.000	14.000	53.000	0.000	25.000	11.000
75%	26.000	8.000	22.000	19.000	71.000	2.000	39.000	19.000
max	89.000	20.000	44.000	75.000	363.000	11.000	115.000	119.000
Transient								
mean	18.185	6.033	13.913	14.163	44.957	1.196	21.935	12.728
std	8.179	2.723	8.300	5.234	33.183	1.882	22.176	9.018
min	1.000	1.000	1.000	3.000	0.000	0.000	0.000	0.000
25%	14.000	4.000	9.750	10.000	25.000	0.000	8.750	7.000
50%	18.000	6.000	13.000	13.000	38.500	0.000	18.500	10.500
75%	21.000	8.000	17.250	18.000	54.250	2.000	30.000	17.250
max	67.000	13.000	63.000	26.000	263.000	10.000	181.000	39.000

Table S2: Statistics of residue and residue pair counts found in each protein region.

H Interaction type properties

H.1 Distribution of *van der Waals* contacts

The number of van der Waals (vdW) contacts was counted between residues in all non-consecutive pairs, without applying any other selection criteria. The figure below shows that a typical vdW interaction between residues forming a pair involved fewer than five contacts.

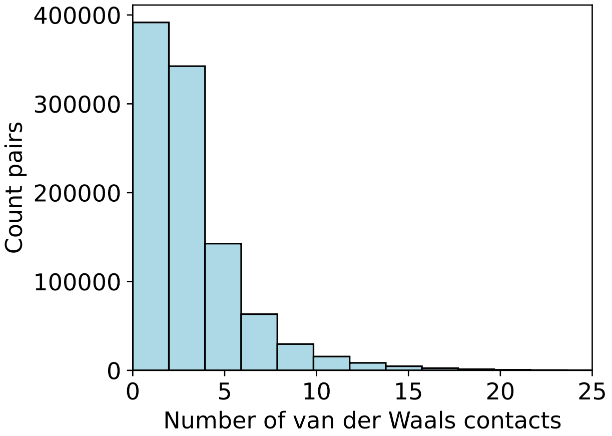

Figure S18: Distribution of number of van der Waals contact between non-consecutive residue pairs.

H.2 Comparison of interaction counts in protein interiors and interfaces

Results

**Interaction type**	**All**	**Interior**	**Core**	**Support**	**Rim (interaction)**	**Rim (NIS)**
Amino−Π	241	197	24	16	3	1
Aromatic−Anion	1380	1119	127	102	29	3
Aromatic−Aromatic	1990	1734	106	141	9	0
Aromatic−Cation	943	690	156	66	30	1
Aromatic−S/Se	2270	2074	104	86	6	0
C-bond	105	96	3	3	3	0
Charge clash	217	160	29	11	16	1
ARG−ARG stacking	112	70	19	15	7	1
Charge repulsion	5725	3872	774	227	768	84
Hydrogen bond	43435	39235	2379	1309	469	43
Hydrogen bond: H−Π	22565	19988	1644	832	101	0
Hydrophobic	21940	20684	688	544	24	0
Hydrophobic−Hydrophilic clash	6512	5466	644	304	90	8
Hydrophobic−Hydrophilic repulsion	59262	52821	3690	1618	1043	90
S/Se Chalcogen or Hydrogen bond	831	774	28	25	4	0
Salt bridge	1513	1015	244	68	182	4
$n \rightarrow \Pi^*$	38	34	4	0	0	0
van der Waals	1233848	1106633	71726	40144	14206	1139

Table S3: Total number of interactions by type across different regions in the entire dataset.

H.3 Non-bonded interaction trends in protein regions

We computed the average interaction count by any residue pair capable of forming that specific interaction and located in a given protein region, without considering the stability of the complexes. For binary interactions (i.e., present: 1, absent: 0), such as ARG–ARG stacking, the Wilson score interval [20] at the 95% confidence level was used to estimate the uncertainty in the average value. To assess whether differences in averages between two regions are statistically significant, the two-proportion z-test was applied. For other, non-binary interactions (e.g., van der Waals or hydrogen bonds), the standard error of the mean with an appropriate Students t coefficient for a 95% confidence level was used to estimate uncertainty. Significance of differences between regions was tested using Welch's t-test. Statistical significance is denoted as follows: $P < 0.05$ (*), $P < 0.01$ (**), $P < 0.001$ (***), $P < 0.0001$ (****). 95% confidence intervals for the effect sizes reported in the text were obtained using the Newcombe method for binary interactions and using Welch's t-test for non-binary interactions.

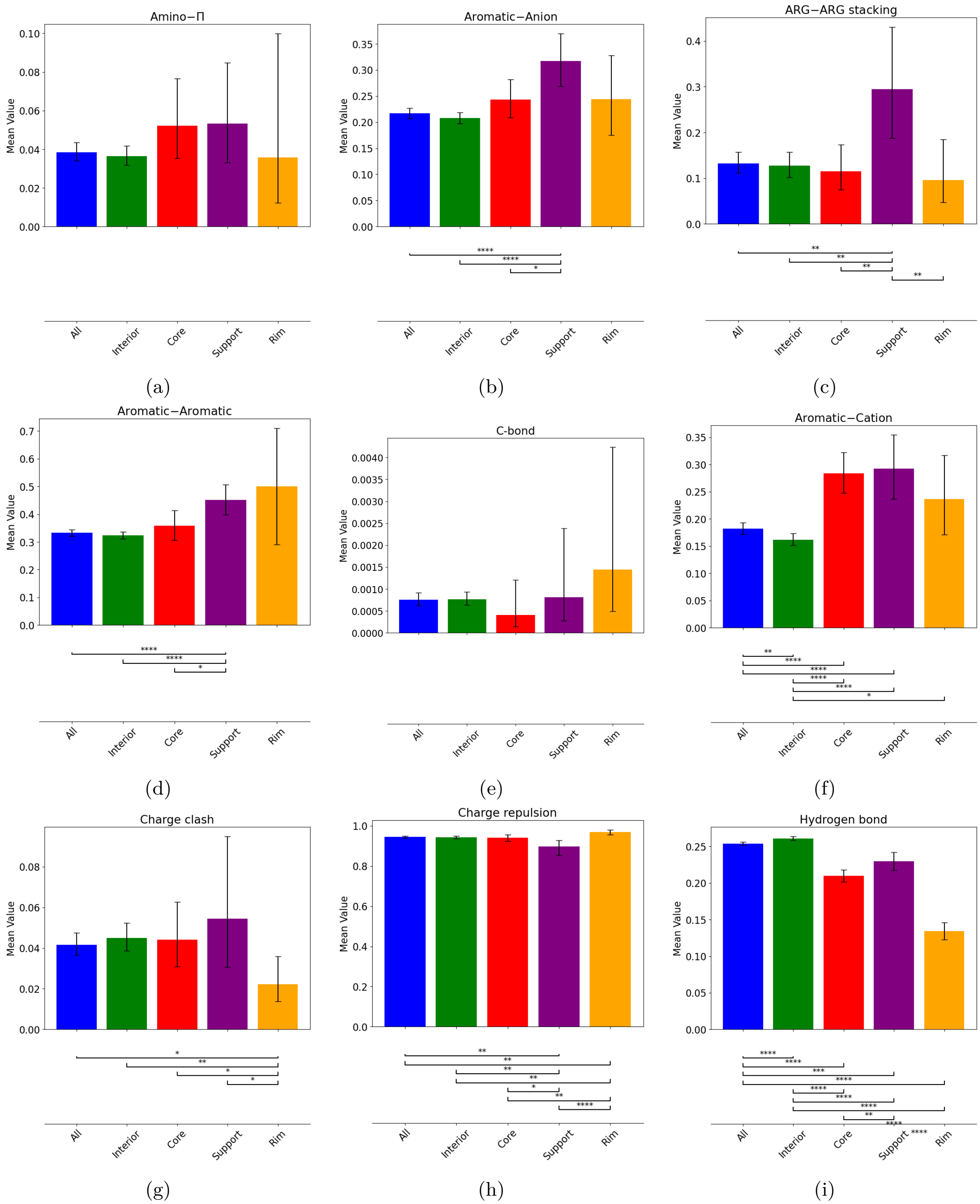

Figure S19: *The figure continues on the next page*

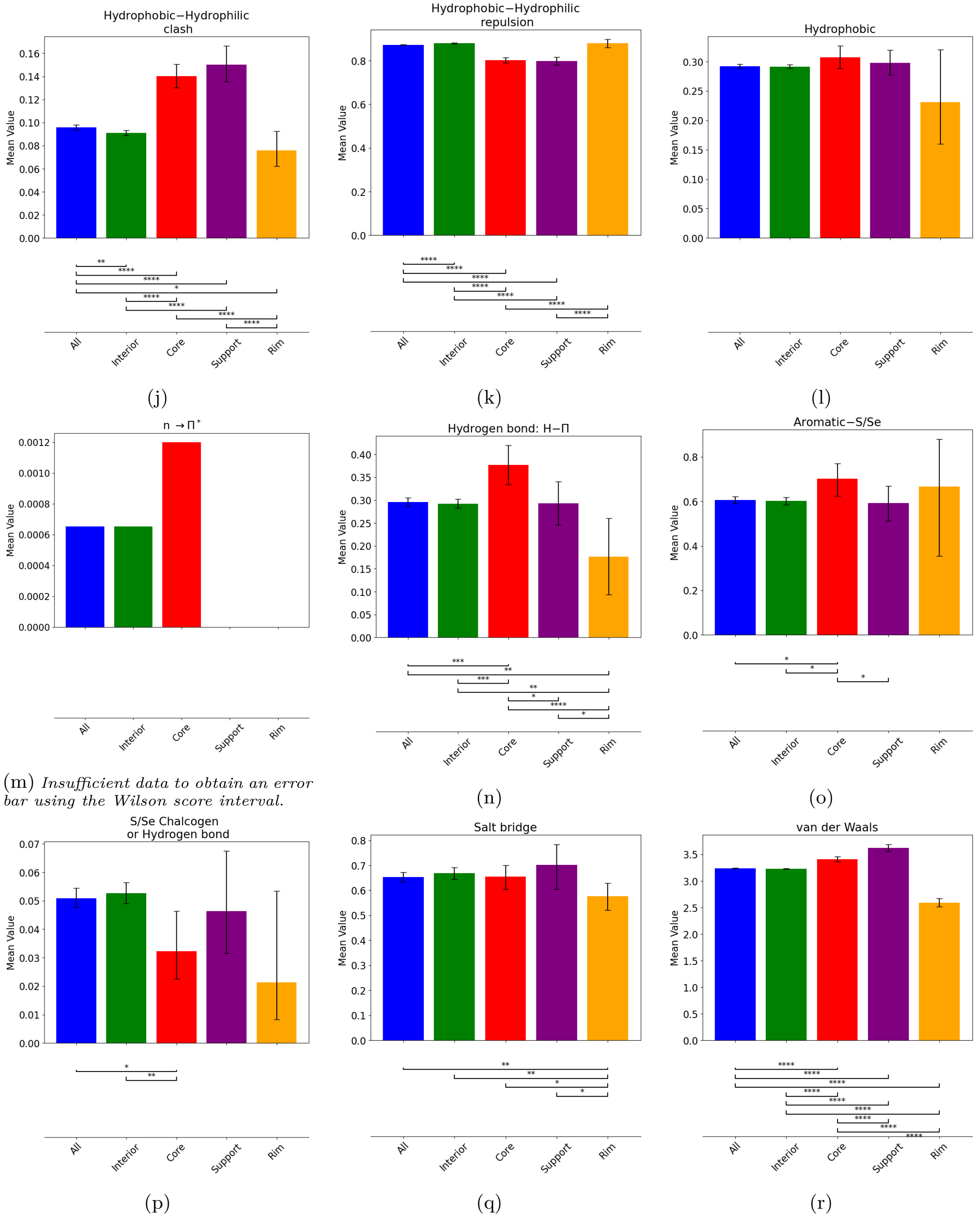

Figure S19: Non-bonded interaction trends in protein regions. Average interaction count by any residue pair capable of forming that specific interaction and located in a given protein region, without considering the stability of the complexes. The error bars and statistical significance of the differences were evaluated as explained in the previous paragraph.

H.4 Evaluation of salt-bridge formation frequency by protein region

ARG–ASP, ARG–GLU, ASP–LYS, and GLU–LYS are residue pairs capable of forming salt bridges. Their frequency of salt bridge formation is assessed for each protein region, irrespective of complex stability. Notably, within the interface, all these pairs exhibit a higher frequency of salt bridge formation in the support region.

Pair	All	Interior	Core	Support	Rim
ARG-ASP	0.73	0.75	0.72	0.77	0.61
ARG-GLU	0.70	0.73	0.67	0.71	0.62
ASP-LYS	0.52	0.45	0.65	0.67	0.61
GLU-LYS	0.56	0.60	0.53	0.60	0.47

Table S4: Frequency of residue pairs capable of forming salt bridges within the different interface regions. The rim NIS region is excluded from the analysis.

I Evaluation of pair diversity

I.1 Methods

This analysis aims to provide a comprehensive overview of the diversity of interacting residue pairs in protein interfaces. We use a two-bead representation of each residue (i.e., backbone and side chain) to describe the pairs. However, a general analysis based on bead identities may not be sufficient, as different side chains can exhibit similar properties [15]. To address this limitation, we classify residue pairs based on their combined polarity and charge properties, which are commonly used in binding energy prediction methods [18], and we specify the charge type as either positive or negative. The table below summarizes the residue properties used. In addition, we adopt a more detailed approach by considering the protein regions, namely the interior, core and support, and rim. The rim is further subdivided into rim (interaction) and rim (NIS). Note that in contrast to our previous analyses, pairs involving backbone beads are excluded, as the backbone is a common feature of all residues.

Side chain beads	Class	Side chain beads	Property	Removed beads
ALA	apolar	LEU	apolar	Backbone
ARG	positive	LYS	positive	
ASN	polar	MET	apolar	
ASP	negative	PHE	apolar	
CYS	apolar	PRO	apolar	
GLN	polar	SER	polar	
GLU	negative	THR	polar	
GLY	apolar	TRP	apolar	
HIS	positive	TYR	polar	
ILE	apolar	VAL	apolar	

Table S5: The classification of individual residues is utilized to derive the properties of residue pairs. Backbone beads are excluded from the analysis.

To assess the diversity of residue pairs, we calculate the Shannon entropy (H) [21] for each protein region. This metric provides a quantitative measure of the information content, which reflects the degree of uncertainty or randomness in the data. This approach compares the frequency of each pair within a protein region, allowing us to evaluate the complexity of the region's pair composition. A low entropy value indicates that the region is composed of a limited diversity of pairs, making it more predictable. Conversely, a high entropy value suggests that the region exhibits a high diversity of pairs, rendering it more challenging to predict due to its randomness.

$$H_{pair\ in\ region} = -\text{Frequency}_{pair\ in\ region} \times \log_2(\text{Frequency}_{pair\ in\ region}) \tag{S6}$$

$$H_{region} = \sum H_{pair\ in\ region} \tag{S7}$$

The entropy value provides insight into the characteristics of the studied system. However, to determine if the system exhibits a random distribution, it is necessary to compare the observed entropy value with the maximum possible entropy (H_{max}). This comparison allows us to assess whether pair frequencies are uniformly distributed, indicating a completely random distribution. The difference (ΔH) between the system's entropy (H) and H_{max} indicates the extent to which the pair population deviates from a completely random distribution on the absolute scale. To characterize the distribution of pair classes independently of the number of categories considered, we also employ the evenness index (J) [22], which ranges from 0 to 1 and reflects the uniformity of pair frequencies within a protein region. A low J value indicates that a few pair classes dominate the population, whereas a high value suggests that the pair population is more homogeneous and evenly distributed.

$$H_{max} = -\frac{1}{N_{pair\ types}} \times \sum_{n=1}^{N_{pair\ types}} \log_2\left(\frac{1}{N_{pair\ types}}\right) \tag{S8}$$

$$= -\log_2\left(\frac{1}{N_{pair\ types}}\right) \tag{S9}$$

Based on the properties used to describe interacting residue pairs, as shown in Table S5, the value of $N_{pair\ types}$ *is equal to 10.*

$$\Delta H = H_{max} - H_{region} \tag{S10}$$

$$J = \frac{H_{region}}{H_{max}} \tag{S11}$$

I.2 Results

**Pair**	**Interior**	**Core & Support** (Stable)	**Core & Support** (Transient)	**Rim** (Stable)	**Rim** (Transient)
apolar-apolar	0.401	0.141	0.225	0.023	0.043
apolar-negative	0.119	0.121	0.131	0.127	0.150
apolar-polar	0.245	0.261	0.245	0.157	0.132
apolar-positive	0.111	0.123	0.154	0.141	0.140
negative-negative	0.015	0.046	0.033	0.145	0.139
negative-polar	0.024	0.073	0.041	0.062	0.048
negative-positive	0.011	0.037	0.032	0.091	0.092
polar-polar	0.038	0.088	0.052	0.061	0.024
polar-positive	0.026	0.077	0.048	0.077	0.068
positive-positive	0.010	0.034	0.038	0.116	0.163

Table S6: Frequencies of pair classes in the interior, core and support, and rim of stable and transient complexes.

J Identification of key amino acid pairs

Methods In this section, we use the same pair property descriptors as in SI section I. To identify key pairs, we draw inspiration from Natural Language Processing (NLP) methods, where residue pairs are treated as *words* or *word combinations*. Specifically, we derive two weighting functions: a modified term frequency-inverse document frequency (tf-idf) [23, 24] and the pointwise mutual information (PMI) [25, 26, 27]. The tf-idf metric characterizes the structural frequency of a pair, reflecting the number of times an interacting residue pair appears in the sub-dataset: interior, transient interface, and stable interface. Values below 1 indicate pairs widely represented across our sub-datasets.

$$\mathrm{tf}_{\mathrm{pair}} = \begin{cases} 1 + \log_{10} \mathrm{count}(\mathrm{pair}) & \text{if count(pair)} > 0 \\ 0 & \text{otherwise} \end{cases} \tag{S12}$$

$$\mathrm{df}_{\mathrm{pair}} = \text{Number of PDB where a given pair is found} \tag{S13}$$

$$\mathrm{idf}_{\mathrm{pair}} = \log_{10}\left(\frac{\text{Number of PDB in the sub-dataset}}{\mathrm{df}_{\mathrm{pair}}}\right) \tag{S14}$$

$$\textit{Where: } N_{PDB\ interior} = 413,\ N_{PDB\ stable\ interface} = 66,\ N_{PDB\ transient\ interface} = 94$$

$$\text{tf-idf}_{\mathrm{pair}} = \mathrm{tf}_{\mathrm{pair}} \times \mathrm{idf}_{\mathrm{pair}} \tag{S15}$$

The PMI score evaluates the pairing of residues by considering each residue as a *word* and their pairing as *co-occurrence*. This score compares the frequency of interacting pairs to the expected frequency of random associations. Consequently, a high positive value indicates a strong association between an interacting pair. In contrast, values below zero correspond to associations that only occur as often as expected by random chance or less. For the expected frequency calculation, we distinguish between homogeneous and heterogeneous pairs:

- Homogeneous pairs: composed of residues from the same class (e.g. polar–polar, nonpolar–nonpolar, negative–negative, positive–positive).
- Heterogeneous pairs: composed of residues from different classes (e.g. nonpolar–polar, nonpolar–negative).

The PMI is then calculated as:

$$\mathrm{PMI}_{\mathrm{pair}} \begin{cases} \log_2\left(\frac{Frequency_{pair}}{Frequency_{residue\ 1} \times Frequency_{residue\ 2}}\right) & \text{if homogeneous pair} \\ \log_2\left(\frac{Frequency_{pair}}{2 \times Frequency_{residue\ 1} \times Frequency_{residue\ 2}}\right) & \text{if heterogeneous pair} \end{cases} \tag{S16}$$

The PMI was calculated using mean pair-class and residue-class frequencies sampled from the respective regions of stable and transient complexes. Values for the interior were derived from structures in the whole dataset. Subsequently, all pairs with corresponding tf-idf values greater than 1 were removed from the PMI table, as their representation is limited across the dataset, making their interpretation unreliable due to insufficient structural data.

Results Table S7 show that there are 6 tf-idf values exceeding 1, indicating that the corresponding pair classes only occur in a limited set of protein complex structures. Notably, these pairs are predominantly located in the rim, which can be attributed to a high variability in pair composition within this region (see also SI section I). In contrast, all pair classes are widely represented in the core and support regions and for the interior.

	Interior	Core and Support Stable	Core and Support Transient	Rim Stable	Rim Transient
apolar-apolar	0.031	0.105	0.078	1.732*	1.215*
apolar-negative	0.080	0.103	0.210	0.821	0.649
apolar-polar	0.048	0.112	0.099	0.848	0.464
apolar-positive	0.108	0.130	0.234	0.539	0.575
negative-negative	0.514	0.596	0.819	0.838	0.666
negative-polar	0.183	0.313	0.565	1.029*	1.038*
negative-positive	0.412	0.341	0.662	0.815	0.797
polar-polar	0.152	0.234	0.446	1.313*	1.477*
polar-positive	0.195	0.176	0.485	0.941	0.837
positive-positive	0.676	0.605	0.860	0.884	0.656

Table S7: td-idf values for each pair class in the different protein regions. The values are considered on a regional basis (per-region), with all structures combined into a single dataset for each region. *: indicates values greater than 1 and used for PMI filtering.

	Interior	Core and Support Stable	Core and Support Transient	Rim Stable	Rim Transient
apolar-apolar	-0.105	-0.451	-0.322	-2.032*	-0.998*
apolar-negative	0.514	0.363	0.463	0.446	0.276
apolar-polar	-0.166	-0.098	0.044	-0.232	-0.038
apolar-positive	0.420	0.338	0.309	0.456	-0.086
negative-negative	0.915	1.351	1.184	1.681	1.503
negative-polar	-0.106	-0.043	-0.283	-0.406*	-0.556*
negative-positive	0.440	0.358	0.309	0.231	-0.163
polar-polar	-0.357	-0.335	-0.490	-0.822*	-1.675*
polar-positive	-0.063	0.114	-0.345	-0.620	-0.570
positive-positive	0.961	0.662	0.819	1.029	1.223

Table S8: PMI values of pair classes in the different protein regions. * Indicates values removed based on tf-idf filtering.

K Amino acid stickiness

Method Amino acid stickiness is a statistical free-energy scale that quantifies how overrepresented a particular amino acid species is at protein–protein interfaces compared to solvent-exposed protein surfaces [28]. This property captures the tendency of amino acid residues to preferentially localize to interfacial regions rather than remain exposed to solvent, reflecting the thermodynamic driving force for these residues to participate in protein–protein interactions. Stickiness s is calculated by comparing the fractional contribution f of each amino-acid type to the total interface area with its contribution to the overall solvent-accessible protein surface area, effectively measuring the relative enrichment of amino acids at interaction interfaces. Physically, stickiness is dominated by desolvation energy—the energetic cost associated with removing water molecules from around the amino acid side chain as it moves from an aqueous environment to participate in protein–protein contacts. This makes sticky residues particularly important for stabilizing protein complexes and driving the formation of functional protein assemblies, as they provide the thermodynamic foundation for favorable interactions between protein surfaces.

The contribution of a residue species to the buried surface area of an interface (BSA_{residue}) is computed separately for each protein region (core, support and rim) and is derived from the ASA values attributed to the residue species when the protein complex is formed (ASA_{bound}) and when free (ASA_{free}):

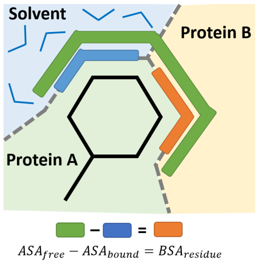

Figure S20: Schematic representation of the calculation of the interface area for a residue.

$$BSA_{\text{residue}} = ASA_{\text{free}} - ASA_{\text{bound}} \tag{S17}$$

The fractional contribution of a residue species (f_{residue}) compares the area formed by the given residue species with the whole area of the region considered. Here we distinguish between residues in the interface (core, support, or rim) and residues on the surface.

$$f_{\text{residue in interface region}} = \frac{BSA_{\text{residue in interface region}}}{\text{Total } BSA \text{ interface region}} \tag{S18}$$

$$f_{\text{residue on surface}} = \frac{ASA_{\text{residue on surface}}}{\text{Total } ASA \text{ surface}} \tag{S19}$$

The stickiness is derived from the fractional contribution of a residue species in the interface compared to its fractional contribution on the surface.

$$s_{\text{residue}} = \log\left(\frac{f_{\text{residue in interface region}}}{f_{\text{residue on surface}}}\right) \tag{S20}$$

Results

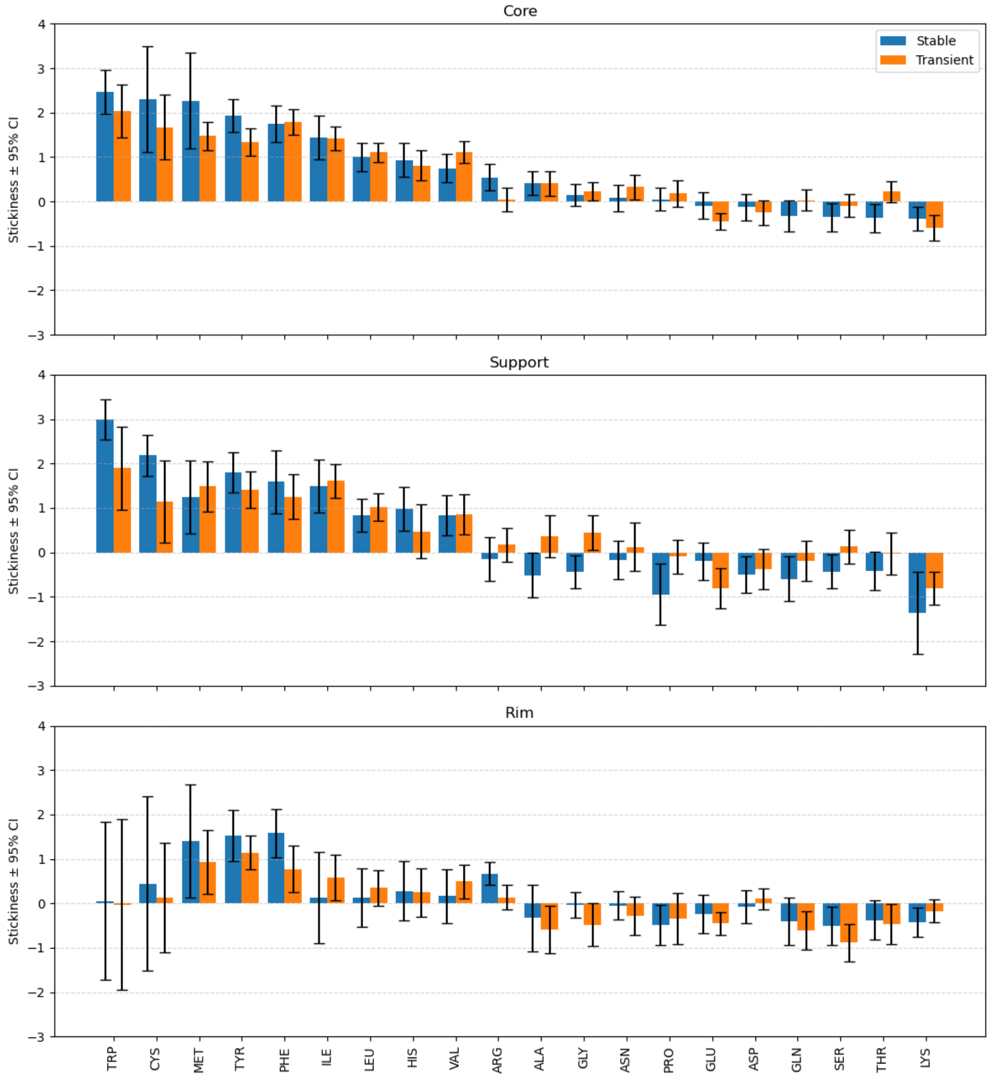

Figure S21: The stickiness values of each residue species have a similar profile for the core and support but differ in the rim. The mean values were computed across stable and transient complexes, respectively. The standard error of the mean with an appropriate Students t coefficient for a 95% confidence interval was used to estimate uncertainty.

L List of used structures

Temperature is set to 25.0 degrees Celsius and pH is set to 7.2 when unknown.

PDB	Database origin	Protein family	PubMed	Classification	Organism	Mutation	Method	Res. (Å)	UniprotKB	Affinity type	Affinity (M)	Chain 1	Chain 2	Temp. (C)	pH
1acb	BM5.5 PPI4DOC PDBind	PF00089 PF00280	1583684	hydrolase / hydrolase inhibitor	Bos taurus Hirudo medicinalis	No	x-ray diffraction	2.0	P00766 P01051	Kd	2e-10	E	I	25.0	7.2
1axi	PPI4DOC PDBind	PF00103 PF09067 PF00041	9353194	complex (hormone / receptor)	Homo sapiens	Yes	x-ray diffraction	2.1	P01241 P10912	Kd	1.4e-08	A	B	25.0	7.2
1ay7	BM5.5 PPI4DOC PDBind	PF00545 PF01337	9757110	complex (enzyme / inhibitor)	Kitasatospora aureofaciens Bacillus amyloliquefaciens	No	x-ray diffraction	1.7	P05798 P11540	Kd	1e-06	A	B	25.0	7.2
1dfj	BM5.5 PDBind	PF00074 PF13516 PF18779	7877692	complex (endonuclease / inhibitor)	Bos taurus Sus scrofa	No	x-ray diffraction	2.5	P61823 P10775	Ki	5.9e-14	E	I	25.0	6.0
1e6e	BM5.5	PF07992 PF00111	11053423	oxidoreductase	Bos taurus	Yes	x-ray diffraction	2.3	P08165 P00257	Kd	8.6e-07	A	B	25.0	7.4
1e96	BM5.5 PPI4DOC PDBind	PF00071 PF13181	11090627	signaling protein	Homo sapiens	Yes	x-ray diffraction	2.4	P63000 P19878	Kd	2.7e-06	A	B	18.0	7.0
1eer	BM5.5	PF00758 PF09067 PF00041	9774108	complex (cytokine / receptor)	Homo sapiens	Yes	x-ray diffraction	1.9	P01588 P19235	Kd	1e-09	A	BC	25.0	7.2
1efn	BM5.5 PPI4DOC	PF00018 PF00469	8681387	complex (sh3 domain / viral enhancer)	Homo sapiens Human immunodeficiency virus 1	Yes	x-ray diffraction	2.5	P06241 P03406	Kd	3.8e-07	B	A	25.0	7.2
1ewy	BM5.5 PPI4DOC	PF00175 PF00111	11053838	oxidoreductase	Nostoc sp. PCC 7119	No	x-ray diffraction	2.38	P21890 P0A3C8	Kd	3.57e-06	A	C	25.0	7.2
1f34	BM5.5 PPI4DOC PDBind	PF00026	10932249	hydrolase / hydrolase inhibitor	Sus scrofa Ascaris suum	Yes	x-ray diffraction	2.45	P00791 P19400	Kd	1e-10	A	B	25.0	7.2
1f3v	3DComplexV6 PDBind	PF09034 PF21355	10892748	apoptosis	Homo sapiens	No	x-ray diffraction	2.0	Q15628 Q12933	Kd	7.8e-06	0	1	25.0	7.2
1fle	BM5.5 PPI4DOC PDBind	PF00089 PF00095 PF10511	8794736	complex (serine protease / inhibitor)	Sus scrofa Homo sapiens	No	x-ray diffraction	1.9	P00772 P19957	Ki	6e-09	E	I	25.0	7.2
1fqj	BM5.5 PPI4DOC	PF00503 PF00615 PF00631 PF04868	11234020	signaling protein	Bos taurus Rattus norvegicus	No	x-ray diffraction	2.02	P10824 P04695 O46469 P04972	Kd	6.7e-08	A	B	25.0	7.2
1fy8	PDBind	PF00014	11420435	hydrolase / hydrolase inhibitor	Rattus rattus Bos taurus	No	x-ray diffraction	1.7	P00763 P00974	Ki	9e-06	0	1	25.0	7.2
1g9i	PDBind	PF00089	11257512	hydrolase / hydrolase inhibitor	Bos taurus Vigna radiata var. radiata	Yes	x-ray diffraction	2.2	P00760 P01062	Kd	1.2e-07	0	1	25.0	7.2
1gcq	BM5.5	PF00018 PF00018	11406576	signaling protein / signaling protein	Homo sapiens Mus musculus	No	x-ray diffraction	1.68	P62993 P27870	Kd	1.68e-05	B	C	25.0	7.4

PDB	Database origin	Protein family	PubMed	Classification	Organism	Mutation	Method	Res. (Å)	UniprotKB	Affinity type	Affinity (M)	Chain 1	Chain 2	Temp. (C)	pH
1grn	BM5.5<br>PDBind	PF00071<br>PF00620	9846874	gene regulation	Homo sapiens	No	x-ray diffraction	2.1	P60953<br>Q07960	Kd	3.88e-07	A	B	25.0	8.0
1h59	PDBind	PF00049<br>PF00219	11447105	insulin	Homo sapiens	No	x-ray diffraction	2.1	P05019<br>P24593	Kd	3.7e-08	0	1	25.0	7.2
1hia	BM5.5	PF00089<br>PF00089<br>PF02822	9032072	complex (protease / inhibitor)	Sus scrofa Hirudo medicinalis	No	x-ray diffraction	2.4	P00752<br>P00752<br>P80302	Ki	1.3e-08	AB	I	25.0	7.2
1i2m	BM5.5<br>PPI4DOC	PF00071<br>PF00415	11336674	cell cycle	Homo sapiens	No	x-ray diffraction	1.76	P62826<br>P18754	Kd	2.5e-12	A	B	25.0	7.2
1i8k	PPI4DOC	PF07686<br>PF07686	11352579	immune system	Mus musculus	Yes	x-ray diffraction	1.8	P01660<br>P18529	Kd	2.2e-08	A	B	25.0	7.2
1jiw	BM5.5<br>PDBind	PF13583<br>PF00353<br>PF08548<br>PF02974	11445573	hydrolase / hydrolase inhibitor	Pseudomonas aeruginosa	No	x-ray diffraction	1.74	Q03023<br>Q03026	Kd	4e-12	P	I	25.0	7.2
1jmo	BM5.5	PF09396<br>PF00089<br>PF00079	12169660	blood clotting	Homo sapiens	Yes	x-ray diffraction	2.2	P00734<br>P00734<br>P05546	Kd	1.15e-07	A	HL	25.0	7.2
1jps	BM5.5<br>PPI4DOC	PF09294	11601848	immune system	Homo sapiens	No	x-ray diffraction	1.85	P13726	Kd	1e-10	HL	T	25.0	7.4
1jtd	BM5.5<br>PPI4DOC<br>PDBind	PF13354<br>PF00144<br>PF13540	11573088	hydrolase / inhibitor	Escherichia coli Streptomyces exfoliatus	No	x-ray diffraction	2.3	P62593<br>O87916	Ki	2.72e-11	B	A	25.0	7.0
1ktz	BM5.5<br>PPI4DOC<br>PDBind	PF00019<br>PF08917	11850637	cytokine / cytokine receptor	Homo sapiens	No	x-ray diffraction	2.15	P10600<br>P37173	Kd	2.9e-07	A	B	25.0	7.2
1l4d	PPI4DOC<br>PDBind	PF00089<br>PF02821	12456874	hydrolase / hydrolase activator	Homo sapiens Streptococcus dysgalactiae subsp. equisimilis	Yes	x-ray diffraction	2.3	P00747<br>P00779	Kd	1.966e-07	A	B	25.0	7.2
1lp1	PDBind	PF02216	12604795	immune system	Staphylococcus aureus	Yes	x-ray diffraction	2.3	P38507	Kd	2e-06	0	1	25.0	7.2
1lw6	PDBind	PF00082<br>PF00280	12142461	hydrolase	Bacillus amyloliquefaciens Hordeum vulgare	Yes	x-ray diffraction	1.5	P00782<br>P01053	Kd	2e-12	0	1	25.0	7.2
1m1e	PDBind	PF00514<br>PF06384	12408825	structural protein	Mus musculus Homo sapiens	No	x-ray diffraction	2.1	Q02248<br>Q9NSA3	Kd	1e-09	0	1	25.0	7.2
1mzw	PDBind	PF00160<br>PF08799	12875835	isomerase	Homo sapiens	No	x-ray diffraction	2.0	O43447<br>O43172	Kd	1.97e-06	0	1	25.0	7.2
1nb5	PPI4DOC	PF00112<br>PF00031	12581647	hydrolase / hydrolase inhibitor	Sus scrofa Homo sapiens	No	x-ray diffraction	2.4	O46427<br>O46427<br>P01040	Ki	6.9e-11	A	C	25.0	7.2

PDB	Database origin	Protein family	PubMed	Classification	Organism	Mutation	Method	Res. (Å)	UniprotKB	Affinity type	Affinity (M)	Chain 1	Chain 2	Temp. (C)	pH
1nca	PPI4DOC	PF00064 PF07654	1381757	hydrolase(o-glycosyl)	Influenza A virus (A / tern / Australia / G70C / 1975(H11N9)) Mus musculus	No	x-ray diffraction	2.5	P03472 P01865	Kd	8.3e-09	HL	N	25.0	7.2
1nw9	BM5.5 PDBind	PF00653 PF00656	12620238	apoptosis	Homo sapiens	No	x-ray diffraction	2.4	P98170 P55211	Ki	1.3e-08	B	A	25.0	7.2
1oc0	BM5.5 PPI4DOC PDBind	PF00079 PF01033	12808446	hydrolase / inhibitor	Homo sapiens	Yes	x-ray diffraction	2.28	P05121 P04004	Kd	1e-09	A	B	25.0	7.2
1op9	PDBind	PF00062	12917687	hydrolase	Camelus dromedarius Homo sapiens	No	x-ray diffraction	1.86	P61626	Kd	7e-10	0	1	25.0	7.2
1oyv	BM5.5 PPI4DOC	PF00082 PF02428	12684499	hydrolase	Bacillus licheniformis Solanum lycopersicum	No	x-ray diffraction	2.5	P00780 P05119	Ki	9e-09	B	I	25.0	7.2
1pk1	PPI4DOC	PF07647 PF00536	15905166	transcription repression	Drosophila melanogaster	Yes	x-ray diffraction	1.8	P39769 Q9VHA0	Kd	5.4e-08	A	B	25.0	7.2
1pvh	BM5.5 PPI4DOC	PF00041 PF09240 PF01291	14527405	signaling protein / cytokine	Homo sapiens	No	x-ray diffraction	2.5	P40189 P15018	Kd	8e-08	A	B	25.0	7.2
1pxv	BM5.5 PPI4DOC	PF05543 PF09023	12874290	hydrolase	Staphylococcus aureus	Yes	x-ray diffraction	1.8	P0C1S6 Q9EYW6	Ki	3.1e-10	A	C	25.0	7.2
1r6q	BM5.5	PF02861 PF02617	15037248	chaperone / protein binding	Escherichia coli	No	x-ray diffraction	2.35	P0ABH9 P0A8Q6	Kd	3.3e-07	A	C	25.0	7.2
1rjc	PPI4DOC PDBind	PF00062	15659390	immune system / hydrolase	Camelus dromedarius Gallus gallus	No	x-ray diffraction	1.4	P00698	Kd	7.7e-11	A	B	25.0	7.2
1rv6	BM5.5 PPI4DOC	PF00341 PF21339	14684734	hormone / growth factor / receptor	Homo sapiens	No	x-ray diffraction	2.45	P49763 P17948	IC50	2.75e-07	VW	X	25.0	7.2
1sbb	BM5.5	PF07654 PF01123 PF02876	9881971	immune system	Mus musculus Staphylococcus aureus	Yes	x-ray diffraction	2.4	P01852 P01552	Kd	0.00014	A	B	25.0	7.2
1sv0	PPI4DOC	PF02198 PF02198	15260987	transcription	Drosophila melanogaster	Yes	x-ray diffraction	2.07	Q01842 Q7K119	Kd	1.11e-08	A	B	25.0	7.2
1t0p	PPI4DOC PDBind	PF00092 PF03921	15728350	immune system	Homo sapiens	Yes	x-ray diffraction	1.66	P20701 P32942	Kd	2.5e-05	A	B	25.0	7.2
1t5z	PDBind	PF00104 PF12489	15563469	hormone / growth factor	Homo sapiens	No	x-ray diffraction	2.3	P10275 Q13772	Kd	3.3e-05	0	1	25.0	7.2
1t6b	BM5.5 PPI4DOC 3DComplexV6 PDBind	PF07691 PF17475 PF03495 PF17476 PF20835 PF00092	15243628	membrane protein / toxin	Bacillus anthracis Homo sapiens	No	x-ray diffraction	2.5	P13423 P58335	Kd	4e-10	X	Y	25.0	7.4

PDB	Database origin	Protein family	PubMed	Classification	Organism	Mutation	Method	Res. (Å)	UniprotKB	Affinity type	Affinity (M)	Chain 1	Chain 2	Temp. (C)	pH
1ta3	PPI4DOC PDBind	PF00704 PF00331	15181003	hydrolase inhibitor / hydrolase	Triticum aestivum Aspergillus nidulans	No	x-ray diffraction	1.7	Q8L5C6 Q00177	Ki	9e-09	A	B	25.0	7.2
1u0s	PPI4DOC PDBind	PF00072 PF07194	15289606	signaling protein	Thermotoga maritima	No	x-ray diffraction	1.9	Q56312 Q56310	Kd	2.3e-07	A	Y	25.0	7.2
1uad	PPI4DOC	PF00071 PF01833	12839989	endocytosis / exocytosis	Homo sapiens Rattus norvegicus	No	x-ray diffraction	2.1	P11233 O54921	Kd	1.37e-07	A	B	25.0	7.2
1v7p	PPI4DOC	PF00059 PF00059 PF00092	15276841	toxin / cell adhesion	Echis multisquamatus Homo sapiens	Yes	x-ray diffraction	1.9	Q7T2Q1 Q7T2Q0 P17301	IC50	6e-09	A	C	25.0	7.2
1veu	PDBind	PF08923 PF03259	15263099	signaling protein / protein binding	Mus musculus	Yes	x-ray diffraction	2.15	O88653 Q9JHS3	Kd	1.28e-08	0	1	25.0	7.2
1vg0	PPI4DOC PDBind	PF00996 PF00071	15186776	protein binding / protein transport	Rattus norvegicus	Yes	x-ray diffraction	2.2	P37727 P09527	Kd	5e-09	A	B	25.0	7.2
1vrk	PDBind		10194305	complex(calcium-binding protein / peptide)	synthetic construct Gallus gallus	No	x-ray diffraction	1.9	P11799	Kd	6.8e-09	0	1	25.0	7.2
1wej	BM5.5 PPI4DOC	PF07686 PF00034	9698550	complex (antibody / electron transport)	Mus musculus Equus caballus	No	x-ray diffraction	1.8	P01635 P00004	Kd	6e-08	HL	F	25.0	7.2
1wlp	PDBind	PF05038 PF00018	16326715	oxidoreductase / signaling protein	Homo sapiens	No	solution nmr		P13498 P14598	Kd	6.4e-07	0	1	25.0	7.2
1xg2	PPI4DOC 3DComplexV6 PDBind	PF01095 PF04043	15722470	hydrolase / hydrolase inhibitor	Solanum lycopersicum Actinidia chinensis	No	x-ray diffraction	1.9	P14280 P83326	Kd	5e-09	A	B	25.0	7.2
1xt9	PDBind	PF02902 PF00240	15567417	hydrolase / hydrolase inhibitor	Homo sapiens	No	x-ray diffraction	2.2	Q96LD8 Q15843	Kd	2e-07	0	1	25.0	7.2
1z0k	BM5.5	PF00071 PF11464	16034420	protein transport	Homo sapiens	Yes	x-ray diffraction	1.92	P20338 Q9H1K0	Kd	7.7e-06	A	B	25.0	7.5
1z7x	PPI4DOC	PF00074 PF13516 PF18779	17350650	hydrolase / hydrolase inhibitor	Homo sapiens	No	x-ray diffraction	1.95	P07998 P13489	Kd	2.9e-16	Y	Z	25.0	7.2
1ze3	PPI4DOC	PF02753 PF00345 PF00419 PF13954	15920478	chaperone / structural / membrane protein	Escherichia coli	No	x-ray diffraction	1.84	P31697 P08191 P30130	Kd	1.2e-06	C	H	25.0	7.2
1zli	BM5.5 PDBind	PF00246 PF10468	15961103	hydrolase / hydrolase inhibitor	Homo sapiens Rhipicephalus bursa	No	x-ray diffraction	2.09	P15086 Q5EPH2	Ki	1.3e-09	A	B	25.0	7.2
2a7u	PDBind	PF00213	16128580	hydrolase	Escherichia coli O157:H7	No	solution nmr		P0ABB2 P0ABA5	Kd	1.2e-07	0	1	25.0	7.2
2arp	PDBind	PF00019 PF09289 PF07648	16482217	hormone / growth factor	Homo sapiens Rattus norvegicus	No	x-ray diffraction	2.0	P08476 P21674	Kd	4.3e-07	0	1	25.0	7.2
2ast	PPI4DOC	PF03931 PF01466 PF12937 PF01111	16209941	ligase / ligase inhibitor	Homo sapiens	Yes	x-ray diffraction	2.3	P63208 Q13309 P61024 P46527	Kd	7e-06	C	B	25.0	7.2

PDB	Database origin	Protein family	PubMed	Classification	Organism	Mutation	Method	Res. (Å)	UniprotKB	Affinity type	Affinity (M)	Chain 1	Chain 2	Temp. (C)	pH
2b42	BM5.5 PDBind	PF14541 PF14543 PF00457	19769747	hydrolase inhibitor / hydrolase	Triticum aestivum Bacillus subtilis	No	x-ray diffraction	2.5	Q8H0K8 P18429	Kd	1.07e-09	B	A	22.0	5.0
2b7c	PDBind	PF00009 PF03143 PF03144 PF00736	16675455	translation	Saccharomyces cerevisiae	Yes	x-ray diffraction	1.8	P02994 P32471	Kd	4e-07	0	1	25.0	7.2
2c0l	BM5.5 PPI4DOC PDBind	PF13181 PF02036	17157249	transport protein / receptor	Homo sapiens	No	x-ray diffraction	2.3	P50542 P22307	Kd	1.09e-07	A	B	35.0	7.4
2c1m	PDBind	PF00514 PF01749 PF16186 PF08911	16222336	protein transport / membrane protein	Mus musculus	No	x-ray diffraction	2.2	P52293 Q9JIH2	Kd	1.1e-09	0	1	25.0	7.2
2cjs	PPI4DOC	PF00168 PF02318	16732694	exocytosis	Rattus norvegicus	Yes	x-ray diffraction	1.78	Q62768 Q9JIS1	Kd	1e-07	B	C	25.0	7.2
2dd8	BM5.5 PPI4DOC	PF07686 PF07654 PF16451 PF09408	16597622	immune system / viral protein	Homo sapiens Severe acute respiratory syndrome-related coronavirus	No	x-ray diffraction	2.3	Q8N355 P59594	Kd	2e-08	HL	S	25.0	7.2
2dsp	PDBind	PF00219 PF00049	16924115	protein binding / hormone / growth factor	Homo sapiens	No	x-ray diffraction	2.5	P22692 Q9NP10	Kd	3e-06	0	1	25.0	7.2
2es4	3DComplexV6	PF00561 PF03280	16518399	hydrolase	Burkholderia glumae	Yes	x-ray diffraction	1.85	P0DUB8 Q05490	Kd	5e-09	A	B	25.0	7.2
2f31	PDBind	PF06371 PF06367	16472745	structural protein	Mus musculus	No	x-ray diffraction	2.1	Q3US76 O08808	Kd	1.02e-07	0	1	25.0	7.2
2f4m	PDBind	PF01841 PF09280	16500903	hydrolase	Mus musculus	No	x-ray diffraction	1.85	Q9JI78 P54728	Kd	6.5e-08	0	1	25.0	7.2
2f5z	PPI4DOC	PF07992 PF02852 PF02817	16442803	oxidoreductase / protein binding	Homo sapiens	Yes	x-ray diffraction	2.18	P09622 O00330	Kd	7.8e-10	B	C	25.0	7.2
2f9z	3DComplexV6	PF04509 PF03975	16469702	signaling protein	Thermotoga maritima MSB8	No	x-ray diffraction	2.4	Q9X006 Q9X005	Kd	9e-07	A	C	25.0	7.2
2fdb	PPI4DOC	PF00167 PF13927 PF07679	16384934	hormone / growth factor / transferase	Homo sapiens	Yes	x-ray diffraction	2.28	P55075 P21802	Kd	1.55e-07	M	P	25.0	7.2
2few	PDBind	PF00359 PF02302	16443929	transferase	Escherichia coli Escherichia coli O157:H7	Yes	solution nmr		P00550 P00550	Kd	0.0037	0	1	25.0	7.2
2fu5	PPI4DOC	PF04421 PF00071	16541104	signaling protein	Homo sapiens Mus musculus	No	x-ray diffraction	2.0	P47224 P55258	Kd	7e-10	A	B	25.0	7.2
2fyl	PDBind	PF06400 PF00057	16938309	surface active protein	Homo sapiens	No	solution nmr		P30533 Q07954	Kd	2.8e-06	0	1	25.0	7.2
2gng	PDBind	PF00069 PF02827	16699172	transferase / transferase inhibitor	Bos taurus Mus musculus	Yes	x-ray diffraction	1.87	P00517 P63248	IC50	6e-06	0	1	25.0	7.2

PDB	Database origin	Protein family	PubMed	Classification	Organism	Mutation	Method	Res. (Å)	UniprotKB	Affinity type	Affinity (M)	Chain 1	Chain 2	Temp. (C)	pH
2hev	PPI4DOC PDBind	PF00020	16905106	cytokine	Homo sapiens	Yes	x-ray diffraction	2.41	P23510 P43489	IC50	6.2e-08	R	F	25.0	7.2
2hqs	BM5.5 PPI4DOC	PF04052 PF07676 PF00691	17375930	transport protein / lipoprotein	Escherichia coli	No	x-ray diffraction	1.5	P0A855 P0A912	Kd	2.7e-08	A	H	25.0	7.2
2hqw	PDBind	PF13499	18073110	metal binding protein	Rattus norvegicus	No	x-ray diffraction	1.9	P0DP29 P35439	Kd	2e-09	0	1	25.0	7.2
2hrk	BM5.5 PPI4DOC PDBind		16914447	ligase / rna binding protein	Saccharomyces cerevisiae	No	x-ray diffraction	2.05	P46655 P46672	Kd	9e-09	A	B	25.0	7.2
2ij0	PPI4DOC	PF07686	17268555	protein binding	Staphylococcus aureus Homo sapiens	No	x-ray diffraction	2.25	P06886 A0A5B4	Kd	1.8e-10	A	B	25.0	7.2
2j1k	PPI4DOC	PF07686	16923808	virus / receptor	Homo sapiens Canine adenovirus 2	No	x-ray diffraction	2.3	P78310 Q65914	Kd	1.1e-09	F	C	25.0	7.2
2j4w	PPI4DOC	PF02430 PF07686 PF07654 PF07686 PF07654	17229439	immune system	Plasmodium vivax Mus musculus	Yes	x-ray diffraction	2.5	Q9TY14 Q569B4 Q5XFY8	Kd	6e-10	HL	D	25.0	7.2
2j59	PPI4DOC	PF00025 PF00169	17347647	hydrolase	Mus musculus Homo sapiens	Yes	x-ray diffraction	2.1	P84078 Q5T5U3	Kd	5.5e-08	A	B	25.0	7.2
2j7p	BM5.5	PF00448 PF02881 PF00448	17184999	signal recognition	Thermus aquaticus	No	x-ray diffraction	1.97	O07347 P83749	Kd	1e-08	A	D	25.0	7.2
2j8x	PPI4DOC	PF03167 PF18880	17157317	hydrolase / inhibitor	human gammaherpesvirus 4 Bacillus phage PBS2	No	x-ray diffraction	2.3	P12888 P14739	Ki	8e-09	A	B	25.0	7.2
2jby	PDBind	PF11099 PF00452	17386268	apoptosis	Myxoma virus Homo sapiens	No	x-ray diffraction	2.41	Q85295 Q16611	IC50	5e-08	0	1	25.0	7.2
2jod	PDBind	PF02793 PF00123	17470806	signaling protein	Homo sapiens	Yes	solution nmr		P41586 P18509	Ki	3.5e-07	0	1	25.0	7.2
2k2s	PDBind	PF11476 PF12661	18818666	cell adhesion	Toxoplasma gondii	No	solution nmr		O00834 Q9XYH7	Kd	5.3e-08	0	1	25.0	7.2
2k79	PDBind	PF00018 PF00017	19361414	transferase	Mus musculus	No	solution nmr		Q03526 Q03526	Kd	0.00067	0	1	25.0	7.2
2mp0	PDBind	PF00391 PF05524 PF00358	25131700	transferase	Escherichia coli K-12	No	solution nmr		P08839 P69783	Kd	0.025	0	1	25.0	7.2
2o3b	BM5.5 PDBind	PF01223 PF07924	17138564	hydrolase / hydrolase inhibitor	Nostoc sp. PCC 7120 = FACHB-418	Yes	x-ray diffraction	2.3	P38446 Q7A260	Ki	3.2e-12	A	B	25.0	7.2
2omu	PDBind	PF00028	17715295	cell invasion / cell adhesion	Listeria monocytogenes EGD-e Homo sapiens	Yes	x-ray diffraction	1.8	P12830	Kd	6e-10	0	1	25.0	7.2
2oob	BM5.5 PPI4DOC PDBind	PF00240	17679095	ligase	Homo sapiens Bos taurus	No	x-ray diffraction	1.9	Q13191 P0CH28	Kd	5.7e-05	A	B	25.0	7.0

PDB	Database origin	Protein family	PubMed	Classification	Organism	Mutation	Method	Res. (Å)	UniprotKB	Affinity type	Affinity (M)	Chain 1	Chain 2	Temp. (C)	pH
2oor	BM5.5	PF05222 PF01262 PF02233	17323922	oxidoreductase	Rhodospirillum rubrum	No	x-ray diffraction	2.32	Q2RSB2 P0C188	Kd	1.55e-08	AB	C	25.0	7.2
2ot3	BM5.5 PPI4DOC PDBind	PF18151 PF02204 PF00071	17450153	protein transport	Homo sapiens	No	x-ray diffraction	2.1	Q9UJ41 Q9UL25	Kd	1.8e-06	B	A	25.0	7.2
2p8q	PDBind	PF00514 PF03810 PF11538	18187419	protein transport	Homo sapiens	No	x-ray diffraction	2.35	Q14974 O95149	Kd	1.56e-08	0	1	25.0	7.2
2pcc	BM5.5	PF00141 PF00034	1334573	oxidoreductase / electron transport	Saccharomyces cerevisiae	No	x-ray diffraction	2.3	P00431 P00044	Kd	1.6e-06	A	B	25.0	7.2
2ptt	PPI4DOC PDBind	PF07686 PF11465	17950006	immune system	Mus musculus	Yes	x-ray diffraction	1.63	P18181 Q07763	Kd	4e-06	A	B	25.0	7.2
2q0o	PPI4DOC	PF03472 PF00196 PF09228	17921255	transcription	Sinorhizobium fredii NGR234	No	x-ray diffraction	2.0	P55407 P55408	Kd	1.49e-08	A	C	25.0	7.2
2qc1	PPI4DOC	PF02931	17643119	protein binding	Bungarus multicinctus Mus musculus	Yes	x-ray diffraction	1.94	P60616 P04756	Kd	1e-11	A	B	25.0	7.2
2qxv	PDBind	PF00400 PF11616	17937919	gene regulation	Mus musculus	No	x-ray diffraction	1.82	Q921E6 Q61188	Kd	3.8e-07	0	1	25.0	7.2
2uyz	PPI4DOC PDBind	PF00179 PF11976	17491593	ligase	Mus musculus Homo sapiens	Yes	x-ray diffraction	1.4	P63280 P63165	Kd	8.2e-08	A	B	25.0	7.2
2v3b	PPI4DOC PDBind	PF07992 PF18113 PF00301	17636129	oxidoreductase	Pseudomonas aeruginosa PAO1	No	x-ray diffraction	2.45	Q9HTK9 Q9HTK8	Kd	5e-06	A	B	25.0	7.2
2v5q	PPI4DOC	PF00069	18391401	transferase	Homo sapiens synthetic construct	No	x-ray diffraction	2.3	P53350	Kd	7.1e-08	A	B	25.0	7.2
2v6x	PDBind	PF04212 PF03357	17928861	protein transport	Saccharomyces cerevisiae	No	x-ray diffraction	1.98	P52917 P36108	Kd	2.8e-05	0	1	25.0	7.2
2v8s	PPI4DOC PDBind	PF01417 PF05008	18033301	protein transport	Homo sapiens	No	x-ray diffraction	2.22	Q14677 Q9UEU0	Kd	2.2e-05	E	V	25.0	7.2
2ver	PDBind	PF04619 PF07686	18086185	cell adhesion	Escherichia coli Homo sapiens	No	solution nmr		Q57254 P06731	Kd	1.31e-05	0	1	25.0	7.2
2vln	PDBind	PF01320 PF21431	18471830	protein binding	Escherichia coli	Yes	x-ray diffraction	1.6	P13479 P09883	Kd	1.68e-12	0	1	25.0	7.2
2vog	PDBind	PF00452 PF15185	18462686	apoptosis	Mus musculus	Yes	x-ray diffraction	1.9	Q07440 Q91ZE9	Kd	2.1e-07	0	1	25.0	7.2
2w2x	PPI4DOC	PF00071 PF00018 PF00018	19394299	signaling protein / hydrolase	Homo sapiens	Yes	x-ray diffraction	2.3	P15153 P16885 P16885	Kd	2.19e-05	A	B	25.0	7.2
2wel	PDBind	PF00069 PF13499	20668654	transferase	Homo sapiens	No	x-ray diffraction	1.9	Q13557 P0DP23	Kd	3e-07	0	1	25.0	7.2
2wwk	PDBind	PF07679 PF07679	20133654	transferase / structural protein	Homo sapiens	Yes	x-ray diffraction	1.7	O75147 Q8WZ42	Kd	1.28e-06	0	1	25.0	7.2

PDB	Database origin	Protein family	PubMed	Classification	Organism	Mutation	Method	Res. (Å)	UniprotKB	Affinity type	Affinity (M)	Chain 1	Chain 2	Temp. (C)	pH
2wy8	PDBind	PF07678	21055811	immune system	Homo sapiens Staphylococcus aureus	Yes	x-ray diffraction	1.7	P01024	Kd	3.6e-07	0	1	25.0	7.2
2x9a	BM5.5 PPI4DOC 3DComplexV6	PF05357 PF06519	21110981	viral protein	Escherichia phage If1 Escherichia coli	No	x-ray diffraction	2.47	O80297 Q8X965	Kd	4.4e-06	D	C	25.0	7.0
2xgy	PPI4DOC PDBind	PF00160	20833376	viral protein / isomerase	Oryctolagus cuniculus Homo sapiens	No	x-ray diffraction	1.8	P62937	Kd	3e-05	A	B	25.0	7.2
2yvj	BM5.5 PPI4DOC	PF07992 PF14759 PF00355	17850818	oxidoreductase / electron transport	Pseudomonas sp.	No	x-ray diffraction	1.9	Q52437 Q52440	Kd	0.000294	A	B	25.0	7.2
2z3q	PPI4DOC	PF02372	17643103	cytokine / cytokine receptor	Homo sapiens	No	x-ray diffraction	1.85	P40933 Q13261	Kd	3.8e-11	A	B	25.0	7.2
2z58	PDBind	PF00082	17988685	hydrolase	Thermococcus kodakarensis KOD1	Yes	x-ray diffraction	1.88	P58502 P58502	Kd	1.6e-08	0	1	25.0	7.2
2z7f	PPI4DOC	PF00089 PF00095	18421166	hydrolase / hydrolase inhibitor	Homo sapiens	No	x-ray diffraction	1.7	P08246 P03973	IC50	1.3e-08	E	I	25.0	7.2
3aaa	BM5.5	PF01267 PF01115 PF12796	20625546	protein binding	Gallus gallus Homo sapiens	No	x-ray diffraction	2.2	P13127 P14315 P58546	Kd	2.1e-08	AB	C	20.0	7.0
3ajb	PDBind	PF04882	21102411	protein transport	Homo sapiens	Yes	x-ray diffraction	2.5	P56589 P40855	Kd	4.08e-08	0	1	25.0	7.2
3aon	PDBind	PF01813 PF01990	22114184	hydrolase	Enterococcus hirae	No	x-ray diffraction	2.0	P43435 P43455	Kd	3.2e-09	0	1	25.0	7.2
3au4	PDBind	PF00788 PF00373 PF00784 PF06583	21642953	motor protein / apoptosis	Homo sapiens	No	x-ray diffraction	1.9	Q9HD67 P43146	Kd	5.3e-07	0	1	25.0	7.2
3blh	PDBind	PF00069 PF00134 PF21797	18566585	transcription	Homo sapiens	Yes	x-ray diffraction	2.48	P50750 O60563	Kd	3e-07	0	1	25.0	7.2
3bx1	PPI4DOC	PF00082 PF00197	18556023	hydrolase / hydrolase inhibitor	Lederbergia lenta Hordeum vulgare	No	x-ray diffraction	1.85	P29600 P07596	Kd	1.3e-06	A	B	25.0	7.2
3c4p	PDBind	PF13354 PF00144 PF07467	18775544	hydrolase / hydrolase inhibitor	Klebsiella pneumoniae Streptomyces clavuligerus	Yes	x-ray diffraction	1.75	P0AD64 P35804	Kd	4.4e-09	0	1	25.0	7.2
3c5t	PDBind	PF02793 PF00123	18287102	signaling protein / signaling protein	Homo sapiens Heloderma suspectum	No	x-ray diffraction	2.1	P43220 P26349	IC50	6e-10	0	1	25.0	7.2
3c9a	PPI4DOC	PF11581	18500331	hormone / signaling protein	Drosophila melanogaster	No	x-ray diffraction	1.6	Q00805 Q01083	Kd	7.7e-09	A	B	25.0	7.2
3cqx	PPI4DOC	PF00012	19029896	chaperone	Mus musculus	No	x-ray diffraction	2.3	P63017 Q91YN9	Kd	4.5e-06	B	C	25.0	7.2
3cx7	PDBind	PF00503 PF09128	18940608	signaling protein	Mus musculus Rattus norvegicus	No	x-ray diffraction	2.25	P27601 Q9ES67	Kd	5.2e-07	0	1	25.0	7.2

PDB	Database origin	Protein family	PubMed	Classification	Organism	Mutation	Method	Res. (Å)	UniprotKB	Affinity type	Affinity (M)	Chain 1	Chain 2	Temp. (C)	pH
3d2u	PPI4DOC	PF00129 PF07654 PF07654 PF13895 PF00022	18632577	immune system	Human betaherpesvirus 5 Homo sapiens	Yes	x-ray diffraction	2.21	P08560 P61769 Q8NHL6 P60709	Kd	4e-09	C	B	25.0	7.2
3ddc	PDBind	PF00071	18596699	hydrolase / apoptosis	Homo sapiens Mus musculus	Yes	x-ray diffraction	1.8	P01112 Q5EBH1	Kd	7.7e-07	0	1	25.0	7.2
3doe	PDBind	PF00025 PF11527	19368893	signaling protein / hydrolase	Homo sapiens	No	x-ray diffraction	2.25	P36404 Q9Y2Y0	Kd	2e-08	0	1	25.0	7.2
3e1z	PPI4DOC PDBind	PF09394 PF00112	19143838	hydrolase inhibitor / hydrolase	Trypanosoma cruzi Carica papaya	No	x-ray diffraction	1.86	Q966X9 P00784	Ki	3.6e-11	A	B	25.0	7.2
3fju	PDBind	PF00246 PF15270	19179285	hydrolase / hydrolase inhibitor	Homo sapiens Ascaris suum	No	x-ray diffraction	1.6	P15085 P19399	Ki	1.6e-09	0	1	25.0	7.2
3fpu	PDBind	PF19429 PF00048	20041127	immune system	Rhipicephalus sanguineus Homo sapiens	Yes	x-ray diffraction	1.76	P0C8E7 P10147	Kd	1.2e-10	0	1	25.0	7.2
3gc3	3DComplexV6 PDBind	PF00339 PF02752 PF09268 PF13838 PF01394	19710023	endocytosis	Bos taurus	No	x-ray diffraction	2.2	P17870 P49951	Kd	2.1e-06	0	1	25.0	7.2
3gmw	PPI4DOC	PF13354 PF00144 PF07467	19332077	protein binding	Escherichia sp. Sflu5 Streptomyces exfoliatus	No	x-ray diffraction	2.1	A5PHA6 Q9KJ90	Kd	5e-11	A	B	25.0	7.2
3gni	PPI4DOC PDBind	PF08569 PF00069	19513107	signaling protein / signaling protein	Homo sapiens	No	x-ray diffraction	2.35	Q9Y376 Q7RTN6	Kd	1.2e-08	A	B	25.0	7.2
3gqi	PPI4DOC PDBind	PF07714 PF00017	19665973	transferase / transferase inhibitor	Homo sapiens Rattus norvegicus	Yes	x-ray diffraction	2.5	P11362 P10686	Kd	3.3e-08	A	B	25.0	7.2
3gxu	PDBind	PF01404 PF00812	19875447	transferase	Homo sapiens	No	x-ray diffraction	2.5	P54764 P52799	Kd	2.03e-07	0	1	25.0	7.2
3h42	SabDab	PF05922 PF18463 PF18464 PF18459	19443683	hydrolase / immune system	Homo sapiens	Yes	x-ray diffraction	2.3	Q8NBP7 Q8NBP7	Kd	4e-12	HL	BA	25.0	7.2
3h6s	PPI4DOC	PF00112 PF10467	19846555	hydrolase / hydrolase inhibitor	Homo sapiens Clitocybe nebularis	Yes	x-ray diffraction	2.22	O60911 Q3Y9I6	Ki	8e-11	A	B	25.0	7.2
3hh2	PPI4DOC	PF00019 PF09289 PF21333 PF07648	19644449	signaling protein / cytokine	Mus musculus Homo sapiens	No	x-ray diffraction	2.15	O08689 P19883	Kd	1.23e-08	A	B	25.0	7.2
3ixe	PDBind	PF12796 PF00412	19963065	signaling protein / signaling protein	Homo sapiens	No	x-ray diffraction	1.9	Q13418 Q7Z4I7	IC50	2.3e-06	0	1	25.0	7.2
3k1r	PDBind	PF21219 PF00595 PF00536	20142502	structural protein	Homo sapiens	Yes	x-ray diffraction	2.3	Q9Y6N9 Q495M9	Kd	1e-09	0	1	25.0	7.2
3k2m	PPI4DOC	PF00017	20357770	signaling protein / protein binding	Homo sapiens	No	x-ray diffraction	1.75	P00519	Kd	7e-09	A	B	25.0	7.2

PDB	Database origin	Protein family	PubMed	Classification	Organism	Mutation	Method	Res. (Å)	UniprotKB	Affinity type	Affinity (M)	Chain 1	Chain 2	Temp. (C)	pH
3kr3	PPI4DOC	PF00049	20515953	immune system	Homo sapiens	No	x-ray diffraction	2.2	P01344	Kd	4.9e-11	HL	D	25.0	7.2
3kuc	PDBind	PF00071 PF02196	20361980	gtp binding protein / transferase	Homo sapiens	Yes	x-ray diffraction	1.92	P62834 P04049	Kd	4.42e-07	0	1	25.0	7.2
3l5w	BM5.5	PF03487	20226193	immune system	Mus musculus Homo sapiens	No	x-ray diffraction	2.0	P35225	Kd	5.2e-11	LH	I	25.0	7.4
3l95	PPI4DOC	PF06816 PF07684 PF00066	20393564	immune system	Homo sapiens	No	x-ray diffraction	2.19	P46531	Kd	2.5e-09	HL	X	25.0	7.2
3l9j	PDBind	PF00059 PF00229	20179326	immune system	Homo sapiens	No	x-ray diffraction	2.1	P05452 P01375	Kd	3.4e-10	0	1	25.0	7.2
3lev	PPI4DOC	PF00140 PF04542	20876137	immune system	Thermus aquaticus Homo sapiens	Yes	x-ray diffraction	2.5	Q9EZJ8	Kd	6e-10	HL	A	25.0	7.2
3m63	PDBind	PF10408 PF04564 PF00240	20427284	ligase / protein binding	Saccharomyces cerevisiae	Yes	x-ray diffraction	2.4	P54860 P48510	Kd	1.75e-07	0	1	25.0	7.2
3mc0	PPI4DOC	PF07686 PF01123 PF02876	21059660	immune system	Mus musculus Staphylococcus aureus	Yes	x-ray diffraction	2.0	A2NTY6 D0EMB6	Kd	1.25e-07	A	B	25.0	7.2
3mxw	BM5.5	PF01085 PF01079	20504762	signaling protein	Homo sapiens	No	x-ray diffraction	1.83	Q15465	Kd	7e-10	LH	A	30.0	7.2
3mzg	PDBind	PF00103 PF09067	20889499	hormone / hormone receptor	Homo sapiens	Yes	x-ray diffraction	2.1	P01236 P16471	Kd	0.00497	0	1	25.0	7.2
3nvn	PPI4DOC PDBind	PF01403 PF01437	20727575	viral protein / signaling protein	Ectromelia virus Homo sapiens	No	x-ray diffraction	2.26	Q8JL80 O60486	Kd	9.4e-09	A	B	25.0	7.2
3o34	PDBind	PF00439 PF00628 PF00125	21164480	transcription / protein binding	Homo sapiens	No	x-ray diffraction	1.9	O15164 P68431	Kd	8.8e-06	0	1	25.0	7.2
3o5t	PDBind	PF03747 PF00543	22074780	hydrolase / transcription	Azospirillum brasilense	No	x-ray diffraction	2.09	A7XNI2 P70731	Kd	1.5e-06	0	1	25.0	7.2
3oiq	PDBind	PF16853	20877309	protein binding	Saccharomyces cerevisiae	No	x-ray diffraction	2.4	P32797 P13382	Kd	3.8e-06	0	1	25.0	7.2
3oky	PPI4DOC	PF01437 PF01403 PF17960 PF01437 PF01403	20877282	signaling protein	Mus musculus	No	x-ray diffraction	2.19	P70207 O35464	Kd	1.3e-06	A	B	25.0	7.2
3orv	PPI4DOC	PF03150 PF02975 PF06433	20929212	oxidoreductase / electron transport	Paracoccus denitrificans PD1222	Yes	x-ray diffraction	1.91	Q51658 P22619 A1BB97	Kd	9.4e-06	B	E	25.0	7.2
3p9w	PPI4DOC	PF00341	23507309	signaling protein / immune system	Homo sapiens	No	x-ray diffraction	2.41	P15692	Kd	1.6e-08	A	B	25.0	7.2
3qc8	PDBind	PF02933 PF02359 PF00789	21739474	protein binding	Homo sapiens	No	x-ray diffraction	2.2	P55072 Q9UNN5	Kd	1.12e-05	0	1	25.0	7.2

PDB	Database origin	Protein family	PubMed	Classification	Organism	Mutation	Method	Res. (Å)	UniprotKB	Affinity type	Affinity (M)	Chain 1	Chain 2	Temp. (C)	pH
3qht	PPI4DOC	PF00240	21518904	de novo protein	Saccharomyces cerevisiae synthetic construct	No	x-ray diffraction	2.4	Q12306	Kd	8.2e-08	A	B	25.0	7.2
3rea	PPI4DOC	PF00469 PF00018	21625496	protein binding	HIV-1 M:B ARV2 / SF2 Homo sapiens	Yes	x-ray diffraction	2.0	P03407 P08631	Kd	1.79e-07	A	B	25.0	7.2
3ro2	PDBind	PF13181 PF13176 PF13374 PF13424	21816348	protein binding	Mus musculus Homo sapiens	No	x-ray diffraction	2.3	Q8VDU0 Q14980	Kd	4.6e-08	0	1	25.0	7.2
3rt0	PPI4DOC	PF00481 PF10604	21658606	hydrolase / hydrolase inhibitor	Arabidopsis thaliana	Yes	x-ray diffraction	2.11	Q9CAJ0 Q8H1R0	Kd	1.2e-06	A	B	25.0	7.2
3sgb	PDBind	PF00089 PF00050	6414511	complex(serine proteinase-inhibitor)	Streptomyces griseus Meleagris gallopavo	No	x-ray diffraction	1.8	P00777 P68390	Kd	1.79e-11	0	1	25.0	7.2
3tac	PDBind	PF00536 PF07647 PF00069	21855798	transferase / protein binding	Homo sapiens	No	x-ray diffraction	2.2	O14936 O75334	Kd	5.5e-07	0	1	25.0	7.2
3tei	PDBind	PF00069	23047924	transferase	Homo sapiens	Yes	x-ray diffraction	2.4	P28482 Q15418	Kd	5e-07	0	1	25.0	7.2
3tkl	PPI4DOC PDBind	PF00071	22416225	protein transport / protein binding	Homo sapiens Legionella pneumophila str. Corby	No	x-ray diffraction	2.18	P62820	Kd	4.3e-07	A	B	25.0	7.2
3tnf	PDBind	PF00071 PF18641	22011575	protein transport	Homo sapiens Legionella pneumophila subsp. pneumophila str. Philadelphia 1	No	x-ray diffraction	2.5	P61006 Q5ZWZ3	Kd	1e-11	0	1	25.0	7.2
3tu3	PDBind	PF01734 PF20848 PF20983	23166655	toxin / toxin chaperone	Pseudomonas aeruginosa	No	x-ray diffraction	1.92	O66100 O34208	Kd	5.7e-08	0	1	25.0	7.2
3tz1	PDBind	PF13499 PF00992	23096565	contractile protein	Chlamys nipponensis akazara	No	x-ray diffraction	1.8	Q27428 Q7M3Y3	Kd	1.41e-07	0	1	25.0	7.2
3u43	PDBind	PF01320 PF21431	22306467	protein binding	Escherichia coli	No	x-ray diffraction	1.72	P04482 P04419	Kd	1e-15	0	1	25.0	7.2
3ul4	PDBind	PF00963 PF00404	23118225	cell adhesion / protein binding	Acetivibrio thermocellus ATCC 27405	No	x-ray diffraction	1.95	Q06848 L7MTK2	Kd	1.14e-08	0	1	25.0	7.2
3uyp	PPI4DOC PDBind	PF02832	22285214	immune system	Mus musculus dengue virus type 4	No	x-ray diffraction	2.0	Q2YHF0	Kd	4.1e-06	A	B	25.0	7.2
3uzq	PPI4DOC PDBind	PF02832	22285214	immune system	Mus musculus dengue virus type 1	No	x-ray diffraction	1.6	P27909	Kd	8.2e-11	A	B	25.0	7.2
3uzv	PPI4DOC PDBind	PF00869 PF02832	22285214	immune system	Dengue virus 2 Jamaica / 1409 / 1983 Mus musculus	No	x-ray diffraction	2.1	P07564	Kd	4.3e-10	A	B	25.0	7.2
3w8i	PDBind	PF20929 PF06840 PF20929	23665169	protein binding / transferase	Homo sapiens	No	x-ray diffraction	2.4	Q9BUL8 Q9P289	Kd	2.15e-09	0	1	25.0	7.2

PDB	Database origin	Protein family	PubMed	Classification	Organism	Mutation	Method	Res. (Å)	UniprotKB	Affinity type	Affinity (M)	Chain 1	Chain 2	Temp. (C)	pH
3w9e	PPI4DOC	PF01537	24100313	viral protein / immune system	Human herpesvirus 2 strain HG52 Homo sapiens	No	x-ray diffraction	2.3	Q69467	IC50	3.6e-09	A	B	25.0	7.2
3wa5	PDBind		24100309	hydrolase	Pseudomonas aeruginosa PAO1	No	x-ray diffraction	1.9	Q9HYC5 Q9HYC4	Kd	2.81e-08	0	1	25.0	7.2
3wdg	PDBind	PF03167 PF06106	24150946	hydrolase / hydrolase inhibitor	Staphylococcus aureus subsp. aureus MRSA252 Staphylococcus aureus	No	x-ray diffraction	2.2	Q6GJ88 Q936H5	Kd	1.198e-09	0	1	25.0	7.2
3wqb	PDBind	PF00082 PF01483 PF18492	25784551	hydrolase / chaperone	Aeromonas sobria	Yes	x-ray diffraction	1.41	Q9L5A4 W5JXD7	Kd	1.8e-09	0	1	25.0	7.2
3wwn	PPI4DOC PDBind	PF00696 PF21344	25392000	metal binding protein / transferase	Thermus thermophilus HB27	No	x-ray diffraction	1.85	O50147 Q9ZND7	Kd	6.62e-06	A	B	25.0	7.2
3zet	PDBind	PF00814 PF00814	23471679	hydrolase	Salmonella enterica subsp. enterica serovar Typhimurium str. ST4 / 74	No	x-ray diffraction	2.31	E8X8J1 E8XBD7	Kd	2.9e-07	0	1	25.0	7.2
3zkq	PPI4DOC PDBind	PF00026	23695257	hydrolase / immune system	Homo sapiens Lama glama	No	x-ray diffraction	1.51	Q9Y5Z0	Kd	1.2e-09	A	D	25.0	7.2
3zo0	PPI4DOC	PF07654 PF00622 PF13765	18420815	immune system / ligase	Mus musculus	No	x-ray diffraction	1.99	P01863 Q62191	Kd	4.37e-07	A	B	25.0	7.2
3zu7	PPI4DOC PDBind	PF00069	22843676	transferase / de novo protein	Rattus norvegicus synthetic construct	No	x-ray diffraction	1.97	P63086	Kd	6.6e-09	A	B	25.0	7.2
3zwz	PDBind	PF02430	22737069	immune system	Plasmodium falciparum 3D7	No	x-ray diffraction	2.1	Q7KQK5 Q8IKV6	Kd	2.03e-08	0	1	25.0	7.2
4a1u	PDBind	PF02180 PF00452 PF08945	22040025	apoptosis	Homo sapiens synthetic construct	No	x-ray diffraction	1.54	Q07817 O43521	Ki	5.4e-07	0	1	25.0	7.2
4a49	PPI4DOC PDBind	PF00097 PF00179	22266821	ligase	Homo sapiens	Yes	x-ray diffraction	2.21	P22681 P62837	Kd	4.2e-05	A	B	25.0	7.2
4an7	PPI4DOC PDBind	PF00089 PF00197	23094997	hydrolase / hydrolase inhibitor	Sus scrofa Tamarindus indica	No	x-ray diffraction	2.23	P00761 F4ZZG4	Ki	3.2e-09	A	B	25.0	7.2
4apx	PPI4DOC PDBind	PF00028 PF00028 PF18432	23135401	cell adhesion	Mus musculus	No	x-ray diffraction	1.65	Q99PF4 Q99PJ1	Kd	2.9e-06	A	B	25.0	7.2
4awx	PDBind	PF09012	23024345	metal transport	Klebsiella pneumoniae	No	x-ray diffraction	2.3	B5XTS6	Kd	5e-07	0	1	25.0	7.2
4ayi	PPI4DOC	PF00084 PF08794 PF20937	23133374	immune system	Homo sapiens Neisseria meningitidis MC58	Yes	x-ray diffraction	2.31	P08603 Q19KF7	Kd	2e-09	A	D	25.0	7.2

PDB	Database origin	Protein family	PubMed	Classification	Organism	Mutation	Method	Res. (Å)	UniprotKB	Affinity type	Affinity (M)	Chain 1	Chain 2	Temp. (C)	pH
4b1x	PDBind	PF02755 PF00022	23041370	structural protein	Oryctolagus cuniculus Mus musculus	No	x-ray diffraction	1.8	P68135 Q2M3X8	Kd	4.34e-06	0	1	25.0	7.2
4b93	PDBind	PF13774 PF12796	23104059	exocytosis	Mus musculus Homo sapiens	No	x-ray diffraction	2.0	P70280 Q96NW4	Kd	2.3e-06	0	1	25.0	7.2
4bd9	PDBind	PF00246 PF00014	23746805	hydrolase / hydrolase inhibitor	Homo sapiens Sabellastarte magnifica	Yes	x-ray diffraction	2.2	Q9UI42 P84875	Ki	3.1e-08	0	1	25.0	7.2
4c2a	PDBind	PF00092 PF16164 PF13855 PF01462	24391089	blood clotting	Homo sapiens	Yes	x-ray diffraction	2.08	P04275 P07359	Kd	9.7e-09	0	1	25.0	7.2
4c4p	PDBind	PF00071 PF09457	24056041	protein transport	Homo sapiens	No	x-ray diffraction	2.0	P62491 Q7L804	Kd	2.5e-07	0	1	25.0	7.2
4c5g	PDBind	PF02820	24186981	transcription	Drosophila melanogaster	No	x-ray diffraction	2.1	Q9VK33 Q8ST83	Kd	1.03e-06	0	1	25.0	7.2
4c7n	PDBind	PF11851 PF00010	24631970	transcription	Homo sapiens synthetic construct	No	x-ray diffraction	2.1	O75030	Kd	1e-09	0	1	25.0	7.2
4c9b	PDBind	PF00270 PF00271 PF02854	24218557	splicing	Homo sapiens	No	x-ray diffraction	2.0	P38919 Q9HCG8	Kd	2.84e-08	0	1	25.0	7.2
4cj0	PPI4DOC PDBind	PF00759 PF02927	24823716	hydrolase / de novo protein	Acetivibrio thermocellus synthetic construct	No	x-ray diffraction	1.1	P0C2S4	Kd	9.8e-08	A	B	25.0	7.2
4cmm	PPI4DOC PDBind	PF07686 PF08204	24550402	signaling protein	Homo sapiens	Yes	x-ray diffraction	1.92	P78324 Q08722	Kd	8e-07	A	B	25.0	7.2
4ct0	PDBind	PF00875 PF03441 PF12114	24855952	circadian clock protein	Mus musculus	No	x-ray diffraction	2.45	P97784 O54943	Kd	2.8e-08	0	1	25.0	7.2
4d0g	PDBind	PF00071 PF09457	26032412	hydrolase	Homo sapiens	Yes	x-ray diffraction	2.5	P61106 Q6WKZ4	Kd	1.8e-06	0	1	25.0	7.2
4d0n	PPI4DOC PDBind	PF00071 PF00169 PF00621	25186459	cell cycle	Homo sapiens	No	x-ray diffraction	2.1	P61586 Q12802	Kd	1.81e-05	A	B	25.0	7.2
4drx	3DComplexV6	PF00091 PF03953 PF00091 PF03953	22778434	cell cycle	Ovis aries synthetic construct	No	x-ray diffraction	2.22	D0VWZ0 D0VWY9	Kd	1.55e-07	AB	F	25.0	7.2
4dt1	PDBind	PF16834 PF16836	22465956	dna binding protein	Saccharomyces cerevisiae S288C	No	x-ray diffraction	1.9	P40465 Q12318	Kd	5.6e-07	0	1	25.0	7.2
4dtg	PPI4DOC	PF00014	22563084	blood clotting inhibitor / immune system	Homo sapiens	No	x-ray diffraction	1.8	P10646	Kd	7.3e-11	HL	K	25.0	7.2
4dxa	PDBind	PF00071 PF00373	22577140	protein binding	Homo sapiens	Yes	x-ray diffraction	1.95	P61224 O00522	Kd	1.8e-06	0	1	25.0	7.2
4eig	PDBind	PF00186	23911607	oxidoreductase / immune system	Escherichia coli K-12 Lama glama	No	x-ray diffraction	2.5	P0ABQ4	Kd	1e-09	0	1	25.0	7.2

PDB	Database origin	Protein family	PubMed	Classification	Organism	Mutation	Method	Res. (Å)	UniprotKB	Affinity type	Affinity (M)	Chain 1	Chain 2	Temp. (C)	pH
4eoz	PPI4DOC	PF00651 PF00888	22632832	protein binding	Homo sapiens	Yes	x-ray diffraction	2.4	O43791 Q13618	Kd	1e-06	A	D	25.0	7.2
4etp	PDBind	PF00225 PF16796	22734002	motor protein	Saccharomyces cerevisiae S288C	Yes	x-ray diffraction	2.3	P17119 Q12045	Kd	3.7e-07	0	1	25.0	7.2
4etw	PPI4DOC	PF00561 PF00550	23045647	hydrolase	Shigella flexneri Shigella flexneri 5 str. 8401	Yes	x-ray diffraction	2.05	Q83PW0 Q0T5U2	Kd	3.1e-06	A	B	25.0	7.2
4euk	PDBind	PF00072 PF01627	23132142	signaling protein	Arabidopsis thaliana	No	x-ray diffraction	1.95	Q3S4A7 Q9ZNV9	Kd	4.1e-06	0	1	25.0	7.2
4fqi	BM5.5	PF00509 PF00509	22878502	viral protein / immune system	Influenza A virus (A / Viet Nam / 1203 / 2004(H5N1)) Homo sapiens	No	x-ray diffraction	1.71	Q6DQ33 Q6DQ33	Kd	9e-10	HL	AB	30.0	7.4
4fqj	PPI4DOC	PF00509	22878502	viral protein / immune system	Influenza B virus (B / reassortant / NYMC BX-21A(Lee / 1940 x Florida / 04 / 2006)) Homo sapiens	No	x-ray diffraction	2.5	I0B7N4	Kd	9e-10	HL	A	25.0	7.2
4fzv	PDBind	PF01189 PF02536	23022348	transferase	Homo sapiens	No	x-ray diffraction	2.0	Q96CB9 Q7Z6M4	Kd	1.33e-08	0	1	25.0	7.2
4g01	PDBind	PF18151 PF02204 PF00071	23519409	transport protein	Arabidopsis thaliana	No	x-ray diffraction	2.2	Q9LT31 Q9SN68	Kd	3.4e-06	0	1	25.0	7.2
4g35	PDBind	PF00452	22920569	apoptosis / inhibitor	Mus musculus	No	x-ray diffraction	2.0	P97287	Ki	5.4e-08	0	1	25.0	7.2
4g59	PPI4DOC	PF14586 PF11624	23169621	immune system	Mus musculus Murid betaherpesvirus 1	No	x-ray diffraction	2.44	O08604 Q83156	Kd	4.2e-07	A	B	25.0	7.2
4g6u	PDBind	PF21111 PF21483 PF07262	23236156	toxin	Escherichia coli O157:H7	Yes	x-ray diffraction	2.35	F2WK69 F2WK70	Kd	1.78e-08	0	1	25.0	7.2
4gaf	PDBind	PF00340 PF18452 PF13895	23431173	signaling protein	Homo sapiens	No	x-ray diffraction	2.15	P01584 P14778	Kd	1.8e-12	0	1	25.0	7.2
4gi3	PPI4DOC PDBind	PF00082	23075397	hydrolase / hydrolase inhibitor	Bacillus licheniformis Schistocerca gregaria	No	x-ray diffraction	1.75	Q9FDF2 P85064	Ki	6.8e-10	A	C	25.0	7.2
4h2w	PPI4DOC	PF00587 PF00550	23541895	ligase	Bradyrhizobium diazoefficiens USDA 110 Agrobacterium fabrum str. C58	No	x-ray diffraction	1.95	Q89VT8 A9CHM9	Kd	1.73e-06	A	D	25.0	7.2
4h5s	PPI4DOC PDBind	PF07686 PF07686	23871486	cell adhesion	Homo sapiens	No	x-ray diffraction	1.7	O95727 Q9BY67	Kd	1.25e-05	A	B	25.0	7.2

PDB	Database origin	Protein family	PubMed	Classification	Organism	Mutation	Method	Res. (Å)	UniprotKB	Affinity type	Affinity (M)	Chain 1	Chain 2	Temp. (C)	pH
4h6j	PDBind	PF08447 PF14598	23033253	transcription	Homo sapiens	Yes	x-ray diffraction	1.52	Q16665 P27540	Kd	1.9e-07	0	1	25.0	7.2
4hep	PDBind	PF18338 PF08931	23530214	viral protein	Lactococcus phage TP901-1 Lama glama	No	x-ray diffraction	1.75	Q9G096	Kd	3e-07	0	1	25.0	7.2
4hff	PDBind	PF14113 PF16695	23288853	hydrolase	Salmonella enterica subsp. enterica serovar Typhimurium str. LT2	No	x-ray diffraction	2.4	Q93IS4 Q8ZRL5	Kd	2.69e-10	0	1	25.0	7.2
4hfk	PPI4DOC	PF14113 PF16695	23288853	hydrolase	Enterobacter cloacae subsp. cloacae ATCC 13047	No	x-ray diffraction	2.1	A0A0H3CIJ2 A0A0H3CIX8	Kd	2.69e-10	C	D	25.0	7.2
4hgm	PPI4DOC PDBind	PF00273	23632026	immune system	Squalus acanthias Homo sapiens	No	x-ray diffraction	2.34	P02768	Kd	1.6e-08	B	A	25.0	7.2
4hjj	PPI4DOC	PF00340	23549062	immune system	Homo sapiens	Yes	x-ray diffraction	2.1	Q14116	Kd	1.6e-10	HL	A	25.0	7.2
4i2x	PPI4DOC	PF07686 PF07654	23826770	immune system	Homo sapiens	No	x-ray diffraction	2.48	Q9P1W8	Kd	1.2e-06	C	B	25.0	7.2
4i9x	PPI4DOC	PF16758 PF00020	23555243	apoptosis	Human herpesvirus 5 strain Merlin Homo sapiens	No	x-ray diffraction	2.1	Q6RJQ3 O14763	Kd	5.96e-09	B	D	25.0	7.2
4ilw	PPI4DOC	PF00965 PF00413	24073280	hydrolase / hydrolase inhibitor	Homo sapiens	No	x-ray diffraction	2.1	P16035 P09238	Ki	5.8e-09	A	B	25.0	7.2
4iu3	PDBind	PF18244	23580648	structural protein	Ruminococcus flavefaciens	No	x-ray diffraction	1.97	A0AEF6 A0AEF5	Kd	2.083e-08	0	1	25.0	7.2
4jeg	PPI4DOC PDBind	PF00017 PF00041	23980151	signaling protein / protein binding	Homo sapiens	No	x-ray diffraction	2.3	Q06124 P02751	Kd	1.2e-08	A	B	25.0	7.2
4jeh	PPI4DOC PDBind	PF00995 PF05739 PF00804	23858467	endocytosis / exocytosis	Rattus norvegicus	No	x-ray diffraction	2.5	P61765 P32851	Kd	8.1e-09	A	B	25.0	7.2
4jpk	PPI4DOC	PF00516	23539181	immune system / viral protein	Homo sapiens Human immunodeficiency virus 1	No	x-ray diffraction	2.4	P04578	Kd	4.4e-08	HL	A	25.0	7.2
4k2u	PPI4DOC	PF05424	23717209	immune system	Plasmodium falciparum 3D7 Mus musculus	No	x-ray diffraction	2.45	Q8IBE8	Kd	1.53e-11	A	B	25.0	7.2
4k5a	PPI4DOC PDBind	PF02180 PF00452	24747052	apoptosis	Bos taurus Escherichia coli	Yes	x-ray diffraction	1.5	Q1RMX3	Kd	1.03e-08	A	B	25.0	7.2
4k94	PPI4DOC		24127596	immune system	Homo sapiens	No	x-ray diffraction	2.4	P10721	Kd	6.3e-10	HL	C	25.0	7.2
4ka2	PDBind	PF00516	23710622	viral protein / inhibitor	Human immunodeficiency virus 1	No	x-ray diffraction	1.79	P35961	Kd	8.4e-12	0	1	25.0	7.2

PDB	Database origin	Protein family	PubMed	Classification	Organism	Mutation	Method	Res. (Å)	UniprotKB	Affinity type	Affinity (M)	Chain 1	Chain 2	Temp. (C)	pH
4kgq	PPI4DOC	PF00020 PF00229	25087510	immune system	Homo sapiens	Yes	x-ray diffraction	2.27	O95407 O43557	Kd	3.6e-08	C	B	25.0	7.2
4kt1	PPI4DOC	PF13855 PF01462 PF15913	23756652	hormone receptor / cell adhesion	Homo sapiens	No	x-ray diffraction	2.5	Q9BXB1 Q2MKA7	Kd	5.65e-08	A	E	25.0	7.2
4kt3	PDBind	PF01832 PF21576	23878199	hydrolase	Pseudomonas protegens Pf-5	No	x-ray diffraction	1.44	Q4KC90 Q4KC91	Kd	2.6e-10	0	1	25.0	7.2
4l0p	PPI4DOC	PF01404 PF00812	25993310	transferase / transferase receptor	Homo sapiens	No	x-ray diffraction	2.26	P29320 P52803	Kd	9e-09	A	B	25.0	7.2
4lad	PDBind	PF00179 PF13639	23942235	ligase / ligase	Homo sapiens	No	x-ray diffraction	2.3	P60604 Q9UKV5	Kd	3.8e-08	0	1	25.0	7.2
4lgr	PPI4DOC PDBind	PF00161	24907552	hydrolase / immune system	Ricinus communis Vicugna pacos	No	x-ray diffraction	1.65	P02879	Kd	1.1e-10	A	B	25.0	7.2
4llo	3DComplexV6	PF00027 PF13426	23975098	transport protein	Mus musculus	No	x-ray diffraction	2.0	Q60603 Q60603	Kd	1.32e-05	A	B	25.0	7.2
4lxr	PPI4DOC	PF13855 PF01462 PF00560 PF01463 PF16077	24733933	immune system / cytokine	Drosophila melanogaster	No	x-ray diffraction	2.2	P08953 P48607	Kd	5.9e-09	A	JK	25.0	7.2
4m0w	PPI4DOC PDBind	PF08715 PF00240	24531491	hydrolase / protein binding	Severe acute respiratory syndrome-related coronavirus Bos taurus	Yes	x-ray diffraction	1.4	P0C6U8 P62992	Kd	6.7e-06	A	B	25.0	7.2
4m1g	PPI4DOC	PF05966	26325270	immune system	Mus musculus Vaccinia virus	Yes	x-ray diffraction	1.6	Q71TT1	Kd	1.44e-08	HL	AB	25.0	7.2
4m5z	BM5.5	PF00509	24027321	viral protein / immune system	Influenza A virus Homo sapiens	No	x-ray diffraction	2.25	G8XMJ2	Kd	5e-09	HL	A	25.0	7.2
4m62	PPI4DOC	PF07686	25254371	immune system	Homo sapiens synthetic construct	No	x-ray diffraction	1.8	P01619	Kd	2.57e-09	HL	S	25.0	7.2
4mjs	PPI4DOC	PF00564 PF00564	24369353	transferase / protein binding	Rattus norvegicus Homo sapiens	Yes	x-ray diffraction	2.5	P09217 Q13501	Kd	2.16e-08	A	B	25.0	7.2
4mrt	3DComplexV6 PDBind	PF00550 PF01648	24704508	transport protein / transferase	Brevibacillus parabrevis Bacillus subtilis subsp. subtilis str. 168	Yes	x-ray diffraction	2.0	O30409 P39135	Kd	9.2e-07	0	1	25.0	7.2
4nl9	PPI4DOC	PF00536 PF00536	24998259	structural protein	Homo sapiens	Yes	x-ray diffraction	1.5	Q6ZW76 Q68DC2	Kd	2.49e-07	A	B	25.0	7.2
4nqw	PDBind	PF04545 PF10099	24699647	dna binding protein / protein binding	Mycobacterium tuberculosis	No	x-ray diffraction	2.4	P9WGH7 P9WGX5	Kd	4.2e-07	0	1	25.0	7.2

PDB	Database origin	Protein family	PubMed	Classification	Organism	Mutation	Method	Res. (Å)	UniprotKB	Affinity type	Affinity (M)	Chain 1	Chain 2	Temp. (C)	pH
4nso	PDBind	PF21277 PF20889	24751834	protein binding	Vibrio cholerae O1 biovar El Tor str. N16961	No	x-ray diffraction	2.4	Q9KN42 Q9KN41	Kd	9.8e-10	0	1	25.0	7.2
4nu1	PDBind	PF00069	24642411	transferase / peptide	Mus musculus Homo sapiens	No	x-ray diffraction	2.5	Q9WV60 O15169	Ki	6e-05	0	1	25.0	7.2
4p3y	PPI4DOC PDBind	PF00009 PF03143 PF03144 PF01323	24860094	translation / oxidoreductase	Escherichia coli BL21(DE3) Acinetobacter baumannii AYE	No	x-ray diffraction	2.15	P0CE47	Kd	1.46e-07	A	B	25.0	7.2
4pas	PDBind	PF18455	24778228	signaling protein	Homo sapiens	No	x-ray diffraction	1.62	Q9UBS5 O75899	Kd	1.266e-07	0	1	25.0	7.2
4pbv	PPI4DOC	PF13855 PF16920 PF00047 PF13927 PF07679	25385546	signaling protein	Gallus gallus	Yes	x-ray diffraction	2.5	Q91044 F1NWE3	Kd	2.4e-06	A	B	25.0	7.2
4pbz	PDBind	PF12265 PF00400	24920672	cell cycle	Homo sapiens	No	x-ray diffraction	2.15	Q09028 Q13330	Kd	5e-08	0	1	25.0	7.2
4per	PPI4DOC PDBind	PF13516 PF18779 PF00074	24941155	hydrolase / hydrolase inhibitor	Gallus gallus	No	x-ray diffraction	1.92	Q5ZIY8 P27043	Kd	1e-15	A	B	25.0	7.2
4pj2	PPI4DOC	PF16743 PF05497	25664745	hydrolase / hydrolase inhibitor	Aeromonas hydrophila subsp. hydrophila ATCC 7966 Meretrix lusoria	No	x-ray diffraction	1.24	A0KHJ5 P86383	Kd	4.7e-11	A	B	25.0	7.2
4pp8	PPI4DOC	PF00059 PF14586	11825567	immune system	Mus musculus	No	x-ray diffraction	1.95	O54709 O08603	Kd	4.86e-07	A	C	25.0	7.2
4pqt	PDBind	PF01398	24787148	hydrolase / transcription	Schizosaccharomyces pombe 972h- Homo sapiens	Yes	x-ray diffraction	2.05	Q9P371	Kd	1.1e-06	0	1	25.0	7.2
4pw9	PDBind	PF00174 PF03404 PF13442	26687009	oxidoreductase / electron transport	Sinorhizobium meliloti 1021	No	x-ray diffraction	2.49	Q92M24 Q92M25	Kd	1.35e-05	0	1	25.0	7.2
4qci	PPI4DOC	PF04692 PF00341	25707433	cytokine / cytokine receptor	Homo sapiens	No	x-ray diffraction	2.3	P01127	Kd	2.8e-11	A	C	25.0	7.2
4qd2	PPI4DOC 3DComplexV6	PF14200 PF05588 PF03505 PF17993 PF00028	24948737	cell adhesion	Clostridium botulinum A str. Hall Mus musculus	No	x-ray diffraction	2.4	A5HZZ6 A5HZZ5 A5HZZ4 P09803	Kd	2.7e-06	D	E	25.0	7.2
4qlp	PDBind	PF15598 PF14021	26237511	hydrolase / protein binding	Mycobacterium tuberculosis H37Rv	No	x-ray diffraction	1.1	O05443 O05442	Kd	2.3e-10	0	1	25.0	7.2
4qxa	PDBind	PF00071	25220469	protein transport / protein binding	Mus musculus	Yes	x-ray diffraction	2.3	Q9R0M6 Q8BPQ7	Kd	3.2e-07	0	1	25.0	7.2
4rey	PDBind	PF04495 PF19046	26363069	membrane protein	Homo sapiens	No	x-ray diffraction	1.96	Q9BQQ3 Q08379	Kd	1.08e-07	0	1	25.0	7.2

PDB	Database origin	Protein family	PubMed	Classification	Organism	Mutation	Method	Res. (Å)	UniprotKB	Affinity type	Affinity (M)	Chain 1	Chain 2	Temp. (C)	pH
4tq1	PDBind	PF20637 PF20638 PF04106	25484072	protein binding	Homo sapiens	No	x-ray diffraction	1.8	Q9H1Y0 Q7Z6L1	Kd	0.00035	0	1	25.0	7.2
4u32	PPI4DOC PDBind	PF00014 PF00089	25301953	hydrolase / hydrolase inhibitor	Homo sapiens	Yes	x-ray diffraction	1.65	O43291 P35030	Ki	3.79e-07	A	X	25.0	7.2
4u4c	PDBind	PF08148 PF00270 PF00271 PF21408 PF13234	25175027	hydrolase	Saccharomyces cerevisiae S288C	No	x-ray diffraction	2.4	P47047 P53632 Q12476	Kd	3.1e-07	0	1	25.0	7.2
4w4l	PPI4DOC	PF00934 PF00823 PF14011	25275011	protein transport	Mycobacterium tuberculosis str. Erdman = ATCC 35801	No	x-ray diffraction	2.45	A0A0H3LBR3 A0A0H3LBN6 A0A0H3LAM1	Kd	1.3e-09	C	B	25.0	7.2
4w6x	PDBind		25502211	cell adhesion	Escherichia coli Lama glama	No	x-ray diffraction	1.88	Q47212	Kd	5.25e-09	0	1	25.0	7.2
4wem	PPI4DOC PDBind	PF07686	25828907	structural protein	Escherichia coli Lama glama	No	x-ray diffraction	1.55	P14190 R9W2R6	Kd	6.8e-06	A	B	25.0	7.2
4wnd	PDBind	PF13181 PF13374 PF13176	25664792	signaling protein / protein binding	Homo sapiens	No	x-ray diffraction	1.5	P81274 Q14CM0	Kd	3.29e-08	0	1	25.0	7.2
4x33	PDBind	PF05207 PF13540	25604895	electron transport	Saccharomyces cerevisiae S288C	No	x-ray diffraction	1.45	Q3E840 P31386	Kd	2.5e-07	0	1	25.0	7.2
4x7f	PPI4DOC	PF00915 PF08435	25520510	viral protein	Norwalk virus Vicugna pacos	No	x-ray diffraction	1.7	Q5F4T5	Kd	2.8e-08	A	C	25.0	7.2
4x7s	PDBind		25849503	immune system	Homo sapiens	No	x-ray diffraction	1.9		Kd	1e-08	0	1	25.0	7.2
4xl1	PPI4DOC	PF00008 PF12661 PF07645 PF01414 PF07657	25700513	protein binding	Rattus norvegicus	Yes	x-ray diffraction	2.3	Q07008 D3ZHH1	Kd	3.04e-07	A	B	25.0	7.2
4xwj	PDBind	PF04353 PF00381	26457424	transcription / transferase	Escherichia coli K-12	Yes	x-ray diffraction	2.1	P0AFX4 P0AA04	Kd	4.45e-09	0	1	25.0	7.2
4xxb	PDBind	PF00281 PF00673 PF00641	26220995	rna binding protein / metal binding protein	Homo sapiens	No	x-ray diffraction	2.4	P62913 Q00987	Kd	1.48e-06	0	1	25.0	7.2
4y5o	PDBind	PF16545 PF00564	26235885	transferase	Homo sapiens	No	x-ray diffraction	2.35	Q9BSQ5 Q99759	Kd	1.4e-06	0	1	25.0	7.2
4yl8	PDBind	PF09379 PF09380 PF00373	25792740	protein binding	Mus musculus Drosophila melanogaster	No	x-ray diffraction	1.5	P26041 P10040	Kd	5.4e-06	0	1	25.0	7.2
4yn0	PPI4DOC PDBind	PF00020 PF12925	25838500	apoptosis / cell adhesion	Mus musculus	No	x-ray diffraction	2.2	Q9EPU5 P12023	Kd	8.5e-08	A	B	25.0	7.2
4yvq	PDBind	PF00745 PF13424	26037924	oxidoreductase / fluorescent protein	Arabidopsis thaliana	No	x-ray diffraction	2.4	P42804 Q940U6	Kd	2.03e-06	0	1	25.0	7.2

PDB	Database origin	Protein family	PubMed	Classification	Organism	Mutation	Method	Res. (Å)	UniprotKB	Affinity type	Affinity (M)	Chain 1	Chain 2	Temp. (C)	pH
4zii	PDBind	PF00452 PF06393	26158515	apoptosis	Homo sapiens	Yes	x-ray diffraction	2.19	Q07812 P55957	IC50	6.6e-08	0	1	25.0	7.2
4zqu	PDBind	PF21111 PF07262	26449640	toxin	Yersinia pseudotuberculosis YPIII Salmonella enterica subsp. enterica serovar Rubislaw str. ATCC 10717	No	x-ray diffraction	2.09	A0A0H3B0B8 A0A0R4I987	Kd	1.6e-08	0	1	25.0	7.2
4zw2	PDBind	PF00625 PF12052	28351836	metal transport	Mus musculus	Yes	x-ray diffraction	1.86	Q8R3Z5 Q02789	Kd	4.9e-09	0	1	25.0	7.2
5b76	PDBind	PF00628 PF00125	27775714	transferase	Homo sapiens	Yes	x-ray diffraction	1.65	Q92794 K7EMV3	Kd	5.8e-06	0	1	25.0	7.2
5cxb	PDBind	PF08154 PF00400 PF08145 PF00400	26476442	protein binding	Thermochaetoides thermophila	No	x-ray diffraction	2.1	G0SFB5 G0SCK6	Kd	9.19e-09	0	1	25.0	7.2
5dfw	PDBind	PF00335	26637054	cell adhesion	Homo sapiens Mus musculus	No	x-ray diffraction	2.33	P60033	Kd	5e-10	0	1	25.0	7.2
5djt	PDBind	PF13426	27427858	signaling protein	Avena sativa Staphylococcus aureus	No	x-ray diffraction	1.4	O49004	Kd	1.7e-08	0	1	25.0	7.2
5dob	PDBind	PF02718 PF04541	26511021	dna binding protein	Human herpesvirus 5 strain AD169	Yes	x-ray diffraction	2.47	P16794 P16791	Kd	1e-06	0	1	25.0	7.2
5ee5	PDBind	PF16213 PF00025	27373159	transcription	Homo sapiens	No	x-ray diffraction	2.28	Q9Y6D6 P40616	Kd	2.6e-05	0	1	25.0	7.2
5elu	PDBind	PF11976	29138295	signaling protein	synthetic construct Homo sapiens	No	x-ray diffraction	2.35	P61956	Kd	4.14e-07	0	1	25.0	7.2
5eo9	PDBind	PF07686 PF13927 PF13927 PF00047	26687361	cell adhesion	Drosophila melanogaster	No	x-ray diffraction	2.3	M9PC40 Q9W4R3	Kd	3.7e-07	0	1	25.0	7.2
5f4e	PDBind	PF16706 PF15005 PF03024	27309818	cell adhesion	Homo sapiens	No	x-ray diffraction	2.4	Q8IYV9 A6ND01	Kd	4.8e-08	0	1	25.0	7.2
5f5s	PDBind	PF03371 PF06991	27773687	splicing	Homo sapiens	No	x-ray diffraction	2.4	Q8NAV1 P55081	Kd	2.2e-08	0	1	25.0	7.2
5g1x	PDBind	PF00069 PF01056	27837025	transferase	Homo sapiens	Yes	x-ray diffraction	1.72	O14965 P04198	Kd	1.21e-05	0	1	25.0	7.2
5gjk	PDBind	PF04433 PF04855	28438634	transcription	Homo sapiens	No	x-ray diffraction	2.05	Q92922 Q12824	Kd	1.2e-07	0	1	25.0	7.2
5gpg	PDBind	PF00254 PF08771	27610411	isomerase / transferase	Homo sapiens	No	x-ray diffraction	1.67	Q00688 P42345	IC50	2.61e-09	0	1	25.0	7.2

PDB	Database origin	Protein family	PubMed	Classification	Organism	Mutation	Method	Res. (Å)	UniprotKB	Affinity type	Affinity (M)	Chain 1	Chain 2	Temp. (C)	pH
5h3j	PDBind	PF04495	28049725	protein transport	Mus musculus	No	x-ray diffraction	1.33	Q99JX3 Q8R2X8	Kd	2.7e-07	0	1	25.0	7.2
5h7y	PDBind	PF18443 PF18426	28979890	hydrolase inhibitor / peptide	Pseudomonas aeruginosa PAO1	No	x-ray diffraction	2.19	Q9I3K3 Q9I3K2	Kd	1.25e-07	0	1	25.0	7.2
5hgg	BM5.5	PF00089	27226628	hydrolase / inhibitor	Homo sapiens Vicugna pacos	Yes	x-ray diffraction	1.97	P00749	Kd	5.4e-11	T	A	25.0	7.2
5hpk	PDBind	PF00632 PF00240	26949039	ligase	Homo sapiens	No	x-ray diffraction	2.43	Q96PU5 P62987	Kd	9.7e-09	0	1	25.0	7.2
5hu3	PDBind	PF00069	30381148	transferase	Drosophila melanogaster	Yes	x-ray diffraction	1.89	Q00168 Q02280	Kd	1e-06	0	1	25.0	7.2
5hys	BM5.5	PF07654	27194387	immune system	Homo sapiens	Yes	x-ray diffraction	2.5	P01854	Kd	3.89e-11	CD	JK	25.0	7.2
5imk	PDBind	PF07686	27889311	immune system	Camelidae Homo sapiens	No	x-ray diffraction	1.23	Q9Y279	Kd	8.5e-07	0	1	25.0	7.2
5imm	PDBind	PF07686	27889311	immune system	Camelidae Mus musculus	No	x-ray diffraction	1.2	F6TUL9	Kd	3.5e-09	0	1	25.0	7.2
5inb	PDBind	PF00149 PF16891 PF15276	27572260	hydrolase / protein binding	Homo sapiens	No	x-ray diffraction	1.3	P36873 Q69YH5	Kd	1.23e-07	0	1	25.0	7.2
5jds	PDBind	PF07686	28280600	immune system	Camelidae Homo sapiens	No	x-ray diffraction	1.7	Q9NZQ7	Kd	3e-09	0	1	25.0	7.2
5jjd	PDBind	PF00169 PF01852	28652409	lipid transport	Homo sapiens	No	x-ray diffraction	2.4	Q9Y5P4 Q9Y5P4	Kd	9.2e-06	0	1	25.0	7.2
5jw9	PDBind	PF05110 PF07303	28134250	protein binding	Homo sapiens	Yes	x-ray diffraction	2.0	Q9UHB7 O00472	Kd	8.6e-08	0	1	25.0	7.2
5kve	PDBind	PF02832	27475895	viral protein / immune system	Zika virus Mus musculus	No	x-ray diffraction	1.7	A0A024B7W	Kd	3.5e-08	0	1	25.0	7.2
5kxh	PDBind	PF10250 PF00008	28530709	transferase	Mus musculus	No	x-ray diffraction	1.33	Q91ZW2 P70375	Kd	4.9e-07	0	1	25.0	7.2
5l21	PDBind	PF07951 PF07953	28785006	toxin	Clostridium botulinum A str. Hall Vicugna pacos	Yes	x-ray diffraction	1.68	P0DPI1	Kd	3.7e-09	0	1	25.0	7.2
5li1	PDBind	PF00069 PF00433	27554858	transferase	Homo sapiens Xenopus tropicalis	No	x-ray diffraction	2.0	P41743 Q28E03	Kd	4.7e-07	0	1	25.0	7.2
5ma6	PDBind	PF01353	29176615	fluorescent protein	Aequorea victoria synthetic construct	Yes	x-ray diffraction	2.3	P42212	Kd	3.03e-10	0	1	25.0	7.2
5me5	PDBind	PF01652	28522457	translation	Cucumis melo	No	x-ray diffraction	1.9	Q00LS8 A0A1S3C4H6	Kd	2.15e-06	0	1	25.0	7.2

PDB	Database origin	Protein family	PubMed	Classification	Organism	Mutation	Method	Res. (Å)	UniprotKB	Affinity type	Affinity (M)	Chain 1	Chain 2	Temp. (C)	pH
5ml9	PDBind	PF13895	29247053	immune system	Homo sapiens synthetic construct	Yes	x-ray diffraction	2.35	P08637	Kd	2.17e-07	0	1	25.0	7.2
5mtj	PDBind	PF00017	28347651	signaling protein	Mus musculus Homo sapiens	No	x-ray diffraction	1.95	Q04736	Kd	3.38e-07	0	1	25.0	7.2
5mtm	PDBind	PF00017	28347651	transferase	Homo sapiens Mus musculus	No	x-ray diffraction	2.4	P06239	Kd	7e-09	0	1	25.0	7.2
5nqg	PDBind	PF02430	28817634	cell invasion	Plasmodium vivax Sal-1 Plasmodium vivax	Yes	x-ray diffraction	2.15	A5K4Z2 A5K3N8	Kd	5e-08	0	1	25.0	7.2
5nus	PDBind	PF03850	28977422	transcription	Thermochaetoides thermophila DSM 1495	No	x-ray diffraction	2.2	G0RXV8 G0RZE6	Kd	1.1e-08	0	1	25.0	7.2
5o90	PDBind	PF00069	29229647	transferase	Mus musculus Homo sapiens	Yes	x-ray diffraction	2.49	P47811 Q15750	Kd	1.1e-06	0	1	25.0	7.2
5oaq	PDBind	PF17725	28960584	transcription	Homo sapiens	No	x-ray diffraction	1.95	Q15561 P46937	Kd	5.8e-08	0	1	25.0	7.2
5oyl	PDBind	PF00974 PF00057	29531262	viral protein	Recombinant vesicular stomatitis Indiana virus rVSV-G / GFP Homo sapiens	No	x-ray diffraction	2.25	B7UCZ5 P01130	Kd	7.5e-06	0	1	25.0	7.2
5szh	PDBind	PF12130 PF00071	27552051	endocytosis	Homo sapiens	No	x-ray diffraction	2.3	O94851 Q9H0U4	Kd	5.2e-06	0	1	25.0	7.2
5t0f	PDBind	PF14215	28137867	transcription	Arabidopsis thaliana	No	x-ray diffraction	2.4	Q9FIP9 Q93ZM9	IC50	5.8e-08	0	1	25.0	7.2
5tar	PDBind	PF00071 PF05351	27791178	oncoprotein	Homo sapiens	No	x-ray diffraction	1.9	P01116 O43924	Kd	2.3e-06	0	1	25.0	7.2
5tvq	PDBind	PF03372 PF11976	28912134	hydrolase	Mus musculus	No	x-ray diffraction	2.35	Q9JJX7 P61957	Kd	8.8e-07	0	1	25.0	7.2
5tzp	PDBind	PF00452 PF12201	28411240	apoptosis	Fowlpox virus strain NVSL Gallus gallus	No	x-ray diffraction	1.35	Q9J5G4	Kd	3e-08	0	1	25.0	7.2
5un7	PDBind	PF21375	28393830	protein binding	Homo sapiens	No	x-ray diffraction	2.1	Q9NUX5 Q96AP0	Kd	1.2e-07	0	1	25.0	7.2
5uul	PDBind	PF00452 PF15826	28594323	apoptosis	Homo sapiens	Yes	x-ray diffraction	1.33	Q16548 Q9BXH1	Ki	4.8e-09	0	1	25.0	7.2
5vko	PDBind	PF00017	28826505	transcription	Saccharomyces cerevisiae S288C	No	x-ray diffraction	1.8	P23615 P04050	Kd	4e-09	0	1	25.0	7.2
5vmo	PDBind	PF00452 PF08945	29483196	viral protein / apoptosis	Grouper iridovirus Danio rerio	No	x-ray diffraction	1.7	Q5GAF0 B8JK68	Kd	8.87e-07	0	1	25.0	7.2
5vz4	PDBind	PF00019 PF02351	28953886	signaling protein / protein binding	Homo sapiens	No	x-ray diffraction	2.2	Q99988 Q6UXV0	Kd	8e-09	0	1	25.0	7.2

PDB	Database origin	Protein family	PubMed	Classification	Organism	Mutation	Method	Res. (Å)	UniprotKB	Affinity type	Affinity (M)	Chain 1	Chain 2	Temp. (C)	pH
5w89	PDBind	PF00452	29339518	peptide binding protein	Homo sapiens	No	x-ray diffraction	1.42	Q07820	IC50	2.5e-08	0	1	25.0	7.2
5wgg	PDBind	PF04055 PF13186 PF13353 PF13165	28704043	peptide binding protein	Acetivibrio thermocellus ATCC 27405	No	x-ray diffraction	2.04	A3DDW1 A3DDW2	Kd	7e-07	0	1	25.0	7.2
5wpa	PDBind	PF08075 PF00076 PF08075 PF00076	29530979	nuclear protein	Homo sapiens	No	x-ray diffraction	2.29	P23246 Q8WXF1	Kd	5.5e-07	0	1	25.0	7.2
5wrv	PDBind	PF16969 PF17004	28369529	protein transport	Homo sapiens	Yes	x-ray diffraction	1.7	Q9UHB9 O76094	Kd	7.3e-07	0	1	25.0	7.2
5wuj	PDBind	PF14842	29229777	motor protein	Helicobacter pylori 26695	No	x-ray diffraction	2.3	O25118 O25119	Kd	4.22e-07	0	1	25.0	7.2
5xiu	PDBind	PF00240	29330428	transferase / ribosomal protein	Homo sapiens Mus musculus	No	x-ray diffraction	1.8	Q8IYW5 P62983	Kd	2.5e-05	0	1	25.0	7.2
5xln	PDBind	PF01652	30902983	rna binding protein / ligase	Homo sapiens	No	x-ray diffraction	1.9	O60573 P26639	Kd	2.48e-06	0	1	25.0	7.2
5xoc	PDBind	PF03166 PF00085	29588413	transcription	Homo sapiens Escherichia coli K-12	No	x-ray diffraction	2.4	P84022 P0AA25 O75593	Kd	8e-06	0	1	25.0	7.2
5xod	PDBind	PF03166	29588413	transcription	Homo sapiens	No	x-ray diffraction	1.85	Q15796 P12755	Kd	2.81e-08	0	1	25.0	7.2
5y9j	BM5.5	PF00229	29572471	protein binding	Homo sapiens	No	x-ray diffraction	2.05	Q9Y275	Kd	9.95e-10	HL	A	25.0	7.2
5yi8	PDBind	PF00640	29467404	cell cycle	Drosophila melanogaster	No	x-ray diffraction	2.0	P16554 Q9W4I7	Kd	1.8e-06	0	1	25.0	7.2
5yip	PDBind	PF02991	29867141	signaling protein	Mus musculus Rattus norvegicus	No	x-ray diffraction	1.85	Q8R3R8 O70511	Kd	3.7e-09	0	1	25.0	7.2
5yr0	PDBind	PF17675 PF10186	29866835	endocytosis	Mus musculus	Yes	x-ray diffraction	1.9	O88597 Q8K245	Kd	7.6e-06	0	1	25.0	7.2
5ywr	PDBind	PF00179 PF13639	29626159	signaling protein	Homo sapiens	No	x-ray diffraction	1.47	P61088 Q8ND25	Kd	3.9e-08	0	1	25.0	7.2
6a0z	BM5.5	PF00509	29925655	viral protein / immune system	Influenza A virus (A / chicken / Chachoengsao / Thailand / CU-11 / 04(H5N1)) Human immunodeficiency virus 1 Mus musculus	No	x-ray diffraction	2.33	Q6DQ34 M1E1E4	Kd	5.3e-08	HL	A	25.0	7.2
6aaf	PDBind	PF02991	30451685	membrane protein	Schizosaccharomyces pombe 972h-	No	x-ray diffraction	2.2	O94272 Q09906	Kd	1.61e-07	0	1	25.0	7.2

PDB	Database origin	Protein family	PubMed	Classification	Organism	Mutation	Method	Res. (Å)	UniprotKB	Affinity type	Affinity (M)	Chain 1	Chain 2	Temp. (C)	pH
6akm	PDBind	PF05769	30622739	protein binding	Homo sapiens	No	x-ray diffraction	2.3	Q9BRV8 Q14BN4	Kd	6.9e-06	0	1	25.0	7.2
6b0s	BM5.5	PF00090	29167197	immune system	Homo sapiens Plasmodium falciparum	No	x-ray diffraction	1.95	P19597	Kd	1.78e-07	HL	C	25.0	7.2
6b6u	PDBind	PF00224 PF02887	29182273	transferase	Homo sapiens	Yes	x-ray diffraction	1.35	P14618	Kd	2.01e-05	0	1	25.0	7.2
6bmt	PDBind	PF03098	29524428	oxidoreductase / inhibitor	Homo sapiens Staphylococcus delphini	No	x-ray diffraction	2.4	P05164 A0A2A4GXB5	Kd	3.1e-07	0	1	25.0	7.2
6bw9	PDBind	PF00514 PF01749 PF16186	30209309	transport protein	Homo sapiens Hendra virus horse / Australia / Hendra / 1994	No	x-ray diffraction	1.6	O00629 P0C1C6	Kd	4.39e-09	0	1	25.0	7.2
6d4p	PDBind	PF00179 PF00240	31634471	transferase	Homo sapiens	Yes	x-ray diffraction	2.11	P51668 Q59EM9	IC50	6.5e-08	0	1	25.0	7.2
6dgf	PDBind	PF00443 PF00240	30763569	protein binding	Homo sapiens	Yes	x-ray diffraction	2.34	O75604 P0CG47	IC50	9.5e-09	0	1	25.0	7.2
6f0f	PDBind	PF04729	31543461	chaperone	Homo sapiens	No	x-ray diffraction	2.0	Q9Y294	Kd	1.8e-07	0	1	25.0	7.2
6fbx	PDBind	PF00452 PF10514	30237469	apoptosis	Danio rerio	No	x-ray diffraction	1.64	Q8UWD5 Q4V925	Kd	3.43e-07	0	1	25.0	7.2
6fc3	PDBind	PF01652 PF17052	30053226	translation	Saccharomyces cerevisiae S288C	Yes	x-ray diffraction	1.75	P07260 P12962	Kd	2e-08	0	1	25.0	7.2
6fp7	PDBind	PF01353	30237292	de novo protein	Clavularia sp. synthetic construct	Yes	x-ray diffraction	1.58	Q9U6Y3	Kd	3e-09	0	1	25.0	7.2
6fub	PDBind		29988155	antifungal protein	Pyricularia oryzae Oryza sativa Japonica Group	No	x-ray diffraction	1.3	C4B8C2 B5UBC1	Kd	2.9e-08	0	1	25.0	7.2
6fv0	PDBind	PF21376 PF00515 PF13374 PF13424	30320553	motor protein	Mus musculus Lama glama	No	x-ray diffraction	2.29	O88447 Q9ER39	Kd	1.72e-06	0	1	25.0	7.2
6gho	PDBind	PF03960 PF13743	30982633	protein binding	Bacillus subtilis subsp. subtilis str. 168 Geobacillus kaustophilus HTA426	No	x-ray diffraction	1.79	O31602 Q5L1S1	Kd	8e-07	0	1	25.0	7.2
6gum	PDBind	PF00179	31292170	transferase	Arabidopsis thaliana	No	x-ray diffraction	1.79	Q42551 Q9SJT1	Kd	1.2e-06	0	1	25.0	7.2
6har	PDBind	PF00089 PF00014	30700553	protein fibril	Homo sapiens	Yes	x-ray diffraction	1.5	Q8N2U3 H7C0V9	Ki	6.1e-11	0	1	25.0	7.2

PDB	Database origin	Protein family	PubMed	Classification	Organism	Mutation	Method	Res. (Å)	UniprotKB	Affinity type	Affinity (M)	Chain 1	Chain 2	Temp. (C)	pH
6her	PDBind	PF00377	31815959	protein binding	Mus musculus Camelus dromedarius	No	x-ray diffraction	1.2	P04925	Kd	4e-08	0	1	25.0	7.2
6idx	PDBind	PF11841 PF04727	30604775	cell adhesion	Homo sapiens Mus musculus	No	x-ray diffraction	1.7	Q96JJ3 Q3UHD1	Kd	3.36e-06	0	1	25.0	7.2
6imf	PDBind	PF08562 PF00188 PF05825	30504218	toxin / antitoxin	Protobothrops flavoviridis	No	x-ray diffraction	2.3	Q8JI39 A7VN14	Kd	2.4e-08	0	1	25.0	7.2
6isc	PDBind	PF07686 PF07686	30591568	immune system	Mus musculus Homo sapiens	No	x-ray diffraction	2.2	Q8K4F0 P15151	Kd	2.4e-06	0	1	25.0	7.2
6iwd	PDBind	PF00102 PF00527	31323018	oncoprotein	Homo sapiens human papillomavirus 18	No	x-ray diffraction	1.8	Q15678 P06788	Kd	1.82e-08	0	1	25.0	7.2
6j4o	PDBind	PF14822 PF15674	31235911	hydrolase	Homo sapiens	No	x-ray diffraction	2.3	Q86V25 Q8N300	Kd	1.8e-08	0	1	25.0	7.2
6jwj	PDBind	PF05020 PF05021	31836717	protein binding	Saccharomyces cerevisiae S288C	Yes	x-ray diffraction	1.58	P33755 P53044	Kd	8.57e-08	0	1	25.0	7.2
6kbr	PDBind	PF00089 PF00050	31391482	hydrolase / hydrolase inhibitor	Homo sapiens	No	x-ray diffraction	2.0	Q9Y5K2 P20155	Kd	8.91e-12	0	1	25.0	7.2
6mav	PDBind	PF00413 PF00965	31040180	hydrolase / hydrolase inhibitor	Homo sapiens	Yes	x-ray diffraction	2.37	P08254 P01033	Ki	3.35e-11	0	1	25.0	7.2
6ne4	PDBind	PF01392	31086346	biosynthetic protein / signaling protein	Escherichia coli Homo sapiens	No	x-ray diffraction	1.65	O75084	Kd	1.6e-09	0	1	25.0	7.2
6pnp	PDBind	PF02210 PF06312	31566781	cell adhesion	Mus musculus Rattus norvegicus	Yes	x-ray diffraction	1.94	Q9CS84 Q63366	Kd	2.992e-07	0	1	25.0	7.2
6r2g	PDBind	PF00517 PF00517	31255705	biosynthetic protein	Human immunodeficiency virus 1 Human immunodeficiency virus type 1 (BRU ISOLATE)	Yes	x-ray diffraction	1.9	P03377 P03377	Kd	7.58e-13	0	1	25.0	7.2
6umt	PDBind	PF07686	31727844	immune system	Homo sapiens	Yes	x-ray diffraction	1.99	Q15116 Q9BQ51	Kd	2.6e-09	0	1	25.0	7.2

References

[1] H. M. Berman. "The Protein Data Bank". In: *Nucleic Acids Research* 28.1 (2000), pp. 235–242. ISSN: 13624962. DOI: 10.1093/nar/28.1.235.

[2] Naomi E Chayen and Emmanuel Saridakis. "Protein crystallization: from purified protein to diffraction-quality crystal". In: *Nature Methods* 5.2 (2008), pp. 147–153. ISSN: 1548-7105. DOI: 10.1038/nmeth.f.203.

[3] Francesca Magnani et al. "A mutagenesis and screening strategy to generate optimally thermostabilized membrane proteins for structural studies". In: *Nature Protocols* 11.8 (2016), pp. 1554–1571. ISSN: 1750-2799. DOI: 10.1038/nprot.2016.088.

[4] Andrew Shrake and John A Rupley. "Environment and exposure to solvent of protein atoms. Lysozyme and insulin". In: *Journal of molecular biology* 79.2 (1973), pp. 351–371.

[5] Robert T. McGibbon et al. "MDTraj: A Modern Open Library for the Analysis of Molecular Dynamics Trajectories". In: *Biophysical Journal* 109.8 (2015), pp. 1528–1532. DOI: 10.1016/j.bpj.2015.08.015.

[6] Volkhard Helms and Olga V. Kalinina, eds. *Protein interactions. The molecular basis of interactomics.* Includes index. Weinheim, Germany: Wiley-VCH, 2023. 1 p. ISBN: 3527830502.

[7] Elizabeth Jurrus et al. "Improvements to the APBS biomolecular solvation software suite". In: *Protein Science* 27.1 (2018), pp. 112–128. ISSN: 0961-8368, 1469-896X. DOI: 10.1002/pro.3280.

[8] Chresten R. Søndergaard et al. "Improved treatment of ligands and coupling effects in empirical calculation and rationalization of p k $_a$ values". In: *Journal of Chemical Theory and Computation* 7.7 (2011), pp. 2284–2295. ISSN: 1549-9618, 1549-9626. DOI: 10.1021/ct200133y.

[9] Mats H. M. Olsson et al. "Propka3: consistent treatment of internal and surface residues in empirical p k $_a$ predictions". In: *Journal of Chemical Theory and Computation* 7.2 (2011), pp. 525–537. ISSN: 1549-9618, 1549-9626. DOI: 10.1021/ct100578z.

[10] Peter Eastman et al. "OpenMM 7: Rapid development of high performance algorithms for molecular dynamics". In: *PLOS Computational Biology* 13.7 (2017). Ed. by Robert Gentleman, e1005659. ISSN: 1553-7358. DOI: 10.1371/journal.pcbi.1005659.

[11] James A. Maier et al. "ff14sb: improving the accuracy of protein side chain and backbone parameters from ff99sb". In: *Journal of Chemical Theory and Computation* 11.8 (2015), pp. 3696–3713. ISSN: 1549-9618, 1549-9626. DOI: 10.1021/acs.jctc.5b00255.

[12] William Humphrey, Andrew Dalke, and Klaus Schulten. "VMD: Visual molecular dynamics". In: *Journal of Molecular Graphics* 14.1 (1996), pp. 33–38. ISSN: 0263-7855. DOI: 10.1016/0263-7855(96)00018-5.

[13] W. M. Haynes, David R. Lide, and Thomas J. Bruno, eds. *CRC Handbook of Chemistry and Physics.* 97th ed. CRC Press, 2016. ISBN: 9781315380476. DOI: 10.1201/9781315380476. (Visited on 05/14/2025).

[14] Emmanuel D. Levy. "A simple definition of structural regions in proteins and its use in analyzing interface evolution". In: *Journal of Molecular Biology* 403.4 (2010), pp. 660–670. ISSN: 00222836. DOI: 10.1016/j.jmb.2010.09.028.

[15] Christelle Pommié et al. "IMGT standardized criteria for statistical analysis of immunoglobulin V-REGION amino acid properties". In: *Journal of Molecular Recognition* 17.1 (2004), pp. 17–32. ISSN: 1099-1352. DOI: 10.1002/jmr.647.

[16] Katarina Elez, Alexandre M. J. J. Bonvin, and Anna Vangone. "Distinguishing crystallographic from biological interfaces in protein complexes: role of intermolecular contacts and energetics for classification". In: *BMC Bioinformatics* 19.S15 (2018). ISSN: 1471-2105. DOI: 10.1186/s12859-018-2414-9.

[17] Koen M. Visscher, Panagiotis L. Kastritis, and Alexandre M. J. J. Bonvin. "Non-interacting surface solvation and dynamics in protein-protein interactions: Surface Solvation Effects in Protein Binding". In: *Proteins: Structure, Function, and Bioinformatics* 83.3 (2015), pp. 445–458. ISSN: 0887-3585. DOI: 10.1002/prot.24741.

[18] Anna Vangone and Alexandre MJJ Bonvin. "Contacts-based prediction of binding affinity in protein-protein complexes". In: *eLife* 4 (July 2015), e07454. ISSN: 2050-084X. DOI: 10.7554/elife.07454.

[19] Greta Grassmann et al. "Electrostatic complementarity at the interface drives transient protein-protein interactions". In: *Scientific Reports* 13.1 (2023), p. 10207. ISSN: 2045-2322. DOI: 10.1038/s41598-023-37130-z.

[20] Edwin B. Wilson. "Probable Inference, the Law of Succession, and Statistical Inference". In: *Journal of the American Statistical Association* 22.158 (1927), pp. 209–212. ISSN: 0162-1459, 1537-274X. DOI: 10.1080/01621459.1927.10502953. (Visited on 03/09/2025).

[21] C. E. Shannon. "A Mathematical Theory of Communication". In: *Bell System Technical Journal* 27.3 (1948), pp. 379–423. ISSN: 00058580. DOI: 10.1002/j.1538-7305.1948.tb01338.x. (Visited on 09/18/2024).

[22] E.C. Pielou. "The measurement of diversity in different types of biological collections". In: *Journal of Theoretical Biology* 13 (1966), pp. 131–144. ISSN: 00225193. DOI: 10.1016/0022-5193(66)90013-0.

[23] H. P. Luhn. "A Statistical Approach to Mechanized Encoding and Searching of Literary Information". In: *IBM Journal of Research and Development* 1.4 (1957), pp. 309–317. ISSN: 0018-8646, 0018-8646. DOI: 10.1147/rd.14.0309. (Visited on 09/18/2024).

[24] Karen Sparck Jones. "A statistical interpretation of term specificity and its application in retrieval". In: *Journal of Documentation* 28.1 (1972), pp. 11–21. ISSN: 0022-0418. DOI: 10.1108/eb026526.

[25] Robert M. Fano. *Transmission of Information A Statistical Theory of Communication*. The MIT Press, 1961.

[26] Kenneth Ward Church and Patrick Hanks. "Word association norms, mutual information, and lexicography". In: *Proceedings of the 27th annual meeting on Association for Computational Linguistics*. Association for Computational Linguistics, 1989, pp. 76–83. DOI: 10.3115/981623.981633.

[27] Kenneth Ward Church and Patrick Hanks. "Word Association Norms, Mutual Information, and Lexicography". In: *Computational Linguistics* 16.1 (1990), pp. 22–29. URL: https://aclanthology.org/J90-1003.

[28] José A. Villegas and Emmanuel D. Levy. "A unified statistical potential reveals that amino acid stickiness governs nonspecific recruitment of client proteins into condensates". In: *Protein Science* 31.7 (2022). ISSN: 0961-8368, 1469-896X. DOI: 10.1002/pro.4361.
